# Methodological drivers, reporting completeness, and transferability in HPV vaccination economic evaluations: A scoping review

**DOI:** 10.1371/journal.pone.0360732

**Published:** 2026-10-14

**Authors:** Adnan Kisa, Sezer Kisa

**Affiliations:** 1 School of Health Sciences, Kristiania University of Applied Sciences, Oslo, Norway; 2 Department of Nursing and Health Promotion, Faculty of Health Sciences, Oslo Metropolitan University, Oslo, Norway; University of Bologna: Universita di Bologna, ITALY

## Abstract

**Background:**

Human papillomavirus (HPV) vaccination is widely reported as cost-effective, but reported incremental cost-effectiveness ratios (ICERs) are shaped by model structure, assumptions, comparator choice, vaccine price, threshold selection, and local screening context. Previous reviews have summarized cost-effectiveness findings but have less often examined why conclusions differ across settings.

**Objective:**

To map methodological evolution, drivers of variation, reporting completeness, methodological credibility, and transferability in HPV vaccination economic evaluations.

**Methods:**

A scoping review was conducted using the methodological framework proposed by Arksey and O’Malley, refined by Levac et al., and informed by JBI guidance for scoping reviews. Reporting followed PRISMA-ScR, and the protocol was preregistered on the Open Science Framework (OSF). PubMed, Scopus, PsycINFO, Embase, Web of Science, and CINAHL were searched from January 1, 2003, to December 31, 2025, with a final search conducted on January 2, 2026. Forty-four records were mapped. Thirty-three full comparative economic evaluations formed the primary synthesis; 11 partial or non-standard economic evidence records were retained as contextual evidence. The Consolidated Health Economic Evaluation Reporting Standards 2022 (CHEERS 2022) checklist was applied retrospectively to assess reporting completeness, and a descriptive structured appraisal was used to examine methodological credibility across six health-economic domains. A Welte-informed assessment was used to examine target-context contextualization.

**Results:**

The evidence base evolved from early static cohort models to dynamic transmission and individual-based approaches, while static models remained common in LMIC-focused standardized-tool analyses. The main drivers of variation were vaccine price, discounting, herd-immunity assumptions, cross-protection assumptions, and threshold selection. Reporting completeness varied and did not necessarily correspond to methodological credibility. Relevance to a study’s own decision setting did not imply that its ICER could be transferred to other countries. Eleven contextual records showed emerging policy questions around delivery costs, fiscal consequences, uptake, risk-benefit framing, and non-ICER-based decision support.

**Conclusion:**

The economic value reported for HPV vaccination depends on the assumptions, comparators, vaccine prices, thresholds, and local screening contexts used in each evaluation rather than on the vaccine alone. Future evaluations should explicitly justify the choice of comparator, model structure, vaccine-price assumptions, discount rates, thresholds, uncertainty analysis, and transferability assumptions. Although formal cost-effectiveness analyses of single-dose HPV vaccination have begun to emerge, evidence remains limited, particularly regarding durability, waning, delivery feasibility, gender-neutral vaccination in LMICs, and country-specific evaluations in underrepresented regions.

## Introduction

Human papillomavirus (HPV) infection is one of the most prevalent sexually transmitted infections worldwide and remains the dominant etiological factor for cervical cancer, alongside other anogenital and oropharyngeal cancers [1]. Globally, cervical cancer ranks as the fourth most common cancer among women, responsible for over 600,000 new cases and 340,000 deaths annually, with approximately 90% of deaths occurring in low- and middle-income countries [2]. The World Health Organization (WHO) recognizes HPV vaccination as a central pillar of its global strategy to eliminate cervical cancer as a public health problem, articulated in 2020 through the 90-70-90 targets: 90% of girls fully vaccinated against HPV by age 15, 70% of women screened with a high-precision test, and 90% of women identified with cervical disease receiving treatment [1]. Achievement of these targets requires evidence-based decision support for resource allocation across heterogeneous health system contexts.

Since the licensure of the first prophylactic HPV vaccines in 2006, vaccination programs have expanded across high- and low-income settings. The vaccine landscape has undergone a substantial transformation, encompassing bivalent HPV 16/18, quadrivalent HPV 6/11/16/18, and nonavalent HPV 6/11/16/18/31/33/45/52/58 formulations [3–6]. Parallel programmatic developments have included the transition from three-dose to two-dose schedules, the expansion of national programs to include boys, catch-up vaccination of older adolescents, and the WHO Strategic Advisory Group of Experts 2022 endorsement of a single-dose schedule for primary target populations [5,7,8].

Economic evaluation of HPV vaccination is methodologically complex. The natural history of HPV infection involves long latency periods between vaccination and prevention of cervical cancer, requiring time horizons of 70–100 years and sensitivity to discount rates [3,6,9]. HPV transmission dynamics imply that vaccination of one population subgroup confers indirect herd protection on unvaccinated individuals, a phenomenon usually requiring dynamic transmission models when it is central to the decision problem [6,10,11]. Vaccine efficacy varies by HPV strain, disease site, and sex. Comparator strategies also differ substantially, including bivalent, quadrivalent, and nonavalent vaccines, girls-only and gender-neutral programs, and one-dose, two-dose, and three-dose schedules. These differences contribute to substantial variation in reported incremental cost-effectiveness ratios (ICERs) [3,6,9].

Whether HPV vaccination ICERs can be transferred across jurisdictions remains uncertain because studies differ in model structure, local parameterization, comparator choice, vaccine price scenarios, threshold selection, and screening context [3,6,9,12]. In addition to economic and epidemiological parameters, HPV vaccination policy is shaped by social acceptability, cultural context, and trust. A previous scoping review of HPV vaccine acceptance in Islamic countries found that religious beliefs, concerns about vaccine safety and fertility, perceived encouragement of sexual activity, and perceptions of halal or haram vaccine ingredients may influence acceptance [13]. These demand-side factors are not always incorporated into economic models, yet they are relevant for interpreting uptake assumptions, implementation feasibility, and transferability across settings [12,13]. As observed in the mapped records, industry sponsorship was a recurring feature of the HPV vaccine economic evaluation literature, with frequent involvement of GlaxoSmithKline and Merck/MSD or its European partner Sanofi Pasteur MSD. Reporting transparency is important because decision-makers require sufficient information to assess assumptions, comparators, uncertainty, and transferability. However, reporting completeness should be distinguished from methodological credibility [14].

Existing reviews have established that HPV vaccination is frequently cost-effective, particularly at low vaccine prices and in high-burden settings. Mahumud et al. (2020) [4] systematically reviewed 9-valent HPV vaccine cost-effectiveness analyses. Ng et al. (2018) [5] reviewed cost-effectiveness studies of 9-valent, gender-neutral, and multi-age-cohort vaccination. Segal and Rashid (2023) [15] conducted a targeted review of HPV economic evaluation reviews from 2012 to 2020. Ortega-Sanchez et al. (2008) [16] reviewed early US HPV CEAs in the immediate post-licensure period. These reviews have mapped whether HPV vaccination is cost-effective under specific assumptions or vaccine valencies. They have not, however, systematically mapped why economic conclusions differ across studies, how recurring methodological drivers shape reported ICERs, how reporting completeness compares with methodological credibility, how recurring model families and product-comparison frameworks are distributed across the evidence base, or how local contextualization affects transferability. These dimensions remain unmapped.

This scoping review contributes to the literature by shifting attention from whether HPV vaccination is cost-effective to why reported economic value differs across settings. It maps the evolution of HPV vaccination economic evaluation across vaccine valencies and program strategies, identifies recurring methodological drivers of variation, distinguishes reporting completeness from methodological credibility, and examines how target-context contextualization affects the interpretation and transferability of ICERs.

### Research aim and questions

The aim of this scoping review is to examine how methodological design choices, reporting practices, model families, and contextual factors shape the interpretation, comparability, and policy relevance of HPV vaccination economic evaluations.

RQ1: How have modeling approaches in HPV vaccination economic evaluations evolved over time?

RQ2: Which methodological and contextual factors recur as drivers of variation in reported economic conclusions?

RQ3: How complete is reporting according to CHEERS 2022, and how does reporting completeness compare with the structured appraisal of methodological credibility?

RQ4: How well are studies contextualized to local decision settings, and what factors limit the transferability of findings across countries?

## Methods

### Study design and framework

This scoping review followed the methodological framework proposed by Arksey and O’Malley (2005) [17], refined by Levac et al. (2010) [18], and informed by JBI guidance for scoping reviews [19]. Reporting followed PRISMA-ScR [20]. A protocol was developed a priori and preregistered in the Open Science Framework (OSF): https://osf.io/an48f.

### Eligibility criteria and evidence categories

For transparency, eligible records were classified into three evidence categories. Mapped records referred to all included records that met the review eligibility criteria. Full comparative economic evaluations referred to cost-effectiveness analyses, cost-utility analyses, and formal cost-benefit analyses that compared alternative strategies in terms of both costs and consequences and provided a complete economic evaluation framework. Contextual records referred to partial or policy-relevant non-standard economic analyses that were charted for scoping breadth but not pooled with the primary synthesis. These included cost-consequence analyses, fiscal net present value analyses, coverage-uptake impact analyses, decision-tree risk-benefit frameworks, number-needed-to-vaccinate analyses, simplified or hybrid cost-benefit analyses, cost-of-delivery studies, and epidemiological transmission models without economic outcomes.

Records were excluded if they: (1) focused exclusively on cervical cancer screening, diagnosis, or treatment without a vaccination component; (2) reported only cost-of-illness without an intervention comparator; (3) constituted non-economic or qualitative research; (4) were systematic or narrative reviews of economic evaluations, theses, conference abstracts, or editorials; (5) evaluated non-HPV vaccines; (6) were methodology-comparison papers using HPV only as an illustrative case; or (7) addressed adjuvant secondary-prevention settings rather than primary HPV vaccination. Only peer-reviewed English-language publications were included; gray literature, HTA reports, theses, conference abstracts, and editorials were excluded.

### Search strategy

Six databases were searched: PubMed, Scopus, PsycINFO, Embase, Web of Science, and CINAHL. The search covered January 1, 2003, to December 31, 2025, with a final search conducted on January 2, 2026, to ensure completeness. Search strategies combined controlled vocabulary terms and free-text keywords structured around three search blocks aligned with the Population–Concept–Context (PCC) framework: HPV vaccination, economic evaluation, and program/context terms. Database-specific Boolean search strategies and the administrative/search-filter criteria applied before title and abstract screening are provided in S6 Table. Record counts per database, the de-duplication and screening cascade, and the PRISMA 2020 flow diagram adapted for scoping review study selection are reported in Section 4.1 and Fig 1.

**Fig 1:**
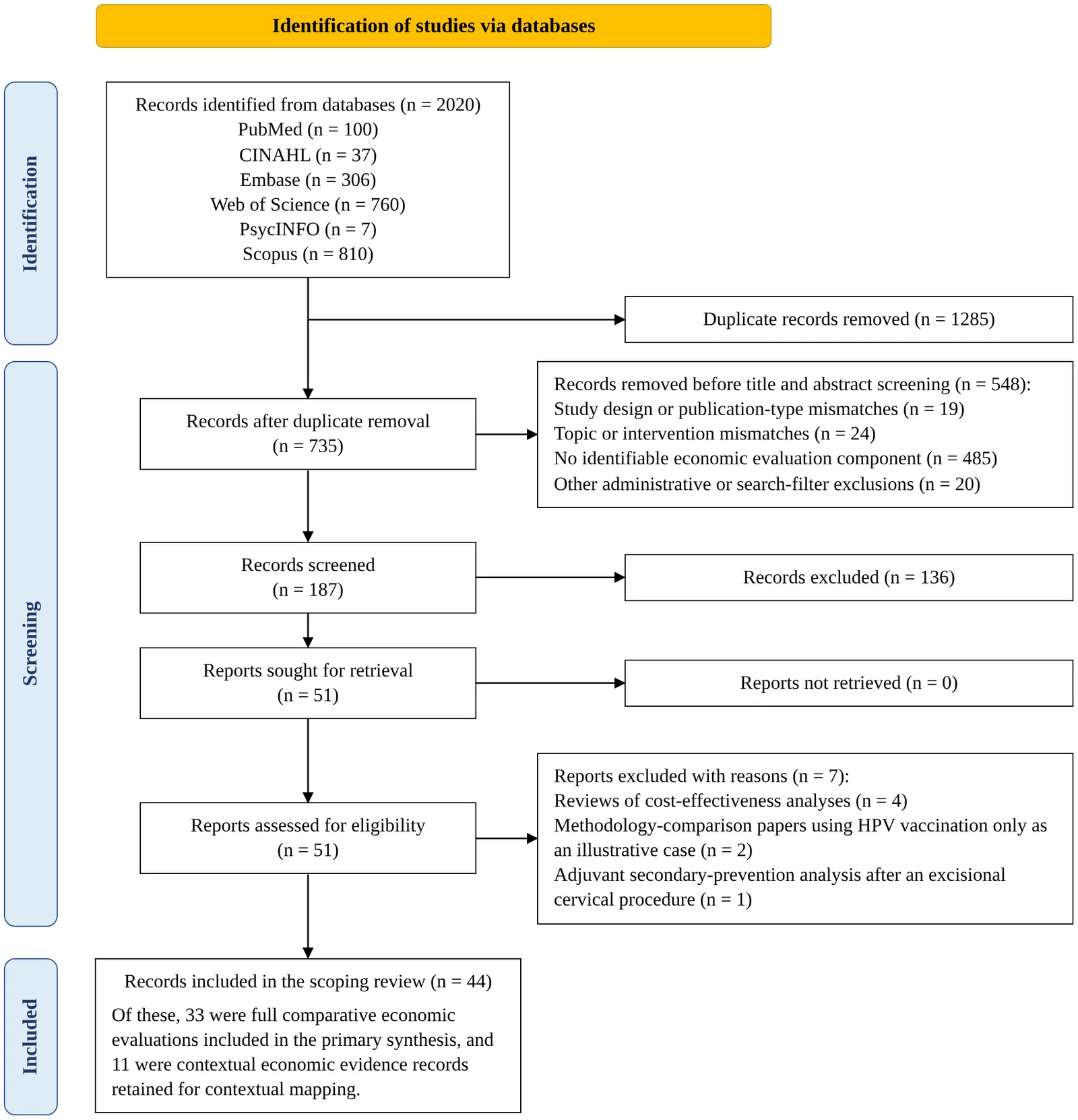
PRISMA 2020 flow diagram adapted for the scoping review study selection process.

### Study selection and data charting

All retrieved records were imported into EndNote 21 for de-duplication and then transferred to Covidence for systematic screening. Before title and abstract screening, a set of records was removed because they met predefined administrative or search-filter criteria rather than substantive eligibility criteria. These pre-screening exclusions were identified during record management in EndNote 21 and Covidence and were verified manually against each record’s title, abstract, keywords, publication type, and database metadata; no automated text-mining or machine-classification algorithm was used to assign them. Four categories were applied: study design or publication-type mismatches (for example, editorials, letters, notes, and conference items not already removed by database-level publication-type filters); topic or intervention mismatches (records outside HPV vaccination); records with no identifiable economic evaluation component (no cost, cost-effectiveness, cost-utility, cost-benefit, budget-impact, or related economic-evaluation terms in the title, abstract, or keywords); and other administrative or search-filter exclusions not assignable to the preceding three categories (for example, records retained through database-export or search-filter artifacts). Records were separated at this stage only when they were clearly ineligible by record type, topic, or the complete absence of an economic component; any record with potential economic evaluation content was retained and carried forward to title and abstract screening. The number of records removed in each category is reported in Section 4.1, and the criteria are summarized in S6 Table. The remaining records were independently screened by two reviewers at the title and abstract level. Disagreements were resolved through discussion until a consensus was reached. Full-text reports were independently assessed for eligibility by two reviewers, with disagreements resolved through discussion until consensus was reached.

Data extraction followed a standardized charting form developed in Microsoft Excel and piloted on the first five included records. The form was organized into five consolidated extraction tables, with rows representing records and columns representing variables, and is provided as S1–S5 Tables.

Two reviewers independently extracted data for the full comparative economic evaluations included in the CHEERS, methodological credibility, and transferability assessments. Contextual records were charted using the same extraction framework for bibliographic, intervention, funding, methodological, and policy-relevance information, but were not included in the primary methodological credibility or Welte-informed transferability synthesis [12]. In the supplementary tables, contextual records were retained for descriptive mapping but distinguished from full comparative economic evaluations to avoid conflating partial economic evidence with formal ICER-based synthesis. Inter-reviewer agreement was not converted into a quantitative score, and disagreements were resolved through discussion until consensus was reached.

### Data synthesis and analysis

#### Methodological evolution and drivers of variation.

Methodological evolution analysis focused on the full comparative economic evaluations. Time-period stratification was used to examine changes in modeling approaches across three periods: 2004–2010, 2011–2017, and 2018–2025. For RQ2, a methodological or contextual factor was classified as a recurring driver of variation when it was identified by study authors as influential in deterministic or probabilistic sensitivity analysis in at least three studies, or when it repeatedly explained divergent conclusions across studies evaluating comparable vaccination strategies or similar country contexts.

#### Reporting completeness assessment.

Full economic evaluations were assessed against the 28-item CHEERS 2022 checklist as the primary reporting-completeness analysis [14]. CHEERS 2022 is a reporting checklist that assesses whether key information is reported; it does not assess methodological quality. Items were categorized as fully reported (F), partially reported (P), not reported (N), or not applicable (NA). Reviewer-defined CHEERS bands were used for descriptive comparison only: high (≥80%), moderate (60–79%), and low (<60%) for the applicable items fully reported. These bands are not part of CHEERS 2022. Item 4, Health Economic Analysis Plan, was coded as N rather than NA for the majority of pre-2022 records; NA was reserved for non-CEA contextual records where CHEERS was only partially applicable. CHEERS coding for contextual records was retained in the supplementary material only and was not included in the primary CHEERS synthesis. Because CHEERS 2022 was published in 2022, applying it retrospectively to studies published before that date assessed completeness of retrospective reporting rather than reporting failure relative to contemporaneous standards.

#### Structured methodological credibility appraisal.

Because CHEERS 2022 does not evaluate whether the model structure, comparator, uncertainty analysis, or threshold choice was appropriate for the decision problem, a separate structured methodological credibility appraisal was conducted. Methodological credibility was appraised across six domains: comparator appropriateness, model structure suitability, local calibration and validation, herd-immunity modeling where relevant, uncertainty analysis, and threshold justification. Because the purpose of the review was to map how methodological choices shape interpretation and transferability, this structured appraisal was used descriptively and was not used to exclude records, produce a risk-of-bias score, or generate an overall quality ranking. Each domain was rated Adequate, Partial, Limited, or NA where applicable, using predefined criteria summarized in this section; aggregate results are reported in Table 4. Two reviewers applied the rubric independently, and disagreements were resolved through discussion until consensus was reached.

#### Transferability assessment.

The Welte framework was used to identify transferability barriers and to describe the degree of target-context contextualization [12]. Target-context contextualization refers to how strongly a study was parameterized for its own decision setting. It should not be interpreted as evidence that the ICER is transferable to other countries. Formal Welte-informed assessment was applied only to full comparative economic evaluations. For contextual records, local data relevance and policy-context information were charted descriptively. Records were classified as high in contextualization when several key target-context dimensions were substantially reflected, such as local epidemiology, cost data, demographics or behavioral inputs, screening context, vaccine price, and/or locally relevant thresholds. Records were classified as moderate when some local inputs were used but were combined with key imported assumptions or regional extrapolations, and low when local data were limited.

#### Recurring model families and product-comparison frameworks.

A model lineage was defined as a group of studies sharing at least two features: a common sponsor or manufacturer portfolio, a recurring author or model development group, an explicitly adapted model structure, recurring vaccine product comparisons, or similar assumptions about vaccine efficacy and cross-protection. Lineages were identified descriptively to map recurring modeling patterns across the evidence base. This analysis was not designed to test funding-related bias or to infer causal relationships between sponsorship, model structure, and study conclusions.

## Results

### Included records and evidence categories

The database searches identified 2,020 records: Scopus (n = 810), Web of Science (n = 760), Embase (n = 306), PubMed (n = 100), CINAHL (n = 37), and PsycINFO (n = 7). After removing 1,285 duplicates, 735 records remained. Of these, 548 records were removed before title and abstract screening using predefined administrative and search filter criteria. These comprised study design or publication type mismatches (n = 19), topic or intervention mismatches (n = 24), records with no identifiable economic evaluation component (n = 485), and other administrative or search filter exclusions not assigned to the preceding categories (n = 20). The remaining 187 records were screened by title and abstract, and 136 records were excluded. A total of 51 reports were sought for retrieval, and all were retrieved and assessed for eligibility. Seven reports were excluded at the full-text stage, leaving 44 records included in the final scoping review. The study selection process is shown in Fig 1. The seven full-text exclusions comprised four reviews of cost-effectiveness analyses, two methodology-comparison papers that used HPV vaccination only as an illustrative case, and one adjuvant secondary prevention analysis of HPV vaccination after an excisional cervical procedure.

Unless otherwise stated, the main analyses refer to the 33 full comparative economic evaluations. The 11 contextual records are described separately for scoping breadth. The mapped evidence base comprised 44 records published between 2004 and 2025, covering more than 25 distinct country contexts plus 5 multi-country regional analyses across all six WHO regions. Thirty-three full comparative economic evaluations formed the primary synthesis sample. Eleven records reported partial or non-standard economic evidence and were therefore summarized as contextual evidence rather than synthesized with ICER-based evaluations. Table 1 summarizes the distribution of included records by evidence category and outcome metric.

**Table 1:** Distribution of included records by evidence category and outcome metric (n = 44).

Evidence category	Outcome metric/ analytic form	n	Role in synthesis
Full comparative economic evaluation	QALY-based cost-utility analysis	24	Primary synthesis
Full comparative economic evaluation	DALY-, life-year-, or case-based cost-effectiveness analysis	9	Primary synthesis
Partial or non-standard economic evidence	Cost-consequence, cases-prevented, NNV, fiscal NPV, hybrid CBA, coverage-uptake impact, decision-tree risk-benefit, cost-of-delivery, or epidemiological transmission model	11	Contextual mapping

Note: The outcome metric is reported separately from the evaluation type because cost-effectiveness and cost-utility analyses use different outcome conventions in high-income and LMIC/UMIC contexts. Per-record bibliographic information, country, income group, funding type, vaccine strategy, and model type for all 44 records are provided in S1 Table.

Of the 33 full comparative economic evaluations, 9 records were published in 2004–2010, 12 in 2011–2017, and 12 in 2018–2025. Industry-funded studies accounted for 16 records, and the remaining 17 were non-industry. LMIC and UMIC settings were represented by two records in 2004–2010 (Mexico and Hungary), ten in 2011–2017 (two from Thailand, two from Indonesia, and one each from Colombia, Malaysia, and Chile, together with the multi-country CEECA, sub-Saharan Africa, and EMENA analyses), and six in 2018–2025 (Iran, Morocco, India, Türkiye, Thailand, and Nigeria).

### Methodological evolution

Among the 33 full comparative economic evaluations, three time periods were examined. Phase 1, covering 2004–2010, included 9 records and was characterized mainly by static Markov cohort models (n = 6), with fewer dynamic transmission models (n = 2) and one individual-based microsimulation. Phase 2, covering 2011–2017, included 12 records, comprising static cohort models (n = 6), dynamic transmission models (n = 3), and microsimulation models (n = 3). During this period, LMIC and UMIC evaluations expanded through DALY-based analyses, regional modeling, and two-tier threshold approaches. Phase 3, covering 2018–2025, included 12 records: static cohort models (n = 8), dynamic transmission models (n = 3), and an individual-based MCMC microsimulation. Static cohort models remained common, particularly in standardized-tool LMIC analyses and in recent single-dose evaluations, while dynamic models were used in several gender-neutral and 9-valent evaluations (Table 2).

**Table 2:** Methodological features by phase across full comparative economic evaluations (n = 33).

Methodological feature	Phase 1 (2004–2010), n = 9	Phase 2 (2011–2017), n = 12	Phase 3 (2018–2025), n = 12
Predominant model type	Static Markov cohort (n = 6), dynamic transmission (n = 2), microsimulation (n = 1)	Static cohort (n = 6), dynamic transmission (n = 3), microsimulation (n = 3)	Static cohort (n = 8), dynamic transmission (n = 3), individual-based MCMC (n = 1)
Herd immunity explicitly modeled	2/9	3/12	4/12
Vaccine valency	Predominantly bivalent and quadrivalent, with pre-licensure HPV 16/18 modeling	Quadrivalent dominant, with transition toward 9-valent evaluation	9-valent dominant, with head-to-head bivalent, quadrivalent, and 9-valent comparisons. Single-dose modeling emerged in recent full evaluations.
Time horizon	Lifetime, usually 70–100 years	Lifetime, with age-specific scenarios	Lifetime, with single-dose, two-dose, and threshold-dose analyses
Discount rate (base case)	3% for costs and outcomes	3% standard, with lower outcome discounting in later UK evaluations	1.5%–5%, with sensitivity analyses prominent
Probabilistic sensitivity analysis	1/9 (formal PSA)	5/12 (formal PSA)	6/12 (formal PSA); 1 Bayesian calibration uncertainty
Cross-protection methodology	Cross-protection is generally excluded from the base case; type-specific or “irrespective of type” coding is limited to early product-comparison work	Cross-protection is generally excluded from the base case. Broader efficacy coding introduced in product-comparison studies	Cross-protection is generally excluded from the base case. Broader efficacy coding limited to product-comparison studies
LMIC/UMIC representation	2 of 9 studies: Mexico (n = 1), Hungary (n = 1)	10 of 12 studies: Thailand (n = 2), Indonesia (n = 2), Colombia (n = 1), Malaysia (n = 1), Chile (n = 1), CEECA region (n = 1), Sub-Saharan Africa (n = 1), and EMENA region (n = 1)	6 of 12 studies: Iran (n = 1), Morocco (n = 1), India (n = 1), Türkiye (n = 1), Thailand (n = 1), and Nigeria (n = 1)

Note: Table values are derived from the 33 full comparative economic evaluations included in the primary synthesis. Detailed per-record model structure, transmission approach, perspective, time horizon, discount rate, PSA status, and key assumptions are provided in S2 Table.

### Drivers of economic variation

Five recurring methodological drivers of variation were identified through cross-study comparison using the operational definition described in Section 3.5.1: vaccine price, discount rate, herd-immunity modeling, vaccine efficacy and cross-protection assumptions, and threshold selection. The inclusion of indirect productivity costs was identified as a recurring driver, particularly in CBA, the societal perspective, and fiscal frameworks. Analytic perspectives also varied across studies, ranging from healthcare payer and government perspectives to limited societal, full societal, fiscal, and individual risk-benefit perspectives. These differences affected whether productivity costs, patient costs, and broader fiscal consequences were included. In the two recent single-dose evaluations, assumptions about the duration of protection, waning, and schedule-specific vaccine effectiveness were policy-relevant but were not classified as recurring drivers because they appeared in fewer than 3 full comparative economic evaluations (Table 3).

**Table 3:** Methodological drivers of variation in HPV economic outcomes, with illustrative examples.

Driver	Mechanism	Illustrative examples
Vaccine price	Vaccine acquisition price was reported as a key sensitivity parameter across LMIC and UMIC settings. It often interacted with the discount rate and threshold choice.	Praditsitthikorn et al. (2011) [21], Thailand: vaccination was cost-effective only when the vaccine price was substantially reduced. Aponte-González et al. (2013) [22], Colombia: explicit vaccine price thresholds for cost-effectiveness were reported. Kim et al. (2013a) [23], SSA, and Kim et al. (2013b) [24], EMENA: vaccine price thresholds were reported across multi-country regional analyses.
Discount rate	Lower outcome discount rates improved ICERs because HPV vaccination benefits accrue decades after vaccination costs.	Datta et al. (2019) [10], United Kingdom: gender-neutral vaccination was cost-effective at 1.5% but not at 3.5%. Owusu-Edusei et al. (2022) [25], United Kingdom: the ICER for switching from quadrivalent to 9-valent vaccination was substantially lower at 1.5% than at 3.5%. Şahin and Karabey (2025) [26], Türkiye: discount rate was identified as the most influential parameter in tornado analysis.
Herd immunity inclusion	Dynamic transmission models incorporating herd effects produced different ICERs than static cohort models for the same vaccination program.	Schobert et al. (2012) [27], Germany: dynamic transmission modeling produced an ICER of €5,525 per QALY. Chesson et al. (2018) [28], United States: dynamic transmission modeling found that harmonization of male catch-up vaccination to age 26 had an ICER of US$228,800 per QALY, above conventional US willingness-to-pay thresholds, despite herd effects.
Vaccine efficacy and cross-protection assumptions	Head-to-head product comparisons using vaccine efficacy, irrespective of HPV type, assumed broader protection than type-specific cross-protection coding.	Demarteau and Standaert (2010) [29], France, Ireland, and Italy: under base-case assumptions, the quadrivalent vaccine dominated the bivalent vaccine in all three countries. The conclusion reversed when minimum cross-protection was assumed for the bivalent vaccine. Lee et al. (2019) [30], South Korea, and Tang et al. (2019) [31], Taiwan, also used head-to-head product-comparison frameworks.
Cost-effectiveness threshold	WHO-CHOICE GDP-based thresholds produced different conclusions from country-specific willingness-to-pay, HTA, or opportunity-cost benchmarks.	Yaghoubi et al. (2018) [32], Iran: ICERs of US$15,205 per DALY from the government perspective and US$14,999 per DALY from the societal perspective were both above the threshold based on three times GDP per capita, US$14,289. Vargas Parada and Veerman (2016) [33], Chile: the ICER of US$138,269 per DALY for HPV vaccination as anal cancer prevention in HIV-positive men was above the threshold based on three times GDP per capita, US$55,758. Şahin and Karabey (2025) [26], Türkiye: both WHO-CHOICE and Ochalek opportunity-cost thresholds were applied.

Note: Detailed model structure, perspective, discounting, base case ICERs, thresholds, and sensitivity drivers are provided in S2 and S3 Tables.

### CHEERS reporting completeness

Among the 33 full economic evaluations, retrospective CHEERS 2022 assessment identified 15 records with high reporting completeness, 16 with moderate completeness, and 2 with low completeness, using reviewer-defined bands of high ≥80%, moderate 60–79%, and low < 60%. This corresponded to 45%, 48%, and 6% of the full economic evaluations, respectively. In descriptive comparison, industry-funded records showed a mean reporting completeness of 73.0% (n = 16), compared with 81.3% among non-industry records (n = 17). Mean reporting completeness was 72.0% in 2004–2010 (n = 9), 76.8% in 2011–2017 (n = 12), and 81.6% in 2018–2025 (n = 12).

Items related to title, abstract, background, study population, setting and location, comparators, perspective, time horizon, and discount rate were generally well reported. Reporting was more variable for Item 4, Health Economic Analysis Plan, which was mostly absent in pre-2022 studies, as well as Item 17, analytics and assumptions justification, Item 18, characterizing heterogeneity, Item 19, characterizing distributional effects, Item 20, characterizing uncertainty, and Item 21, approach to engagement.

### Structured methodological credibility appraisal

The structured methodological credibility appraisal showed variation across the six assessed domains. Comparator appropriateness and model structure suitability were the strongest areas, with 23 records rated as adequate for comparator appropriateness and 21 for model structure suitability. Local calibration and validation were more variable, with 16 records rated adequate, 13 partial, and 4 limited, particularly reflecting variation in standardized-tool LMIC studies and regional modeling studies that relied on standardized or extrapolated inputs. Uncertainty analysis and threshold justification were also uneven. Formal probabilistic sensitivity analysis was reported in a minority of records, mainly in high-income and microsimulation studies, whereas several LMIC-focused or standardized-tool analyses relied mainly on deterministic sensitivity analysis (Table 4).

**Table 4:** Structured descriptive appraisal of methodological credibility across full comparative economic evaluations (n = 33).

Domain	Adequate, n (%)	Partial, n (%)	Limited, n (%)	Not applicable, n (%)	Key observations
Comparator appropriateness	23 (70%)	9 (27%)	1 (3%)	0 (0%)	Most studies used policy-relevant comparators, including no vaccination, screening alone, or the current program. Head-to-head comparisons of bivalent versus quadrivalent products were used in narrower product comparison frameworks.
Model structure suitability	21 (64%)	10 (30%)	2 (6%)	0 (0%)	Dynamic transmission models were used where herd immunity effects were central to the policy question. Static cohort models remained common in CERVIVAC- and PRIME-based LMIC analyses, partly reflecting data constraints.
Local calibration and validation	16 (48%)	13 (39%)	4 (12%)	0 (0%)	Stronger calibration and validation were observed in records that used local epidemiological, genotype distribution, behavioral, screening, surveillance, cost, or utility data. More limited calibration was observed mainly in standardized-tool LMIC studies and regional modeling studies that relied on standardized, extrapolated, or externally derived inputs.
Herd immunity modeling, where relevant	9 (27%)	6 (18%)	1 (3%)	17 (52%)	Adequate herd immunity modeling focused on dynamic transmission and individual-based transmission models. Static cohort and microsimulation analyses that explicitly excluded herd immunity did not model indirect effects. A limited rating was applied where herd effects were potentially central to the policy question but were not modeled. Not applicable was used for evaluations where herd effects were not central to the decision problem, such as girls-only routine vaccination.
Uncertainty analysis adequacy	15 (45%)	10 (30%)	8 (24%)	0 (0%)	Formal probabilistic sensitivity analysis was reported in 12 records. A smaller group incorporated calibration parameter set uncertainty or Bayesian calibration uncertainty. The remaining records mainly relied on deterministic one-way, multivariate, or scenario sensitivity analyses, including several standardized-tool LMIC evaluations and some recent high-income country analyses.
Threshold justification	17 (52%)	13 (39%)	3 (9%)	0 (0%)	Country-specific HTA thresholds were explicitly justified in several high-income settings, including the UK, US, Netherlands, and Italy. In several LMIC and UMIC studies, WHO-CHOICE thresholds based on one to three times GDP per capita were applied, sometimes without strong country-specific justification. More recent work also incorporated opportunity cost thresholds, including the comparison of WHO-CHOICE and Ochalek based thresholds in the Türkiye evaluation.

**Note:** Domain criteria are described in Methods Section 3.5.3. Not applicable was used only for the herd immunity domain, where the policy question did not centrally require herd effects modeling, such as girls-only routine vaccination. Other domains applied to all 33 full comparative economic evaluations. Methodological credibility appraisal is reported at the aggregate domain level in this table. Per-record CHEERS item level assessment is provided in S5 Table.

### Target-context contextualization and transferability

Among the 33 full economic evaluations, target-context contextualization did not always align with CHEERS reporting completeness. Several records with high CHEERS completeness were rated only moderate for contextualization because they relied on regional modeling, extrapolated cost data, or product-comparison frameworks. Conversely, some records with moderate CHEERS completeness had high contextualization because they used country-specific screening practice, epidemiology, surveillance, cost, or policy inputs. The main transferability barriers were therefore not only reporting gaps, but also differences in screening organization, cost inputs, vaccine-product assumptions, calibration, and threshold choice (Table 5).

**Table 5:** CHEERS reporting completeness and target-context contextualization across full economic evaluations (n = 33).

Study	CHEERS reporting completeness	CHEERS band	Target-context contextualization	Main credibility concern or transfer barrier
Goldie et al. 2004 [3]	85.2%	High	High	US-specific epidemiology and screening, with pre-licensure modeling assumptions
Boot et al. 2007 [34]	44.4%	Low	High	Friction-cost productivity assumptions specific to the Dutch context
Elbasha et al. 2007 [6]	70.4%	Moderate	High	US-specific epidemiology and foundational dynamic transmission framework
Kulasingam et al. 2008 [35]	81.5%	High	High	UK NHS-specific screening practice
Coupé et al. 2009 [36]	74.1%	Moderate	High	Dutch organized screening assumptions
Kim et al. 2009 [9]	81.5%	High	High	US-specific demographic and screening; older-women context-specific
Reynales-Shigematsu et al. 2009 [37]	70.4%	Moderate	Moderate	Adapted from Kulasingam UK framework with limited local recalibration
Dasbach et al. 2010 [38]	66.7%	Moderate	High	Hungarian-specific epidemiology; limited cross-protection coding
Demarteau and Standaert 2010 [29]	74.1%	Moderate	Moderate	Multi-jurisdictional model; conclusions sensitive to cross-protection assumption
Praditsitthikorn et al. 2011 [21]	88.9%	High	High	Local Thai epidemiology, costs, and thresholds
Schobert et al. 2012 [27]	63.0%	Moderate	High	German SHI-specific perspective
Termrungruanglert et al. 2012 [39]	70.4%	Moderate	Moderate	Narrower comparator framework than another Thai policy evaluation
Aponte-González et al. 2013 [22]	85.2%	High	High	Colombian local data with explicit price thresholds
Berkhof et al. 2013 [40]	81.5%	High	Moderate	CEECA regional 2-tier; country-level estimates limited
Kim et al. 2013a [23]	82.1%	High	Moderate	Multi-country SSA regional; cost data extrapolation
Kim et al. 2013b [24]	82.1%	High	Moderate	Multi-country EMENA regional; cost data extrapolation
Aljunid et al. 2016 [41]	74.1%	Moderate	Moderate	Malaysian local cost data; partial local utility values
Setiawan et al. 2016a [42]	75.0%	Moderate	High	Indonesian local primary cost data
Vargas Parada and Veerman 2016 [33]	85.7%	High	Moderate	HIV-positive male-specific; colorectal cancer cost as proxy for anal cancer
Kosen et al. 2017 [43]	51.9%	Low	Moderate	Expert-informed local estimates
Mennini et al. 2017 [44]	81.5%	High	High	Italian local 9-valent transition data
Chesson et al. 2018 [28]	92.9%	High	High	US comprehensive surveillance
Yaghoubi et al. 2018 [32]	81.5%	High	Moderate	CERVIVAC standardized LMIC tool, with limited screening modeling and limited uncertainty analysis
Datta et al. 2019 [10]	96.4%	High	High	UK NATSAL behavioral data; methodologically advanced
Lee et al. 2019 [30]	74.1%	Moderate	Moderate	Korean local data; product-comparison framework
Messoudi et al. 2019 [45]	76.9%	Moderate	Moderate	Spanish ICO methodology adapted to Morocco; partial local
Tang et al. 2019 [31]	85.7%	High	Moderate	Taiwan local data; product-comparison framework
Chauhan et al. 2020 [46]	89.3%	High	High	Indian primary local data with EQ-5D and 9-strategy screening
Owusu-Edusei et al. 2022 [25]	74.1%	Moderate	High	UK 4v → 9v transition. Deterministic sensitivity analysis only
Palmer et al. 2023 [11]	75.0%	Moderate	High	Japan-specific Bayesian metapopulation; calibration uncertainty only
Termrungruanglert et al. 2024 [57]	77.8%	Moderate	High	Static Markov cohort; cross-protection and herd immunity not modeled. Strong Thai-specific calibration; provider perspective only
Şahin and Karabey 2025 [26]	77.8%	Moderate	High	PRIME static framework limits screening modeling and herd immunity. Deterministic sensitivity analysis only
Gao et al. 2025 [47]	77.8%	Moderate	High	Static Markov cohort; herd immunity not captured (acknowledged). Strong Nigeria real-world cost and coverage data; GDP-based threshold

Note: Table values are derived from the included records and the record-level extraction tables. Records are ordered chronologically by publication year. The CHEERS item-level assessment is provided in S5 Table. Target-context contextualization summaries are provided in S4 Table. Methodological credibility appraisal is reported at the aggregate domain level in Table 4.

### Recurring model families and product-comparison frameworks

A recurring head-to-head comparison framework was identified using the operational lineage definition described in Section 3.5.5. This framework employed static Markov modeling with a “vaccine efficacy irrespective of HPV type” approach in product comparisons evaluating AS04-HPV-16/18v, Cervarix, against quadrivalent or nonavalent comparators. Among the full economic evaluations, this framework appeared in Demarteau 2010 [29], France, Ireland, and Italy, Lee 2019 [30], South Korea, and Tang 2019 [31], Taiwan. Among the contextual records, Capri 2011 [48], Italy, and Bonanni 2015 [49], Italy, formed part of the same model family using cost-consequence and cases-prevented frameworks, respectively. This model family was observed mainly among GSK-funded head-to-head product-comparison records.

A second recurring model family was the Elbasha-Dasbach-Insinga dynamic transmission lineage, originally developed for the quadrivalent vaccine and later extended for 9-valent transition analyses. Among the full economic evaluations, records in this lineage included Elbasha 2007 [6], United States, Dasbach 2010 [38], Hungary, Kosen 2017 [43], Indonesia, Mennini 2017 [44], Italy, Owusu-Edusei 2022 [25], United Kingdom, and Palmer 2023 [11], Japan. Among the contextual records, Bresse 2013 [50], France, was a cost-consequence analysis in the same model family. This dynamic transmission family was observed mainly in records funded by Merck/MSD or Sanofi Pasteur MSD and used model structures explicitly adapted from earlier analyses in the same lineage across multiple jurisdictions.

Funding source, model structure, and comparator selection appeared to co-occur descriptively. This observation should be interpreted cautiously because the review did not formally test associations between funding source and model design. These product-comparison frameworks also reflected different vaccine-valency assumptions, particularly bivalent cross-protection versus quadrivalent or nonavalent broader type coverage. Consistent with this descriptive pattern, mean CHEERS reporting completeness was somewhat lower among industry-funded than among non-industry records, as reported in the CHEERS reporting-completeness results; this difference should be read as a mapping observation rather than as evidence of bias.

### Contextual economic evidence records

The 11 contextual records were charted descriptively. They included three cost-consequence or cases-prevented analyses, one number-needed-to-vaccinate analysis, one fiscal net present value analysis, two simplified or hybrid cost-benefit analyses, one coverage-uptake impact analysis, one decision-tree risk-benefit analysis, one cost-of-delivery analysis, and one epidemiological transmission model without economic outcomes.

Among the contextual records, the cost-of-delivery analysis by Slavkovsky 2024 [51] addressed the post-WHO 2022 single-dose endorsement era, but it reported delivery-cost outcomes only, not health outcomes or formal ICERs. The Sargazi 2022 [52] record from Iran applied a CBA framework using discrete-choice willingness-to-pay elicitation and cost-of-illness data. The record from Lebanon applied a simplified cost-benefit ratio framework that excluded indirect productivity costs [53].

## Discussion

The findings show that the reported economic value of HPV vaccination is shaped by the interaction between vaccine characteristics, program design, model structure, and local decision context, rather than by vaccine efficacy alone. Across the 33 full comparative economic evaluations, cost-effectiveness estimates were driven by vaccine price, comparator choice, discounting, model structure, herd-immunity assumptions, threshold selection, and local screening context. These drivers did not operate independently. Vaccine price had to be interpreted in relation to the threshold used; herd-immunity assumptions were especially important when gender-neutral vaccination was evaluated; and productivity-cost inclusion mainly affected studies adopting societal, fiscal, or CBA perspectives.

The methodological evolution observed in this review was not a simple movement from static to dynamic modeling. Variation across evaluations was not driven only by individual economic inputs such as vaccine price or discount rate, but also by more structural modeling choices. Differences in transmission structure, the representation of HPV natural history, the calibration targets and validation approach used, and assumptions about the duration and mechanism of immune protection can each shift reported ICERs even when economic inputs are held constant. Recognizing these structural sources of variation matters because two models applied to the same country and vaccine price can still reach different conclusions. This is consistent with HPV comparative-modeling initiatives showing that harmonized input data do not necessarily produce harmonized projections when models differ in natural history structure, calibration targets, transmission assumptions, and validation procedures. Dynamic transmission models became more prominent for gender-neutral and 9-valent vaccination questions, where indirect protection and population-level effects are central. However, static cohort models remained common, particularly in LMIC settings where data availability and standardized LMIC tools shaped model choice, as reflected in the CERVIVAC evaluation from Iran and the PRIME-based evaluation from Türkiye [26,32]. The contextual records also show that HPV vaccination economic evidence has expanded beyond conventional ICER-based analysis into delivery-cost, fiscal, coverage, and risk-benefit questions. Importantly, reporting completeness, methodological credibility, and transferability did not necessarily align. A study may be carefully calibrated to UK screening practice or Japanese vaccination policy and still have limited relevance for settings with different vaccine prices, screening coverage, implementation capacity, or decision thresholds.

Previous reviews have generally concluded that HPV vaccination is often cost-effective, particularly when vaccine prices are low and the disease burden is high. Mahumud et al. (2020) systematically reviewed 9-valent HPV vaccine evaluations and concluded that the 9-valent vaccine was generally cost-effective compared with the quadrivalent vaccine under favorable price differentials [4]. Ng et al. (2018) synthesized studies of 9-valent, gender-neutral, and multiple-age-cohort vaccination and reported favorable cost-effectiveness under selected assumptions [5]. Segal and Rashid (2023) reviewed reviews of HPV economic evaluations and similarly emphasized generally favorable conclusions [15]. Ortega-Sanchez et al. (2008) reviewed early US HPV CEAs in the immediate post-licensure period [16].

Those reviews established the broad direction of the evidence. The present review adds a different layer by examining how those conclusions are produced. Rather than treating cost-effectiveness as a fixed property of HPV vaccination, this review shows that reported ICERs are outputs of modeling choices, comparator definitions, price assumptions, threshold selection, and local epidemiological and screening contexts.

The main contribution of this review is therefore methodological. It shows that reported cost-effectiveness depends not only on vaccine price and disease burden, but also on model structure, herd-immunity and cross-protection assumptions, discounting, threshold selection, and local screening context. It separates reporting completeness, assessed through CHEERS 2022, from methodological credibility, which was appraised across six health-economic domains. It also identifies recurring model families and product-comparison frameworks across the vaccine-technology timeline, while noting that funding source, model structure, and comparator choice tended to co-occur. Finally, the Welte-informed assessment separates a study’s relevance to its own decision setting from the question of whether its ICER can be transferred elsewhere [12]. This distinction is important because evidence supporting 9-valent or gender-neutral vaccination in one setting may not apply directly to another, especially where female vaccination coverage, vaccine prices, screening organization, and willingness-to-pay thresholds differ substantially.

CHEERS-based reporting completeness, methodological credibility, and transferability are conceptually distinct. CHEERS is a reporting checklist; it asks whether the information needed to interpret the study was reported [14]. Methodological credibility asks whether the model structure, comparator, calibration, herd-immunity treatment, uncertainty analysis, and threshold choice were appropriate for the decision problem. Transferability, including contextualization to the target setting, asks whether economic evaluation results can be meaningfully adapted across settings and depends on epidemiology, costs, clinical practice, resource use, perspective, and decision context [12]. These dimensions should not be collapsed into a single judgment of study quality. Some studies in this review achieved high reporting completeness while relying on assumptions or model structures that limited their usefulness for particular policy questions. Conversely, some records with only moderate reporting completeness used defensible approaches given local data constraints. For future reviews and policy appraisals, this distinction is essential.

Recent HPV-specific modeling guidance, including HPV-FRAME, emphasizes transparent reporting of model structure, natural history assumptions, calibration targets, vaccine protection assumptions, and policy context [61]. Although this review did not apply HPV-FRAME as a formal scoring instrument, the revised synthesis uses its underlying concerns to interpret model transparency, structural assumptions, reproducibility, and policy relevance across HPV vaccination economic evaluations.

The 11 contextual records broaden the map of HPV vaccination economic evidence beyond conventional CEA, CUA, and CBA. These records address policy questions that fall outside standard ICER-based evaluation, including program delivery costs, fiscal consequences, coverage uptake, risk-benefit framing, simplified or hybrid cost-benefit frameworks, and product-portfolio cost-consequence analyses. Their inclusion is appropriate for a scoping review because HPV vaccination decisions are not based only on ICERs. Decision makers also need evidence on affordability, delivery costs, implementation feasibility, fiscal sustainability, and uptake.

Specifically, the contextual records capture delivery costs [51], fiscal consequences [54], coverage uptake [55], risk-benefit framing [56], simplified or hybrid cost-benefit frameworks [52,53], and product-portfolio cost-consequence analyses [48–50]. These records should not be interpreted as full economic evaluations. Their value is that they identify policy questions not fully captured by conventional ICER-based studies, including fiscal sustainability under Gavi graduation, delivery-cost implications of single-dose schedules, and the economics of uptake-conditional vaccination.

The transferability of HPV vaccination economic evidence should also be interpreted in relation to demand-side and implementation factors. Economic evaluations often model vaccine uptake, coverage, and delivery costs as technical parameters, but these assumptions may be shaped by local beliefs, trust, and social norms. A previous scoping review of HPV vaccine acceptance in Islamic countries identified religious objections, concerns about fertility, perceived encouragement of sexual activity, and doubts about vaccine compatibility with religious beliefs as barriers to acceptance [13]. These findings are relevant for economic modeling because uptake assumptions are not purely technical; they depend on communication, trust, program design, and social context. ICERs from one setting may therefore transfer poorly to another unless local implementation conditions are considered.

Within the post-WHO 2022 single-dose endorsement era [8], formal QALY-based cost-effectiveness analyses of single-dose HPV vaccination have begun to emerge within the scope of this review. Two recent evaluations modeled single-dose schedules directly: Termrungruanglert 2024 [57] compared single-dose and two-dose bivalent, quadrivalent, and 9-valent vaccination in Thailand, and Gao et al. 2025 [47] evaluated the single-dose quadrivalent program adopted in Nigeria, both reporting favorable cost-effectiveness. The delivery-cost analysis by Slavkovsky 2024 complements these by addressing program delivery costs rather than health outcomes [51]. This emerging evidence remains limited but is growing rapidly, and single-dose schedules may change both delivery costs and program feasibility, particularly in LMICs.

A remaining biological uncertainty is the absence of a fully established correlate of long-term protection after HPV vaccination, particularly for single-dose schedules. This affects how models specify duration of protection, waning immunity, booster or revaccination requirements, and long-term prevention of HPV-associated cancers. Economic evaluations of single-dose schedules should therefore make these assumptions explicit and test them in scenario and sensitivity analyses.

Two recurring model families were observed. A head-to-head comparison framework, observed mainly in GSK-funded studies, used static Markov modeling with a “vaccine efficacy irrespective of HPV type” approach in product comparisons. A dynamic transmission lineage, observed mainly in Merck/MSD-funded studies, used the Elbasha-Dasbach-Insinga framework [6] for quadrivalent and 9-valent analyses across multiple jurisdictions. Funding source, model structure, and comparator selection appeared to co-occur descriptively. This review did not formally test for funding-related bias; such a question would require a quantitative framework controlling for model design, comparator choice, price assumptions, and outcome selection. The observation should therefore be interpreted as a mapping finding, not as evidence of bias. Nevertheless, it reinforces the need for transparent reporting of sponsor involvement, comparator selection, vaccine-price assumptions, efficacy assumptions, and cross-protection modeling in HPV vaccine economic evaluations [58–60].

### Limitations of the study

The findings should be interpreted in light of several limitations. First, gray literature, HTA reports, theses, conference abstracts, and editorials were excluded to maintain comparability across peer-reviewed economic evaluations. However, this may have excluded policy-relevant evidence used by national HTA agencies, immunization advisory bodies, and program decision makers. Only English-language publications were included, which may also have limited the representation of country-specific evaluations published in other languages. Second, the CHEERS 2022 assessment should be interpreted cautiously. The reviewer-defined CHEERS bands used in this review, high ≥80%, moderate 60–79%, and low < 60%, are not part of CHEERS 2022 and were applied only for descriptive comparison. CHEERS 2022 assesses reporting completeness, not methodological quality; therefore, the resulting percentages should be understood as structured indicators of reporting rather than validated quality scores. Third, the structured methodological credibility appraisal was descriptive and was not a validated risk-of-bias instrument. Although it used six predefined domains with explicit criteria, ratings still involved reviewer judgment. Similarly, categorical decisions about whether a record represented a full comparative economic evaluation or a contextual, partial, or non-standard economic record involved judgment, and other reviewers might have drawn some boundaries differently. Fourth, the recurring model-lineage analysis was descriptive and was not designed to test funding-related bias. The model-family mapping was based on published information on authorship, funding, model structure, comparator choice, and assumptions. It did not include citation-network analysis, author-network analysis, access to unpublished model documentation, or verification of confidential vaccine-price assumptions or sponsor involvement beyond what was disclosed. Finally, this review did not re-estimate ICERs, standardize costs to a common currency year, or attempt quantitative pooling. Such approaches were not appropriate because the included records differed substantially in perspective, model structure, outcome measure, comparator, time horizon, price assumptions, discounting, and threshold choice.

## Conclusion

This scoping review mapped 44 records of HPV vaccination economic evidence, including 33 full comparative economic evaluations and 11 contextual records. The evidence base has evolved from early static cohort modeling toward dynamic transmission, individual-based, and nonavalent policy models, although static standardized-tool models remain common in LMIC settings. Reported cost-effectiveness was shaped by vaccine price, discounting, herd immunity assumptions, cross-protection assumptions, threshold choice, comparator selection, and local screening context.

The review shows that HPV vaccination cost-effectiveness should not be interpreted as a fixed property of the vaccine alone. Instead, it reflects the interaction between assumptions, modeling choices, program design, and decision context within each evaluation. CHEERS reporting completeness varied and was distinct from methodological credibility. A study may be highly relevant to its own decision setting while still being difficult to apply elsewhere without reassessing model structure, vaccine price, comparator choice, screening context, discounting, thresholds, uncertainty analysis, and uptake assumptions.

Further formal economic evaluations of single-dose vaccination are needed, particularly studies addressing durability, waning assumptions, delivery feasibility, gender-neutral vaccination in LMICs, and country-specific analyses in underrepresented regions.

## Supporting information

S1 Table Study and intervention characteristics of included HPV vaccination economic evidence records. (DOCX)

S2 Table Model and economic evaluation characteristics of included records. (DOCX)

S3 Table Economic outcomes and drivers of variation in included records. (DOCX)

S4 Table Per-publication summary of contextualization and transferability. (DOCX)

S5 Table Item-level CHEERS 2022 reporting completeness matrix. (DOCX)

S6 Table Database-specific search strategies and administrative/search-filter criteria. (DOCX)
