# Supplementary material for: Methodological drivers, reporting completeness, and transferability in HPV vaccination economic evaluations: A scoping review
Source: PLoS One. 2026 Oct 14;21(10):e0360732. doi: 10.1371/journal.pone.0360732 (PMC13667800; doi:10.1371/journal.pone.0360732)
Supplement: Supplementary file 1 [file pone.0360732.s001.docx]

**S1 Table. Study and intervention characteristics of included HPV vaccination economic evidence records.**

| **First author, year, country** | **WHO region** | **Income group** | **Evidence category** | **Study type** | **Funding source** | **Industry involvement** | **Vaccine type** | **Target age** | **Sex/gender strategy** | **Primary comparator(s)** | **Screening context** | **Role in synthesis** |
| --- | --- | --- | --- | --- | --- | --- | --- | --- | --- | --- | --- | --- |
| Goldie et al., 2004, United States [3] | AMRO | High income | Full comparative economic evaluation | CEA, CUA | GlaxoSmithKline Biologicals + NIH (R01-CA93435) | Yes | 2v (HPV 16/18, hypothetical) | 12 | Girls only | No vaccination + no screening. No vaccination + screening only (multiple frequencies/ages). Various vaccine-screening combinations (80 strategies). | Organized | Primary synthesis |
| Elbasha et al., 2007, United States [6] | AMRO | High income | Full comparative economic evaluation | CEA, CUA, BIA | Merck Research Laboratories | Yes | 4v (HPV 6/11/16/18, Gardasil) | 12 (with 12–24 catch-up scenarios) | Multiple strategies compared: girls only, gender-neutral, with/without catch-up | No vaccination (current screening only). Five vaccination strategies: F12-only, F&M12, F12+CUF, F&M12+CUF, F&M12+CUF&M. | Organized | Primary synthesis |
| Boot et al., 2007, The Netherlands [34] | EURO | High income | Full comparative economic evaluation | CUA, HTA-style economic evaluation | RIVM (Dutch National Institute for Public Health and the Environment) | No | 2v + 4v compared (Cervarix and Gardasil) | 10–12 (preadolescent girls) | Girls only | No vaccination (current screening only) | Organized | Primary synthesis |
| French et al., 2007, Finland [60] | EURO | High income | Contextual economic evidence record | Epidemiological modeling only, not a full comparative economic evaluation. The publication explicitly notes that “cost-effectiveness has not really been considered.” | European Union Sixth Framework Program Network of Excellence (CCPRB, Contract LSHC-CT-2004-503465) | No | 2v (HPV 16 only, single-type model) | 12, 15, 18, 21 (compared) | Multiple strategies compared: girls only, gender-neutral | No vaccination (current screening only, 5-year intervals starting age 25) | Organized | Contextual mapping |
| Kulasingam et al., 2008, United Kingdom [35] | EURO | High income | Full comparative economic evaluation | CEA, CUA | Sanofi Pasteur MSD (SP-MSD); authors also report grants from Merck and CSL-Australia | Yes | 4v (HPV 6/11/16/18, Gardasil) | 12 | Girls only | No vaccination (current screening only, 3-year intervals from age 25–49 and 5-year intervals from age 50–64) | Organized | Primary synthesis |
| Coupé et al., 2009, The Netherlands [36] | EURO | High income | Full comparative economic evaluation | CEA, CUA | GlaxoSmithKline, acknowledged as a funder, stated to have no role in study design, data analysis or interpretation, or manuscript approval | Yes | 2v (HPV 16/18, Cervarix-equivalent assumed) | 12 | Girls only | Screening only (Dutch NHSCSP, 5-year intervals from age 30 to 60); also compared no screening + no vaccination, vaccination only, and screening + vaccination | Organized | Primary synthesis |
| Kim et al., 2009, United States [9] | AMRO | High income | Full comparative economic evaluation | CEA, CUA | National Cancer Institute (R01 CA93435); Centers for Disease Control and Prevention; American Cancer Society; Bill and Melinda Gates Foundation (30505) for related developing-country work | No | 2v / 4v (HPV 16/18, analysis applies to either Gardasil or Cervarix and models HPV 16/18 prevention only) | 35, 40, or 45 (older catch-up cohort focus, not pre-adolescents) | Women only | Multiple screening strategies without vaccination as comparators: cytology with HPV triage, annual, biennial, or triennial; combined cytology + HPV DNA testing, annual or biennial; HPV DNA testing with cytology triage; also, current screening practice weighted as 53% annual, 17% biennial, 11% triennial, and 15% every 5 years | Organized | Primary synthesis |
| Reynales-Shigematsu et al., 2009, Mexico [37] | AMRO | Upper middle income | Full comparative economic evaluation | CEA, CUA | CONACyT (Salud 2003-C01-038) and Merck Sharp & Dohme (MSD) grant; MSD stated to have no role in design, data, analysis, or writing | Yes | 4v (HPV 6/11/16/18, Gardasil) | 12 (varied 12–25 in sensitivity analysis) | Girls only | No intervention; conventional Pap with coverage every 3 years; conventional Pap every 5 years; vaccine alone; combinations | Organized (Mexican national program; 30% screening coverage assumed) | Primary synthesis |
| Dasbach et al., 2010, Hungary [38] | EURO | Upper middle income | Full comparative economic evaluation | CEA, CUA | Merck, North Wales, Pennsylvania, USA; EJD and EHE are Merck employees and own Merck stock; LN is an MSD Budapest employee and owns MSD stock; AB was a paid consultant for MSD Budapest | Yes | 4v (HPV 6/11/16/18, Gardasil) | 12 | Girls only | No vaccination + current screening; routine vaccination of 12-year-old girls; routine vaccination + 12–24-year catch-up, all combined with current Hungarian screening practices | Organized (Hungarian National Cancer Control Programme since 2003, but uptake was reportedly poor) | Primary synthesis |
| Demarteau and Standaert, 2010, France, Ireland, Italy [29] | EURO | High income | Full comparative economic evaluation | CEA, CUA | GlaxoSmithKline Biologicals. Both authors are employees of GSK Biologicals, Wavre, Belgium. | Yes | Hypothetical 2v vaccine, Vaccine A, HPV 16/18, versus hypothetical 4v vaccine, Vaccine B, HPV 6/11/16/18. Product comparison, not against no vaccination. | 12 | Girls only | Vaccine A versus Vaccine B head-to-head, bivalent product comparison versus quadrivalent product. Not against a no-vaccination scenario. | Country-specific organized programs: France, 60% screened every 3 years from age 25 to 65. Ireland, 40% screened every 3 years from age 25 to 44 plus selected ages. Italy, 64% screened every 3 years from age 25 to 60. | Primary synthesis |
| Capri et al., 2011, Italy [48] | EURO | High income | Contextual economic evidence record | Contextual economic evidence record, not a full comparative economic evaluation. Cost-consequence analysis, no ICER produced. | GlaxoSmithKline Biologicals SA, Belgium. Funding for study and medical writing services. ND is a GSK employee. | Yes | Hypothetical 2v, Cervarix HPV 16/18, versus hypothetical 4v, Gardasil HPV 6/11/16/18. Product comparison. | NR, focuses on the steady-state population effect | Girls only | Bivalent versus quadrivalent head-to-head product comparison. | Italian regional Pap smear screening, with incomplete coverage. Only 1.6 million of 5.6 million eligible women attended, according to the 2008 PASSI survey. | Contextual mapping |
| Praditsitthikorn et al., 2011, Thailand [21] | SEARO | Lower middle income | Full comparative economic evaluation | CUA | World Bank Population and Reproductive Health Capacity Building Program. HITAP was supported by the Thai Health Promotion Foundation, the Health Systems Research Institute, the Bureau of Policy and Strategy, and the Thai Health Global Link Initiative. | No | 2v / 4v, HPV 16/18 vaccine. Both Gardasil and Cervarix were licensed in Thailand. | 15 to 60 years, with multiple ages tested annually from 15 to 60 years | Girls and women only | Multiple comparators, including no intervention, Pap smear every 5 or 10 years, VIA every 5 or 10 years, VIA plus sequential Pap smear, HPV vaccination at ages 15 to 60, and combinations of vaccination and screening. | Organized. The Thai Ministry of Public Health Pap smears were available in all health centers, but only 9% target coverage was achieved. VIA was available in 15 of 76 provinces, with 19% target coverage. | Primary synthesis |
| Schobert et al., 2012, Germany [27] | EURO | High income | Full comparative economic evaluation | CEA, CUA | Sanofi Pasteur MSD (unrestricted grant to Friedrich-Alexander-University. Medical writing also funded by SP-MSD) | Yes | 4v (HPV 6/11/16/18, Gardasil) | 12–17 (per German national recommendation since 2007) | Girls only | No vaccination (current screening only, annual cytology from age 20) | Organized | Primary synthesis |
| Termrungruanglert et al., 2012, Thailand [39] | SEARO | Lower middle income | Full comparative economic evaluation | CEA, CUA | MSD Thailand, Merck Sharp & Dohme. The views stated were not endorsed by the sponsor. | Yes | 4v, HPV 6/11/16/18, Gardasil | 12 | Girls only | Current management practice in Thailand involves treatment only at baseline, with no comparison with VIA or Pap-only strategies. | Implicit, current Thai practice baseline. | Primary synthesis |
| Aponte-González et al., 2013, Colombia [22] | AMRO | Upper middle income | Full comparative economic evaluation | CEA, CUA | NR, academic study. No industry funding was declared. Ethics committee approval from Universidad Nacional de Colombia. | No | Hypothetical 2v versus hypothetical 4v versus no vaccination, three-strategy comparison. | 12 | Girls only | Three-arm comparison: no vaccination plus screening alone, current Colombian practice with 70% Pap coverage and 1-1-3 programs. Bivalent vaccination plus screening. Quadrivalent vaccination plus screening. | Organized. The Colombian National Program for Control and Early Detection of Cervical Cancer since 1991. 1-1-3 programs: after two negative Pap tests, 3-year intervals. | Primary synthesis |
| Berkhof et al., 2013, CEECA region, including Slovenia, Poland, Georgia [40] | EURO | Mixed | Full comparative economic evaluation | CEA, CUA | European Commission 7th Framework PREHDICT, HEALTH F3 2010 242061. Netherlands Organisation for Health Research and Development, ZonMW 121030032. Instituto de Salud Carlos III, Spain. Agència de Gestió d'Ajuts Universitaris i de Recerca, Catalonia. | No, primary funding was public or academic. One author disclosed speaker fees from Qiagen and incidental consultancy from Sanofi Pasteur and GSK. The ICO Unit was involved in HPV vaccine trials sponsored by GSK, Merck, and Sanofi. | 2v, HPV 16/18, assumed to prevent 70% of all cervical cancer cases | 12 | Girls only | CEECA 28 country threshold analysis: vaccination versus no vaccination. Detailed 3-country analysis: cancer treatment only, screening alone using Pap or HPV DNA at 3, 6, or 10-year intervals, HPV 16/18 vaccination alone, and combined vaccination plus screening at 10 or 6-year intervals. | Mixed. Slovenia had recently established organized screening. Poland had organized screening. Georgia had opportunistic screening. The wider CEECA region was largely opportunistic. | Primary synthesis |
| Bresse et al., 2013, France [50] | EURO | High income | Contextual economic evidence record | Contextual economic evidence record, not a full comparative economic evaluation. Cost-consequence analysis, no ICER produced. | SPMSD, Sanofi Pasteur MSD. Editorial assistance from Ossian Health Economics and Communications. | Yes. XB, MA, and NL were employees of SPMSD. SR and RM were employees of HEVA, which received consulting fees from SPMSD. The study was supported by SPMSD funding. | 2v versus 4v, HPV 16/18 versus HPV 6/11/16/18, both compared with screening alone baseline | 14 | Girls only | Three strategies: screening only as a baseline, bivalent vaccine plus screening, and quadrivalent vaccine plus screening, compared via a common baseline. | Organized triennial cytology screening for women aged 25 to 65 according to the French HAS recommendation. Approximately 55% regular cytology coverage. Approximately 70% of new cervical cancer cases occurred in unscreened women. | Contextual mapping |
| Kim et al., 2013a, the sub-Saharan Africa region, Uganda and South Africa [23] | AFRO | Mixed | Full comparative economic evaluation | CEA, CUA | Bill & Melinda Gates Foundation, grant 30505. Mireia Diaz and UNIC ICO received partial support from the Instituto de Salud Carlos III, the Spanish government, and the Agència de Gestió d'Ajuts Universitaris i de Recerca, Catalonia. | No. Philanthropic and public funding only. Authors disclosed no potential conflicts of interest. | 2v, HPV 16/18, assumes 70% cervical cancer prevention. Both bivalent and quadrivalent vaccines were licensed in some countries but not differentiated in this analysis. | 12, pre-adolescent, and before sexual debut | Girls only | 48 country analysis: vaccination versus no vaccination. Uganda and South Africa analysis: no intervention; VIA once at age 35; cytology three times in a lifetime; HPV DNA testing three times in a lifetime using two visits; HPV vaccination alone; and HPV vaccination plus HPV DNA testing three times in a lifetime. | Limited to demonstration or opportunistic screening in most sub-Saharan African settings. South Africa had a national cytology program with 70% target coverage. Uganda and others were largely opportunistic. Rwanda launched a national HPV DNA program in 2011. | Primary synthesis |
| Kim et al., 2013b, EMENA region, including Algeria, Lebanon, Turkey [24] | AFRO, EMRO, EURO | Mixed | Full comparative economic evaluation | CEA, CUA | Bill & Melinda Gates Foundation, grant 30505. Mireia Diaz and UNIC ICO received partial support from the Instituto de Salud Carlos III, the Spanish government, and the Agència de Gestió d'Ajuts Universitaris i de Recerca, Catalonia. | No, primary funding was philanthropic and public. One author, M. Seoud, disclosed honoraria, research grants, and travel support from MSD and GlaxoSmithKline. The first six authors disclosed no conflicts. | 2v, HPV 16/18, assumes 100% efficacy and 70% HPV 16/18 cervical cancer attribution. Bivalent and quadrivalent vaccines were licensed in 13 of 20 EMENA countries but not differentiated. | 12, pre-adolescent, and before sexual debut | Girls only | 20 country analysis: vaccination versus no vaccination. Algeria, Lebanon, and Turkey analysis: no intervention, cytology screening alone using 1, 3, or 5 year intervals reflecting national practice, HPV DNA testing alone in sensitivity scenario, HPV vaccination alone, and HPV vaccination plus cytology screening. | Mostly opportunistic in EMENA, with few organized population-based programs. Turkey had a 5-yearly cytology from age 35 to 65 according to national guidelines. Algeria and Lebanon had variable cytology recommendations. | Primary synthesis |
| Jit et al., 2014, England and Canada [7] | EURO, AMRO | High income | Contextual economic evidence record | Contextual economic evidence record, not a full comparative economic evaluation. Clinical impact analysis using number-needed-to-vaccinate, no ICER. | Canadian Institutes of Health Research, CRN 83320. Canada Research Chairs program. Québec Ministry of Health. UK Public Health England institutional support. | No, primary funding was non-industry. Brisson disclosed consulting and travel reimbursement from Merck Frosst and GlaxoSmithKline. Other authors reported no conflicts. | 2v plus cross-protection, HPV 16/18 plus cross protection against HPV 31/33/45/52/58. Both bivalent and quadrivalent vaccines were considered equivalent in this analysis. | 12 routine, with a catch-up to 18 in the first year | Girls only | Three-dose versus two-dose schedules. The three-dose schedule assumed lifetime protection. Two-dose protection was explored at 30, 20, 10, and 15 years. One scenario assumed lifetime two-dose protection but no cross-protection. | Organized screening in England through the UK NHS and in Canada through provincial programs. Current screening behavior was assumed unchanged with vaccination. | Contextual mapping |
| Bonanni et al., 2015, Italy [49] | EURO | High income | Contextual economic evidence record | Contextual economic evidence record, not a full comparative economic evaluation. Population impact analysis using cases prevented, no ICER. | GlaxoSmithKline Biologicals SA funded the study and was involved in all stages of study conduct, including analysis, and covered all development and publication costs. | Yes. GSK funded. ND was a GSK employee with stock or options. PB, SB, GG, and GLT received grants or honoraria from Pfizer, GSK, Sanofi Pasteur MSD, Novartis Vaccines, and Crucell. PB and SB served on advisory boards. GLT was the project manager of an HTA report on Cervarix and Gardasil. | 2v, HPV 16/18 AS04 adjuvanted, Cervarix, explicit GSK product focus | 11, primary target in the Italian National Vaccination Plan | Girls primary, with comparison including boys vaccination | Multiple strategies: screening only, 11-year-old girls vaccination, 15-year-old female catch-up plus 11-year-old girls vaccination, 25-year-old female catch-up plus 11-year-old girls vaccination, and 11-year-old boys vaccination plus 11-year-old girls vaccination. | The Italian organized triennial cytology screening for ages 25 to 64. Screening coverage assumed at 65%, with 35% never screened. | Contextual mapping |
| Aljunid et al., 2016, Malaysia [41] | WPRO | Upper middle income | Full comparative economic evaluation | CEA, CUA | GlaxoSmithKline Pty Ltd, acknowledged funder. Universiti Kebangsaan Malaysia ethics approval, FF 370 2012. MOH Malaysia approval, NMRR 12 1207 14109. | Yes, GSK funded. The study explicitly acknowledged funding from GlaxoSmithKline Pty Ltd. The authors declared no competing interests, but the funding source was explicit. | 2v, HPV 16/18 AS04 adjuvanted, Cervarix. Implicit GSK product focus given by the funder. | 13, Malaysian school-based program age | Girls only | Three strategies: no vaccination as an added scenario, a 3-dose schedule as the current Malaysian practice with an RM134 per-dose administration cost, and a 2-dose schedule with price parity assumed. | Implicit. No explicit screening modeling, but screening results were referenced in the model. | Primary synthesis |
| Setiawan et al., 2016a, Indonesia [42] | SEARO | Lower middle income | Full comparative economic evaluation | CUA | Directorate General of Higher Education, DIKTI Scholarship, Ministry of National Education, Indonesia, Setiawan PhD scholarship. The funder had no role in study design, analysis, writing, or the decision to submit. | No. DIKTI scholarship from the Indonesian government. The authors indicated no conflicts of interest related to the article content. | 2v, HPV 16/18. Efficacy data primarily from PATRICIA and Paavonen 2009 bivalent trial. The model did not differentiate between bivalent and quadrivalent vaccines in the base case, acknowledged as a limitation. | 12, before sexual debut | Girls only | Three strategies: no intervention as reference, VIA screening alone every 3 years from age 30 to 60 with 63.6% coverage, and VIA screening plus HPV vaccination of 12-year-old girls. | The Indonesian Ministry of Health VIA screening program was launched in 2007. Screening every 3 years from age 30 to 60. Recommended 63.6% coverage. Cryotherapy adherence 83.1%. Detection rate 69.4%, based on the 2012 Indonesian VIA single-visit study. | Primary synthesis |
| Setiawan et al., 2016b, Indonesia [54] | SEARO | Lower middle income | Contextual economic evidence record | Contextual economic evidence record, not a full comparative economic evaluation. Fiscal NPV analysis from a government perspective. | Setiawan PhD scholarship from the Directorate General of Higher Education, DIKTI, Ministry of National Education, Indonesia. Global Market Access Solutions, GMAS, provided salaries for NK and MPC. The funder had no role in study design, data collection, analysis, decision to publish, or preparation. | Mixed. DIKTI, the Indonesian government, supported the primary author, and GMAS commercial consultancy supports NK and MPC. JCW reported personal fees from GSK and was co-founder or stakeholder of ViciniVax BV. MJP reported grants and personal fees from GSK and SPSMD relevant to the topic, additional unrelated industry relationships, and 3% stock in Ingress Health. | Same as Setiawan 2016a, bivalent HPV 16/18 with PATRICIA derived efficacy. | 12, before sexual debut | Girls only | Three strategies: cervical screening alone, HPV vaccination alone, and HPV vaccination plus cervical screening, each compared with no intervention as a reference. | VIA screening every 3 years from age 30 to 60. Yearly coverage 21.2%, equal to 63.6% over 3 years. | Contextual mapping |
| Vargas Parada and Veerman, 2016, Chile [33] | AMRO | Upper middle income | Full comparative economic evaluation | CEA, DALY-based, per DALY averted ICER | No funding required. Result of a thesis submitted for the Master's in Health and Economics degree at the University of Queensland, Australia. | No. Academic master's thesis output. The authors indicated no conflicts of interest related to the article content. | 4v, quadrivalent Gardasil. HPV 6/11/16/18 efficacy from Palefsky 2011 NEJM RCT MSM substudy. | 30, HIV positive men aged 25 to 34 years | Men only, HIV positive population, sex specific evaluation | Two strategies: 3-dose quadrivalent HPV vaccination for HIV positive men aged 25 to 34 years, and no systematic HPV prevention as the current Chilean practice. | NR. No anal cancer screening modeled and no AIN screening. Chile has no anal cancer screening program. AIN diagnosis and treatment were also not modeled. | Primary synthesis |
| Kosen et al., 2017, Indonesia [43] | SEARO | Lower middle income | Full comparative economic evaluation | CEA, CUA | Merck & Co., Inc. Funding statement reported that the views expressed were those of the authors and approved by Merck & Co., Inc. Acknowledgments credited Merck personnel Amit Sharad Kulkarni, Matthew Pillsbury, Andrew Pavelyev, and Anuj Walia for data analysis and collection. | Yes. Merck-funded, with explicit sponsor approval. The conflict-of-interest statement said none was declared, but Merck's funding role and Merck personnel acknowledgments demonstrate industry involvement. The methodological framework was based on the original Merck quadrivalent model by Elbasha 2007. | 4v, quadrivalent HPV 6/11/16/18, Gardasil, explicit Merck product. | 11 to 12 primary target. 12 to 26 catch-up scenarios. | Girls primary, female catch-up only | Three strategies: screening only with Pap smear, as the current Indonesian practice; 45% follow-up after abnormal result; and 2-dose quadrivalent HPV vaccination of 11- to 12-year-old girls without catch-up, and 2-dose quadrivalent HPV vaccination of 11- to 12-year-old girls plus female catch-up from 12 to 26 years. | Indonesian Pap smear screening. 45% follow-up rate after an abnormal Pap result. Indonesian National Cervical Cancer Registry 2010 to 2012 data. | Primary synthesis |
| Mennini et al., 2017, Italy [44] | EURO | High income | Full comparative economic evaluation | CEA, CUA | Sanofi Pasteur MSD, SPMSD. The study was sponsored by Sanofi Pasteur MSD. Mathieu Uhart, Alessandro Rinaldi, and Natalie Largeron were affiliated with Sanofi Pasteur MSD as the funding organization and had roles in study design, data collection, analysis, interpretation, and manuscript writing. | Yes. Sanofi Pasteur MSD funded. Extensive conflicts disclosed. Mathieu Uhart, Alessandro Rinaldi, and Nathalie Largeron were SPMSD employees. Giacomo Plazzotta and Florence Bianic were Mapi Group employees who received SPMSD funding. Francesco Saverio Mennini, Paolo Bonanni, Chiara de Waure, and Gianluca Baio received grants from SPMSD for advisory boards, expert meetings, and speaking at congresses. | 9v, Gardasil 9, HPV 6/11/16/18/31/33/45/52/58, SPMSD product. Also, 4v Gardasil and 2v Cervarix as comparators. | 12, Italian primary target since 2007 | Girls primary, plus girls and boys universal scenarios | Six strategies: screening only, cervical screening plus bivalent vaccine girls only, cervical screening plus quadrivalent vaccine girls only, cervical screening plus quadrivalent vaccine girls and boys, cervical screening plus 9-valent vaccine girls only, and cervical screening plus 9-valent vaccine girls and boys. | Organized Pap smear-based screening every 3 years for women aged 25 to 64 years; 77% screened at least once every 3 years. Primary HPV testing for women aged 30 years or older had been introduced in some regions, but data were limited. | Primary synthesis |
| Chesson et al., 2018, United States [28] | AMRO | High income | Full comparative economic evaluation | CEA, CUA | US Centers for Disease Control and Prevention, CDC, institutional funding. Authors were CDC employees from the Division of STD Prevention, Division of Viral Diseases, and Division of Cancer Prevention and Control. Disclaimer stated that findings and conclusions did not necessarily represent the official CDC position. | No. US CDC institutional, non-industry. The authors reported no conflicts of interest. | 9v, Gardasil 9, was the only HPV vaccine available in the United States since 2015, after the 4vHPV phase out. | 11 to 12, US ACIP routine target. Vaccination can start at age 9. | Both girls and boys | Two scenarios: comparison scenario with 9vHPV for females aged 12 to 26 and males aged 12 to 21, current ACIP. Expanded scenario with 9vHPV for females and males aged 12 to 26, harmonized age cap. Both also compared with no vaccination as an additional reference. | Implicit. Cervical cancer screening was incorporated indirectly through observed CIN and cervical cancer rates from the 2006 to 2010 era data. Rates were assumed constant over a 100-year horizon in the absence of HPV vaccination. | Primary synthesis |
| Yaghoubi et al., 2018, Iran [32] | EMRO | Lower middle income | Full comparative economic evaluation | CEA, DALY-based, per DALY averted ICER | World Health Organization, grant no. 2015/516673-0. WHO Regional Office for Eastern Mediterranean, EMRO, co-author Chaudhri. | No. WHO funded, international agency. The authors declared no competing interests. | 4v, quadrivalent Gardasil. Cervarix bivalent was not considered because it was not approved by the Food and Drug Administration of Iran. | 9, based on the Iranian Center for Disease Control and Management expert opinion | Girls only | Two strategies: no vaccination, current Iranian status, and 3-dose quadrivalent HPV vaccination of 9-year-old girls. Scenario analysis included a 2-dose alternative. | NR. No cervical cancer screening modeled because of the CERVIVAC model limitation. Current Iranian screening practices were implicit in incidence rates. | Primary synthesis |
| Bahr et al., 2019, Lebanon [53] | EMRO | Lower middle income | Contextual economic evidence record | Contextual economic evidence record, not a full comparative economic evaluation. Simplified cost-benefit ratio, no productivity costs. | Funding: none declared. Department of Epidemiology and Population Health, Faculty of Health Sciences, American University of Beirut, institutional. Institutional Review Board clearance from the American University of Beirut. | No. Academic institutional analysis with no funding source. Competing interests: none declared. | 2v, Cervarix bivalent, explicitly chosen as the cheapest. Gardasil quadrivalent and 9-valent vaccines were also licensed by the Lebanese Ministry of Public Health but were not cost-considered. | 11, Lebanese Ministry of Public Health consideration | Girls only | Two strategies: no vaccination, the current Lebanese practice, and Cervarix only HPV vaccination of 11-year-old girls, the cheapest option. Other vaccines were not considered economically viable. | Pap smear screening at primary health care centers, US$10 per test. This was recommended as an alternative in conclusion but was not modeled as a comparator. | Contextual mapping |
| Datta et al., 2019, United Kingdom [10] | EURO | High income | Full comparative economic evaluation | CEA, CUA, QALY-based, threshold dose price methodology | UK Department of Health, Grant 027/0089. The funder had no role in study design, data collection, analysis and interpretation, manuscript writing, or the decision to submit. | No. UK Department of Health-funded, non-industry. The authors declared no competing interests. One author, MU, was a shareholder in Clinvivo Ltd and reported minor NIHR research/editor fees. | Three vaccines evaluated: 2v Cervarix bivalent, used in the UK from 2008 to 2012; 4v Gardasil quadrivalent, used in the UK from 2012 to 2018; and 9v Gardasil 9 nonavalent, adopted in the UK in 2019. | 12 to 13, UK school-based program | Both girls and boys, with a gender-neutral analysis central to the study | Six vaccination strategies: halted vaccination, historical 2008 to 2016 plus halt from 2017 onwards; girls only, historical plus 85% from 2017; girls plus boys, historical plus 85% both from 2017; girls plus boys equal, 42.5% each from 2017; girls naïve, no historical vaccination plus 60% girls from 2008; and girls plus boys naïve, no historical vaccination plus 60% both from 2008. | NR. Cervical cancer screening was explicitly not modeled. It was assumed to continue in its current form, and this was acknowledged as a limitation. | Primary synthesis |
| Lee et al., 2019, South Korea [30] | WPRO | High income | Full comparative economic evaluation | CEA, CUA, QALY, and LY-based, full ICER reporting at multiple discount rates | GlaxoSmithKline Biologicals SA. The funding source was involved in all stages of study conduct and analysis and funded all costs associated with manuscript development and publication. | Yes. GSK funded, Cervarix manufacturer. Multiple GSK employee co-authors: Jang, Sohn, Van Kriekinge, and Lee IH, who was at GSK at the time of the study and later at Gilead Sciences. Korean academic co-authors reported personal fees from GSK during and outside the study. | Two vaccines compared head to head: 2v AS04 HPV 16/18v, Cervarix GSK, versus 4v HPV 6/11/16/18v, Gardasil Merck. Both used a 2-dose schedule. | 12, the Korean national program target was launched in June 2016 | Girls only | Three strategies: screening alone, current Korean Pap smear every 2 years from age 30 to 74 with 49.5% coverage; screening plus 2-dose AS04 HPV 16/18v, Cervarix; and screening plus 2-dose 4vHPVv, Gardasil. | Pap smear testing every 2 years from age 30 to 74 under the current Korean government funding, with 49.5% coverage. | Primary synthesis |
| Messoudi et al., 2019, Morocco [45] | EMRO | Lower middle income | Full comparative economic evaluation | CEA, life years saved based, not QALY or DALY based | Spanish public, EU, Catalan, and Moroccan foundation funding: Instituto de Salud Carlos III, ISCIII RD12/0036/0056, PI11/02090, PIE16/00049, PI16/01254, co funded by ERDF; Catalan Generalitat SGR 2014SGR756, 2014SGR1077, 2017SGR1718; EU FP7 COHEAHR Health F3 2013 603019; FP7 People 2013 IRSES612216 MNSIRSES; RecerCaixa 2015ACUP00129; CERCA Programme/Generalitat de Catalunya institutional support; and Lalla Salma Foundation for Cancer Prevention and Treatment, Morocco, financial and technical support. | No. Spanish public, EU, Catalan, and Moroccan foundation funding. WM, TE, CN, NT, AZ, GA, DM, and MD declared no competing interests. Diaz M disclosed institutional support: HPV vaccine trials and epidemiological studies at ICO Barcelona were sponsored by GSK, Merck, Roche, and Sanofi Pasteur MSD, but this was not personal support. | 2v, bivalent HPV 16/18, Cervarix equivalent. Quadrivalent and 9-valent vaccines were not modeled. | 14, Moroccan pre-adolescent target | Girls only | Multiple strategies: no intervention; VIA screening alone at 3-year, 5-year, 10-year, 20-year, or once in a lifetime frequency at 10% coverage; HPV bivalent vaccination alone for 14-year-old girls at 70% coverage; and combined VIA every 3 years plus HPV vaccination. | Current Moroccan VIA program: opportunistic VIA every 3 years for women aged 30 to 49, launched in 2010. National coverage was approximately 6% to 10%. Around 80% to 90% of cervical cancer cases were diagnosed at an advanced stage. Sensitivity analyses varied screening frequency, coverage, sensitivity, and cost. | Primary synthesis |
| Smith et al., 2019, Canada [55] | AMRO | High income | Contextual economic evidence record | Contextual economic evidence record, not a full comparative economic evaluation. Coverage-uptake impact analysis, no ICER. | Canadian Partnership Against Cancer, institutional employer of all authors. OncoSim model led by Canadian Partnership Against Cancer. Model development by Statistics Canada through Health Canada funding. The Partnership endorsed the Union for International Cancer Control global pledge to eliminate cervical cancer. | No. Canadian Partnership Against Cancer institutional funding, Statistics Canada, and Health Canada model development funding. Authors declared no conflicts of interest. | 4v, quadrivalent vaccine assumed throughout. 9-valent transition was acknowledged as expected to have a modest effect, but no separate 9-valent modeling. | 9 to 14, varied by jurisdiction, including Saskatchewan grade 6 or age 13, Ontario grade 7 to 8, and Alberta grade 5 to 6 transition. | Girls only as study focus. Boys' inclusion in school-based programs was noted but not separately modeled. | Three strategies via uptake variation: 0% HPV vaccination as the no-intervention baseline; 67% HPV vaccination, the current Canadian weighted average, ranging from 55% in the Northwest Territories to 92% in Newfoundland; and 90% HPV vaccination, the Government of Canada 2025 target. | Canadian triennial Pap test for women aged 21 to 69, with 72% national participation, 60% sensitivity for CIN2+, and 97% specificity. Current Canadian standard practice. | Contextual mapping |
| Tang et al., 2019, Taiwan [31] | WPRO | High income | Full comparative economic evaluation | CEA, CUA, QALY, and LY-based, full ICER reporting with probabilistic sensitivity analysis. | GlaxoSmithKline Biologicals SA, GSK study identifier HO 16 17794. The funding source was involved in all stages of study conduct and analysis and covered all costs associated with manuscript development and publication. | Yes. GSK funded, Cervarix manufacturer. GSK employee coauthors included Hsieh JY, Mukherjee P, Van Kriekinge G, and Lee C. Taiwanese academic coauthors reported no disclosures, but GSK funding was explicitly stated. | Four vaccines compared head-to-head: 2v AS04 HPV 16/18v, Cervarix GSK, 4v HPV 6/11/16/18v, Gardasil Merck, and 9v HPV 6/11/16/18/31/33/45/52/58v, Gardasil 9 Merck, using both 2-dose and 3-dose schedules. | 12, Taiwanese 7th grade target | Girls only | Five strategies: screening alone, current Taiwanese annual Pap smear with 27.6% coverage in 2014, screening plus 2-dose AS04 HPV 16/18v, screening plus 2-dose 4vHPVv, screening plus 2-dose 9vHPVv, and screening plus 3-dose 9vHPVv. | The Taiwanese national annual Pap smear program since 1995. Coverage was 27.4% in 2007 and 27.6% in 2014. Incidence and mortality rates decreased by 46% and 47% in 2006 to 2007, attributed to the program. | Primary synthesis |
| Chauhan et al., 2020, India [46] | SEARO | Lower middle income | Full comparative economic evaluation | CEA, CUA, QALY-based, full ICER reporting with Monte Carlo probabilistic sensitivity analysis. | Department of Health Research, Ministry of Health and Family Welfare, Government of India, grant T 11011/02/2017 HR. The funders had no role in study design, data collection and analysis, decision to publish, or manuscript preparation. | No. Indian Department of Health Research government grant, non-industry. Authors declared no competing interests. | 2v, bivalent HPV 16/18 strain coverage, based on Indian HPV vaccination experience. | 11, Indian pre-adolescent target | Girls only | Multiple strategies: no screening plus no vaccination as baseline, VIA at 3-, 5-, or 10-year intervals, Pap smear at 3, 5, or 10 year intervals, HPV DNA testing at 3, 5, or 10 year intervals, HPV vaccination alone for 11-year-old girls, and HPV vaccination plus screening with VIA at 5 or 10 year intervals. | The current Indian NPCDCS program used VIA every 5 years for women aged 30 to 65 in a 100-district pilot. Multi-frequency comparison was central to the research question. Auxiliary nurse midwife screening at sub-centers followed NPCDCS guidelines. | Primary synthesis |
| Kitano, 2022, Japan [56] | WPRO | High income | Contextual economic evidence record | Risk-benefit analysis, QALY-based, with ICER reported as secondary. The primary outcome was the risk-benefit ratio. | No funding declared, single-author study. | No. Single author, no funding declared. Conflict of interest: none to declare. | 9v, Gardasil 9. At the time of the study, the 9-valent vaccine was approved only for women in Japan, while the quadrivalent vaccine was approved for males aged 9 years or older. | 12, the Japanese national immunization program age for girls | Boys only, male only analysis, rare in HPV CEA literature | Two strategies: HPV vaccination of a 12-year-old Japanese boy with a 9-valent series, and no vaccination. The risk-benefit framework compares the benefits of preventing five HPV-related diseases with the risks of acute and chronic adverse events. | NR. No cervical cancer screening was modeled because the analysis was male-only. Japanese opportunistic screening was implicit. | Contextual mapping |
| Owusu-Edusei et al., 2022, United Kingdom [25] | EURO | High income | Full comparative economic evaluation | CEA, CUA, QALY-based, full ICER reporting with comprehensive one-way sensitivity analysis and threshold analysis. | Merck Sharp & Dohme Corp, a subsidiary of Merck & Co., Inc., Kenilworth, New Jersey. The study was performed by Merck Sharp & Dohme Corp. | Yes. Merck funded, Gardasil and Gardasil 9 manufacturer. Four of five authors were Merck Sharp & Dohme employees. Authors may hold Merck stock or stock options. Favato G was the only academic coauthor and conceptualized the study with the Merck team. | 9v, Gardasil 9, versus 4v, Gardasil. The 9-valent vaccine covered HPV types 6, 11, 16, 18, 31, 33, 45, 52, and 58. The 4-valent vaccine covered HPV types 6, 11, 16, and 18. | 12 to 13, UK Year 8 school-based program target | Both girls and boys, gender neutral, with boys eligible from the 2019 to 2020 UK school year, according to JCVI 2018 | Two strategies: universal vaccination with the 4-valent HPV vaccine, UV4V, and universal vaccination with the 9-valent HPV vaccine, UV9V. Both scenarios included the impact of the historical UK vaccination program from 2008 to 2019. | The UK national cervical screening program is implicit. The model assumed no change to other screening programs or vaccine uptake. UK Public Health England and NHS framework. | Primary synthesis |
| Sargazi et al., 2022, Iran | EMRO | Lower middle income | Contextual economic evidence record | Contextual economic evidence record, not a full comparative economic evaluation. Hybrid cost-benefit analysis with WTP discrete-choice experiment and cost-of-illness data. | Tehran University of Medical Sciences, TUMS. MSc thesis in health economics. Registration code IR.TUMS.SPH.REC.1398.121. Acknowledgment to TUMS School of Public Health funding. | No. TUMS academic non-industry funding through MSc thesis support. The authors declared no conflict of interest. | 2v, bivalent HPV 16/18, Cervarix equivalent, and 4v, quadrivalent HPV 6/11/16/18, Gardasil equivalent. | 9 to 14, the WHO-recommended age range according to the WHO 2016 guide to introducing the HPV vaccine | Girls only, WHO's first priority recommendation | Three strategies: no vaccination in the current Iranian context, where HPV vaccination was not in the immunization schedule, bivalent HPV vaccination, and quadrivalent HPV vaccination. Both vaccines were compared with no vaccination. | Cervical cancer screening was not organized in Iran, according to the Ghahramani 2020 systematic review cited in the study. | Contextual mapping |
| Palmer et al., 2023, Japan [11] | WPRO | High income | Full comparative economic evaluation | CEA, CUA, QALY-based, full ICER reporting with one-way sensitivity analysis and scenario analysis. | Merck Sharp & Dohme LLC, a subsidiary of Merck & Co., Inc., Rahway, NJ, USA. The manuscript was funded by Merck Sharp & Dohme LLC. | Yes. Merck funded the manufacturers of Gardasil 4v and Gardasil 9. All six authors were Merck or MSD employees and may hold equity interests in Merck & Co., Inc. | 9v, Gardasil 9, for girls aged 12 to 16 and post-March 2023 catch-up girls aged 17 to 26. 4v, Gardasil, for boys aged 12 to 16 because 9vHPV was not yet approved for boys in Japan at the time of the study. The post-April 2023 transition to 9vHPV in the national immunization program was acknowledged. | 12 to 16 years old, Japanese National Immunization Program primary cohort. 17 to 26, catch-up cohort from April 2022 to March 2025. | Both a gender-neutral strategy, including boys aged 12 to 16, girls aged 12 to 16, and women aged 17 to 26 in the catch-up cohort. | Two strategies: female-only vaccination with 9vHPV for primary girls and female catch-up vaccination, and gender-neutral vaccination with 9vHPV for girls and 4vHPV for boys. Both scenarios included the April 2022 reinstatement of the governmental recommendation and the transition from 4vHPV to 9vHPV for girls from April 2023 onward. | Japanese current cervical cancer screening included, using 2022 National Health Insurance fee schedule cost data and Shirobon Net 2022 Medical Fee Score Table. | Primary synthesis |
| Slavkovsky et al., 2024, Ethiopia, Guyana, Rwanda, Sri Lanka, and Uganda | AFRO, AMRO, SEARO | Mixed | Contextual economic evidence record | Contextual economic evidence record, not a full comparative economic evaluation. Cost-of-delivery analysis with no health outcomes and no ICER. | Bill & Melinda Gates Foundation, INV 005053 and INV 552340. PATH research organization. Monograph sponsored by Bill & Melinda Gates Foundation, State of the Science of Single Dose Prophylactic HPV Vaccination. The funder had no role in study design, data collection and analysis, decision to publish, or manuscript preparation. | No. Bill & Melinda Gates Foundation non-industry funding. The authors declared no conflict of interest. | HPV vaccine delivery schedule comparison; vaccine product type not separately specified. | 9 to 13, WHO recommended the primary target population | Girls only, WHO's primary target population | Two strategies: a 2-dose schedule based on the 2019 implementation in the five countries, and a hypothetical single-dose schedule based on the WHO 2022 endorsement, both using the same operational context modeling. | NR. Cervical cancer screening was not modeled because the analysis focused only on vaccine program delivery costs. | Contextual mapping |
| Şahin and Karabey, 2025, Türkiye [26] | EURO | Upper middle income | Full comparative economic evaluation | CEA, full economic evaluation with DALY-based ICER reporting, ICER per case averted, ICER per death averted, and ICER per life-year saved. | No specific grant. The study reported no specific funding from any public, commercial, or not-for-profit agency. Ethics approval from Istanbul University Clinical Research Ethics Committee, No. 2019/513, 12 April 2019. | No. Non-industry study. Authors declared no competing interests. Institutional affiliations included the University of Health Sciences, the Istanbul Medipol University Epidemiology Program, and the Istanbul University Faculty of Medicine. | Scenario 1: bivalent Cervarix GSK or quadrivalent Gardasil Merck HPV vaccination, with vaccine prices of US$8.5 for bivalent and US$9.6 for quadrivalent, based on PAHO procurement data 2018. Scenario 2: nonavalent Gardasil 9 Merck HPV vaccination, with vaccine price US$16.81 based on CDC and WHO 2018 reference. | 12, primary cohort selected based on Türkiye Td booster dose timing. Td booster coverage at age 12 was 93%. | Girls only, 12-year-old female cohort | Two strategies: Scenario 1, bivalent or quadrivalent HPV vaccination with 80% coverage compared with no vaccination baseline. Scenario 2, nonavalent HPV vaccination with 90% coverage compared with no vaccination baseline. Scenario 2 was also compared incrementally with Scenario 1 for direct head-to-head ICER calculation. | Cervical cancer screening was not explicitly modeled. The study used a vaccine-only CEA in the PRIME framework and noted that combining vaccination with screening would substantially improve outcomes, referencing Özdemir's 2022 Turkish literature review. | Primary synthesis |
| Termrungruanglert et al., 2024, Thailand [57] | SEARO | Upper middle income | Full comparative economic evaluation | CUA, full economic evaluation with QALY-based ICER and cost per QALY gained. | Research grant from the Division of Gynecologic Oncology, Department of Obstetrics and Gynecology, Faculty of Medicine, Chulalongkorn University. IRB approval not required. | No. Non-industry study. The authors declared no conflicts of interest. Institutional affiliations included Chulalongkorn University and King Chulalongkorn Memorial Hospital, Bangkok, and Srinakharinwirot University, Nakhon Nayok, Thailand. | Nine strategies: 1-dose and 2-dose bivalent Cervarix GSK, bivalent Cecolin Innovax, quadrivalent Gardasil Merck, and nonavalent Gardasil 9 Merck, plus no vaccination. 9vHPV cost based on procurement price. | 12, a school-based cohort of 12-year-old girls | Girls only, 12-year-old female cohort | Nine vaccination strategies: single dose and 2 dose 2vHPV Cervarix, 2vHPV Cecolin, 4vHPV, and 9vHPV, plus no vaccination. All strategies were assessed in conjunction with primary HPV screening, with incremental comparison across strategies. | Primary HPV testing screening was modeled for women aged 30 to 65 every 5 years, with 53.9% coverage. | Primary synthesis |
| Gao et al., 2025, Nigeria [47] | AFRO | Lower middle income | Full comparative economic evaluation | CUA, full economic evaluation with QALY-based ICER, cost per QALY gained, and health system and societal perspectives. | No specific grant stated. Authors were affiliated with Georgetown University, the University of Saskatchewan, the Behavioral Insights Lab, and the World Bank. The study was not a human subjects study, and IRB approval was not required. | No. Non-industry study. Authors included academic and World Bank affiliations. No competing interests indicated. | Single dose quadrivalent HPV vaccine, Gardasil 4. Gavi price is US$4.50 per dose. Wastage was assumed at 5%. | 9 to 14, school-based national program cohort | Girls only9- to 14-year-old female cohort | Two scenarios: a quadrivalent HPV vaccination program with 76% coverage versus the status quo (no vaccination program). | Cervical cancer screening was not modeled. Opportunistic screening in Nigeria was acknowledged. The analysis was a vaccine-only CEA. | Primary synthesis |

***Classification key:*** *Full comparative economic evaluation, primary synthesis, n = 33; contextual economic evidence record, contextual mapping only, n = 11.*
***Abbreviations:*** *AFRO, African Region; AMRO, Region of the Americas; EMRO, Eastern Mediterranean Region; EURO, European Region; SEARO, South-East Asia Region; WPRO, Western Pacific Region; LMIC, low- and middle-income country or countries; CEA, cost-effectiveness analysis; CUA, cost-utility analysis; CBA, cost-benefit analysis; BIA, budget impact analysis; CCA, cost-consequence analysis; DALY, disability-adjusted life year; ICER, incremental cost-effectiveness ratio; NNV, number-needed-to-vaccinate; NPV, net present value; NR, not reported; QALY, quality-adjusted life year; WHO, World Health Organization; 2v, bivalent HPV vaccine; 4v, quadrivalent HPV vaccine; 9v, nonavalent HPV vaccine. Mixed: multi-country record including more than one income group.*

***Row shading:*** *Green shading indicates full comparative economic evaluations included in the primary synthesis. Amber shading indicates contextual economic evidence records included for contextual mapping only. Row shading is used only as a visual aid; the classification key should be used as the authoritative interpretation if colors are not visible in print or journal formatting.*
