# Supplementary material for: Methodological drivers, reporting completeness, and transferability in HPV vaccination economic evaluations: A scoping review
Source: PLoS One. 2026 Oct 14;21(10):e0360732. doi: 10.1371/journal.pone.0360732 (PMC13667800; doi:10.1371/journal.pone.0360732)
Supplement: Supplementary file 2 [file pone.0360732.s002.docx]

**S2 Table. Model and economic evaluation characteristics of included records.**

| **Author(s), Year, Country** | **Model type** | **Transmission structure** | **Time horizon** | **Herd immunity incorporated (Y/N/NA)** | **Cross-protection** | **Calibration** | **Sensitivity/uncertainty analysis** | **Analytic perspective** | **Discount rate, costs (%)** | **Discount rate, outcomes (%)** |
| --- | --- | --- | --- | --- | --- | --- | --- | --- | --- | --- |
| Goldie et al.,  2004,  United States [3] | Markov model | Static cohort | Lifetime | N, not incorporated | No | Partial | Deterministic one-way sensitivity analysis | Societal | 3 | 3 |
| Elbasha et al.,  2007,  United States [6] | Dynamic transmission model, population-based deterministic compartmental model, with epidemiological model component | Dynamic transmission, compartmental | 100 years, planning horizon | Y, dynamic transmission model | No, independent natural histories assumed | Yes, HPV prevalence and cervical cancer incidence | Deterministic one-way and multivariate sensitivity analysis | Healthcare payer | 3 | 3 |
| Boot et al.,  2007,  The Netherlands [34] | Cohort-based Markov model | Static cohort | Lifetime | N, not incorporated | No | Partial | Deterministic only | Limited societal, includes friction-cost productivity losses | 4 | 1.5 |
| French et al.,  2007,  Finland [60] | Dynamic transmission model, single-type HPV 16 model | Dynamic transmission, compartmental | 2000 to 2060, 60 years | Y, dynamic transmission model | NA, epidemiological model | Yes, Finnish HPV prevalence | Deterministic only | NA, epidemiological model only | NA | NA |
| Kulasingam et al., 2008,  United Kingdom [35] | Markov state-transition model | Static cohort | Lifetime, age 12 to 85 | N, not incorporated; acknowledged as a limitation | No, 10% coinfection with other high-risk HPV in sensitivity analysis | Yes, partial calibration using UK HPV prevalence, cervical cancer lifetime risk, and incidence | No PSA, explicitly noted as a limitation | Healthcare payer, UK NHS | 3.5 | 3.5 |
| Coupé et al.,  2009,  The Netherlands [36] | Multi-type Markov model with parallel chains | Static cohort | Lifetime, age 12 to 100 | N, not incorporated; acknowledged as a limitation | No in base case; sensitivity analysis explored 50% to 90% protection against HPV31/45 | Yes, HPV genotype and CIN data | Deterministic scenario analysis, 39 alternative parameter sets | Healthcare payer, Dutch direct medical costs | 4 | 1.5 |
| Kim et al.,  2009,  United States [9] | Individual-based first-order Monte Carlo simulation | Individual-based microsimulation | Lifetime, from age 9 entry to death | N, not incorporated | No, cross-protective effects explicitly not included | Yes, likelihood-based calibration to US empirical data | Formal PSA | Societal, primary analysis, including patient time and transportation | 3 | 3 |
| Reynales-Shigematsu et al., 2009,Mexico [37] | Markov model | Static cohort | Lifetime, age 12 to 85 | N, not incorporated | No | Partial, adapted from Kulasingam-Myers and calibrated to Mexican age-specific cervical cancer mortality | Deterministic only | Healthcare payer, Mexican Ministry of Health/public sector | 3 | 3 |
| Dasbach et al.,  2010, Hungary [38] | Dynamic transmission model for quadrivalent HPV types 6/11/16/18, adapted from a previously developed US quadrivalent HPV vaccination model | Dynamic transmission, compartmental | 100 years | Y, dynamic transmission model | No, cross-protection against non-vaccine HPV types was not modeled; acknowledged as a conservative assumption | Partial, Hungarian adaptation validated against cervical cancer incidence; genital-warts epidemiological data were unavailable for calibration | Deterministic sensitivity analysis and pessimistic scenario analysis; no formal PSA reported | Healthcare payer, Hungarian National Health Insurance Fund Administration, NHIFA | 5 | 5 |
| Demarteau et al.,  2010,  France; Ireland; Italy [29] | Markov cohort model, lifetime model with 1-year cycles | Static cohort | Lifetime, 95 years, cohort age 12 followed through life | N, static cohort model; acknowledged as a limitation | Overall efficacy irrespective of type | No | Deterministic scenario and threshold sensitivity analysis on frontier lines | Healthcare payer, third-party payer, France, Ireland, and Italy | France: 3; Ireland: 3.5; Italy: 3 | France: 1.5; Ireland: 3.5; Italy: 3 |
| Capri et al.,  2011,  Italy [48] | Prevalence-based steady-state model | Static cohort | 1 year at steady state, not lifetime | N, steady-state prevalence-based model | Overall efficacy irrespective of type | No | Univariate and probabilistic sensitivity analysis | Healthcare payer, Italian SSN | 0, no discount applied due to 1-year steady-state evaluation | 0 |
| Praditsitthikorn et al., 2011,  Thailand [21] | Semi-Markov model | Semi-Markov cohort model, herd immunity not captured | Lifetime, 100-year period, cohort age 15 followed through life | N, not incorporated; acknowledged as a limitation | No, explicitly excluded | Yes, Thai HPV and cervical cancer incidence | Formal PSA, 10,000 simulations | Limited societal, payer plus patient time and transport | 3 | 3 |
| Schobert et al.,  2012,  Germany [27] | Dynamic transmission model, multi-type model | Dynamic transmission, compartmental | 100 years, lifetime, base-case analysis from third-party payer perspective | Y, dynamic transmission model | Scenario only | Partial | Deterministic only | Healthcare payer, German statutory health insurance, SHI | 3 | 3 |
| Termrungruanglert et al., 2012, Thailand [39] | Markov state-transition model | Static cohort | Lifetime, age 12 to 100 | N, not incorporated; acknowledged as a limitation | No | No | Formal PSA, Monte Carlo, 10,000 simulations | Healthcare payer, Thailand | 3 | 3 |
| Aponte-González et al., 2013, Colombia [22] | Markov model | Static cohort | Lifetime, age 12 to 76, Colombian female life expectancy | N, not incorporated; acknowledged as a limitation | Yes, cross-protection against non-vaccine high-risk HPV types: bivalent approximately 30%, quadrivalent approximately 25% | Partial, Colombian DALY | Formal PSA, 1,000 iterations | Limited societal, payer plus patient transport and home care | 3 | 3 |
| Berkhof et al.,  2013,  CEECA region (28 countries), Slovenia, Poland, Georgia [40] | Two-part approach. First, a simplified threshold formula for 28 countries. Second, individual-based microsimulation for three countries. | Individual-based microsimulation | Lifetime, per cohort to mortality | N, not incorporated; acknowledged as a limitation | No, HPV16/18 only; 70% cervical cancer prevention assumption | Yes, CEECA epidemiology | Deterministic univariate and multivariate sensitivity analysis | Healthcare payer, CEECA national health systems | 3 | 3 |
| Bresse et al.,  2013,  France [50] | Two-stage indirect approach. Dynamic transmission model adapted from Elbasha-Dasbach 2010 for HPV 16, 18, 6, and 11, with post-processing for non-vaccine HPV cross-protection. | Dynamic transmission, compartmental | 100 years, 2012 to 2112 | Y, dynamic transmission model | Overall efficacy irrespective of type | Partial | Deterministic only | Healthcare payer, France, and societal scenario | 4 | 4 |
| Kim et al.,  2013a,  Sub-Saharan Africa  48 countries; Uganda, South Africa [23] | Two-tier approach. First, simple cohort simulation for 48 countries. Second, individual-based microsimulation for Uganda and South Africa, using the same framework as Kim et al. 2009. | Individual-based microsimulation | Lifetime, per cohort to mortality | N, not incorporated; acknowledged as a limitation | No, vaccine-type only | Yes, regional sub-Saharan African epidemiology | Deterministic and scenario analyses; calibration parameter-set uncertainty, 50 good-fitting sets | Healthcare payer, sub-Saharan African national health systems | 3 | 3 |
| Kim et al.,  2013b,  EMENA region 20 countries; Algeria, Lebanon, Turkey [24] | Two-tier approach. First, simple cohort simulation for 20 EMENA countries. Second, individual-based microsimulation for Algeria, Lebanon, and Turkey, using the same framework as Kim et al. 2009 and Kim et al. 2013 SSA. | Individual-based microsimulation | Lifetime, per cohort to mortality | N, not incorporated; acknowledged as a limitation | No, explicitly assumed fully susceptible to non-16/18 types | Yes, regional EMENA epidemiology | Deterministic and scenario analyses; calibration parameter-set uncertainty, good-fitting sets | Healthcare payer, EMENA national health systems | 3 | 3 |
| Jit et al., 2014,  England and Canada [7] | Two transmission-dynamic models in parallel: an English compartmental transmission-dynamic model and the Canadian HPV-ADVISE individual-based transmission-dynamic model | Dynamic transmission, compartmental and individual-based models in parallel | 70 years post-vaccination introduction | Y, dynamic transmission models | Yes, limited cross-protection against HPV31/33/45/52/58, 5.5% to 46.2% | No | Deterministic only | NA, number-needed-to-vaccinate, NNV, clinical impact analysis | 5 | 5 |
| Bonanni et al.,  2015,  Italy [49] | Lifetime Markov cohort model adapted from the Demarteau et al. 2013 framework for the Italian setting | Static cohort | Lifetime, 95 cycles | N, static cohort model; acknowledged as a limitation | Overall efficacy irrespective of type | No | Deterministic only | NA, cases-prevented model; no economic evaluation | 5 | 5 |
| Aljunid et al.,  2016,  Malaysia [41] | Markov cohort model with a 95-year lifetime horizon, not a dynamic transmission model | Static cohort | Lifetime, 95 cycles for 13-year-old girls | N, static cohort model; acknowledged as a limitation | No | No, adapted model | Deterministic only | Healthcare payer, Malaysian MOH | 3 | 3 |
| Setiawan et al.,  2016a,  Indonesia [42] | Population-based Markov model with simplified three-state structure, including susceptible, cervical cancer, and death | Static cohort | Lifetime, age 12 to 100 | N, static cohort model; acknowledged as a limitation | No | Partial, Indonesian epidemiology | Formal PSA | Healthcare payer, Indonesian Ministry of Health | 3 | 3 |
| Setiawan et al.,  2016b,  Indonesia [54] | Two-stage approach. First, a population-based Markov model was adopted from Setiawan et al. 2016a, with three states: susceptible, cervical cancer, and death. Second, a fiscal analytic framework used discounted cash-flow net present value calculation to translate clinical outcomes into government tax revenue and social-security gains or losses. | Static cohort Markov disease model linked to fiscal NPV framework; no transmission dynamics | Lifetime, 58 years | N, not incorporated; acknowledged as a limitation | NA, fiscal NPV | NA, fiscal NPV | Univariate deterministic sensitivity analysis | Government-fiscal, Indonesian fiscal NPV | 3 | 3 |
| Vargas Parada and Veerman, 2016, Chile [33] | Cohort multistate life-table-based Markov model with DisMod II for case-fatality estimation | Static cohort | Lifetime, age 30 to 100 | N, not incorporated; acknowledged as a limitation | No | Partial, Chilean data and DisMod II | Deterministic only | Healthcare payer, Chilean Ministry of Health | 3 | 3 |
| Kosen et al.,  2017,  Indonesia [43] | Dynamic transmission model was adapted from the Elbasha et al. 2007 quadrivalent HPV vaccination strategies model | Dynamic transmission, compartmental | 100 years | Y, dynamic transmission model | No, Elbasha 6/11/16/18 framework; cross-protection not modelled | Partial | No PSA, transmission dynamic model, explicitly stated | Healthcare payer, Indonesian Ministry of Health | NR | NR |
| Mennini et al.,  2017, Italy [44] | Deterministic dynamic ODE-based susceptible-infected-recovered-susceptible, SIRS, transmission model adapted from Elbasha et al. 2007 and Elbasha et al. 2010 boys-and-men extension frameworks | Dynamic transmission, compartmental | 100 years | Y, dynamic transmission model | No, 9 types modelled directly; no cross-protection assumed in base case | Yes, Italian epidemiology adapted for 9v analysis | Formal PSA | Healthcare payer, Italian SSN | 3 | 3 outcomes, 1.5% in sensitivity analysis only |
| Chesson et al.,  2018,  United States [28] | Deterministic dynamic population-based transmission model based on Chesson 2008/2011 and the Chesson 2016 9vHPV extension, with simplified structure compared with Elbasha 2007 and HPV-ADVISE | Dynamic transmission, compartmental | 100 years | Y, dynamic transmission model | No, 9 vaccine types modelled directly; no additional cross-protection | Yes, US national surveillance | Formal PSA, 5,000 simulations | Healthcare payer, US payer, and societal scenario | 3 | 3 |
| Yaghoubi et al.,  2018, Iran [32] | CERVIVAC v1.1.60 standardized LMIC HPV vaccination cost-effectiveness tool developed in collaboration with the London School of Hygiene and Tropical Medicine and the Harvard Center for Health Decision Science | Static cohort | 10-year programme horizon, with lifetime cost and benefit tracking from age 9 to death | N, static model; herd immunity not incorporated and acknowledged as a model limitation | No | No, CERVIVAC standard model using Iranian epidemiological and cost inputs | Deterministic sensitivity and scenario analysis | Healthcare payer, Iranian MOH primary; societal secondary | 3 | 3 |
| Bahr et al.,  2019,  Lebanon [53] | Direct cost-ratio comparison within a cost-benefit analysis framework. The vaccination cost was compared with the cervical cancer management cost. No mathematical disease model and no formal modeled time horizon were used. | NA, CBA cost-ratio analysis | Single year, with break-even point projection over 5 to 10 years | N, not incorporated; no transmission model | NA, CBA cost ratio | NA, CBA cost ratio | Deterministic only | Government/health-system cost perspective; indirect and productivity costs excluded | NA, no discounting, single-year analysis | NA |
| Datta et al.,  2019,  United Kingdom [10] | Individual-based stochastic SIRS-V, susceptible-infected-recovered-susceptible-vaccinated, transmission model with Bayesian MCMC parameter inference, integrated with a continuous-time individual patient simulation economic model | Individual-based transmission, MCMC | 100 years post-vaccination intervention | Y, individual-based transmission model | Type-specific cross-protection | Yes, Bayesian history matching using NATSAL | Formal PSA, Bayesian MCMC | Healthcare payer, UK NHS | 3.5 | 3.5 |
| Lee et al.,  2019,  South Korea [30] | Static Markov model adapted from Demarteau and Standaert 2010 to the Korean setting. Lifetime cohort simulation up to 95 one-year cycles, with a cohort of 253,000 12-year-old girls based on KOSIS 2015. | Static cohort | Lifetime, up to 95 one-year cycles | N, static Markov model; acknowledged as a limitation | Overall efficacy irrespective of type | No | Deterministic only | Healthcare payer, Korean NHIS | 5 | 5 |
| Messoudi et al.,  2019,  Morocco [45] | Static Markov model adapted from the validated Spanish ICO model, Georgalis 2016 and Moriña 2017. The model included 12 health states: healthy, HPV infection, CIN1 to CIN3, FIGO cervical cancer stages I to IV, cancer survival, cervical cancer mortality, and other-cause mortality. It used 1-year transitions and a lifetime simulation. | Static cohort | Lifetime | N, static Markov model; acknowledged as a limitation | No | Partial, Moroccan ICO adaptation | Deterministic only | Healthcare payer, Moroccan MOH, public healthcare perspective | 3 | 3 |
| Smith et al.,  2019,  Canada [55] | OncoSim microsimulation model, version 2.5, developed by the Canadian Partnership Against Cancer, Statistics Canada, and Health Canada. The model used multiple data sources and expert opinions and included cancer-specific diagnostic, treatment, cost, utility, and tax-revenue components. | Microsimulation with incorporated herd-immunity assumptions, OncoSim | Lifetime cohort, girls aged 5 to 10 years in 2015 followed through lifetime simulation | Y, incorporated in OncoSim microsimulation | No, quadrivalent vaccine; HPV16/18 approximately 70% of cervical cancer | Yes, Canadian OncoSim | Deterministic only | Healthcare payer, Canadian provincial | 1.5 | 1.5 |
| Tang et al.,  2019,  Taiwan [31] | Static Markov cohort model adapted from the Demarteau 2012 and Suarez 2008 frameworks. The model used 1-year cycles across a 95-year lifetime horizon and a cohort of 120,000 Taiwanese 12-year-old girls. | Static cohort | Lifetime, 95 one-year cycles to age 107 | N, static Markov model; acknowledged as a limitation | Overall efficacy irrespective of type | No | Formal PSA, 1,000 simulations | Healthcare payer, Taiwan NHI | 3 | 3 |
| Chauhan et al.,  2020,  India [46] | Static Markov model based on previously published validated cervical cancer models, including Kim 2007 multiparameter calibration. The model used 1-year cycles and a lifetime simulation. | Static cohort | Lifetime | N, static Markov model; acknowledged as a limitation | No | Yes, Indian NPCDCS data | Formal PSA, 999 Monte Carlo simulations | Limited societal, payer plus patient out-of-pocket costs; wage losses excluded | 3 | 3 |
| Kitano ,  2022,  Japan [56] | Decision-tree risk-benefit model, not a Markov model. The model estimated lifetime QALY gain and loss for a vaccinated 12-year-old boy, with results reported per 100,000 vaccinated persons. | NA, individual decision-tree risk-benefit analysis | Lifetime | N, not incorporated; decision-tree risk-benefit analysis | No, vaccine-covered HPV-related outcomes only; no cross-protection modelled | No | Deterministic only | Individual, Japanese male risk-benefit decision tree | 3 | 3 |
| Owusu-Edusei et al., 2022,  United Kingdom [25] | Age-structured deterministic heterosexual compartmental disease transmission model based on Elbasha 2007, Dasbach 2008, Daniels 2021, and Majed 2021 frameworks, using an ordinary differential equations system | Dynamic transmission, compartmental | 100 years, starting 2019 | Y, dynamic transmission model | No, 9 vaccine types modeled directly; UV4V versus UV9V comparison | Partial | Deterministic only | Healthcare payer, UK NHS | 3.5 | 1.5 |
| Sargazi et al.,  2022,  Iran [52] | Cost-benefit analysis framework, not a Markov model. The study combined bottom-up prevalence-based cost-of-illness analysis and a willingness-to-pay discrete-choice experiment. Vaccine effectiveness was adjusted for Iranian HPV genotype distribution. | NA, CBA, WTP DCE, and COI | Lifetime, life expectancy of Iranian women 76 years, World Bank 2020 | N, not incorporated; acknowledged as a limitation | Yes, cross-protective effects against non-16/18 high-risk HPV types were included in the vaccine-effectiveness calculation | NA, CBA | Deterministic only | Full societal, cost-benefit analysis plus willingness-to-pay DCE and cost-of-illness including productivity | 3 | 3 |
| Palmer et al.,  2023, Japan [11] | Deterministic dynamic transmission metapopulation model adapted from Elbasha 2007, Dasbach 2008, Insinga 2009, and Cody 2021. The model included separate disease transmission models for distinct demographic groups segmented by age, sex, and sexual activity. | Dynamic transmission, compartmental, metapopulation | 100 years, 2024 to 2124 | Y, dynamic metapopulation model | No, 9 vaccine types modelled directly | Yes, Bayesian history matching | Calibration uncertainty only, 10 Bayesian parameter combinations | Healthcare payer, Japanese MHLW | 3 | 3 |
| Slavkovsky et al.,  2024,  Ethiopia, Guyana, Rwanda, Sri Lanka, Uganda [51] | Cost-of-delivery modeling using primary data from the companion Mvundura 2023 six-country HPV vaccine delivery cost study. The analysis used micro costing methods, evaluated 11 program activities, and distinguished financial from economic costs. | NA, cost-of-delivery only | Annual, single-year reference period 2019, cost-analysis focus | NA, cost-of-delivery analysis with no health outcomes | NA, cost-of-delivery analysis | NA, cost-of-delivery analysis | Deterministic only | Health system, 5 LMICs, delivery cost analysis only | NA, single-year 2019 cost analysis | NA, no health outcomes |
| Şahin & Karabey,  2025, Türkiye [26] | WHO PRIME model, Papillomavirus Rapid Interface for Modelling and Economics. Static cohort model with four modules: country selection, demographic inputs, output generation, and customization. The model used the same framework as Yaghoubi 2018 Iran, Van Minh 2017 Vietnam, Mahumud 2020 Bangladesh, and the Jit 2014 Lancet Global Health 179-country PRIME study. | Static cohort, PRIME | 100 years, post-vaccination lifetime, modeled outputs annual values in 2018 USD presented in Tables 3 and 4 | N, static PRIME model; acknowledged as a limitation | No | No, PRIME standard | Deterministic only | Healthcare payer, Turkish SGK primary; societal secondary | 3 | 3 |
| Termrungruanglert et al., 2024, Thailand [57] | Markov cohort model with eight health states: susceptible, HPV infection, genital wart or CIN1, CIN2/3, cancer, post-treatment cancer, cured, and death. HPV genotypes were grouped into four classes: low-risk HPV 6/11, HPV 16, HPV 18, and other high-risk types. The model used a cohort of 100,000 HPV-free 12-year-old girls. | Static cohort, Markov | Lifetime, cohort followed to death, 1-year cycle | N, static Markov model; acknowledged as a limitation | No, cross-protection effect not analyzed; explicitly stated | Partial, Thai genotype distribution, local utilities and costs, and parameters from prior Thai models and literature | Formal PSA, probabilistic, 10,000 simulations, plus one-way sensitivity analysis | Healthcare provider, direct medical costs only | 3 | 3 |
| Gao et al., 2025, Nigeria [47] | Markov model with six health states: susceptible, cervical cancer, death, and cervical cancer pathways with successful treatment, treatment failure, and no treatment. The model used a cohort of 100,000 girls aged 9 to 14. | Static cohort, Markov | Lifetime, followed to age 100, transition probabilities from IHME age-specific data | N, static Markov model; acknowledged as a limitation | No, HPV16/18 approximately 70% of cervical cancer; overall vaccine efficacy 68.25% against cervical cancer | Partial, Nigeria real-world program cost and coverage data, and IHME age-specific incidence and mortality | Formal PSA, Monte Carlo, 10,000 iterations, Beta/Gamma distributions, plus one-way tornado analysis | Health system and societal, Second Panel on Cost-Effectiveness recommendation | 3 | 3 |

***Classification key:*** *Full comparative economic evaluation, primary synthesis, n = 33; contextual economic evidence records, contextual mapping only, n = 11. Contextual economic evidence records were included for descriptive mapping only and were not included in the primary methodological credibility or transferability synthesis.*

***Herd immunity incorporated:*** *Y = incorporated in the model; N = not incorporated; NA = not applicable to the record design. Additional notes are included only where the publication explicitly described the modeling basis or identified non-incorporation as a limitation.*

***Transmission structure:*** *NA was used when the record did not include a transmission or disease-progression modeling structure, such as fiscal NPV analysis, cost-benefit cost-ratio analysis, decision-tree risk-benefit analysis, cost-of-delivery analysis, or cost-of-illness and willingness-to-pay analysis.*

***Cross-protection:*** *“No” indicates that cross-protection was not included or not modeled. “Yes” indicates explicit cross-protection assumptions. “Overall efficacy irrespective of type” indicates that the study used broader vaccine-efficacy assumptions rather than explicit type-specific cross-protection. NA was used where cross-protection was not applicable to the record design.*

***Calibration:*** *“Yes” indicates explicit calibration to local or regional epidemiological or model-input data. “Partial” indicates adaptation or partial use of local, regional, or external calibration inputs. “No” indicates no clear calibration reported. NA indicates not applicable to the record design.*

***Sensitivity/uncertainty analysis:*** *This column reports the uncertainty analysis described by each publication, including deterministic sensitivity analysis, scenario analysis, calibration uncertainty, formal probabilistic sensitivity analysis, or no PSA where explicitly stated.*

***Discount rates:*** *Rates are reported where used by the original publication. NA, not applicable; NR, not reported.*

***Abbreviations:*** *CBA, cost-benefit analysis; CEECA, Central and Eastern Europe and Central Asia; CERVIVAC, standardized HPV vaccination cost-effectiveness tool; CIN, cervical intraepithelial neoplasia; COI, cost of illness; DALY, disability-adjusted life year; DCE, discrete-choice experiment; EMENA, Eastern Mediterranean, Europe, and North Africa; FIGO, International Federation of Gynecology and Obstetrics; HPV, human papillomavirus; HPV-ADVISE, named individual-based HPV transmission model; ICO, Catalan Institute of Oncology; IHME, Institute for Health Metrics and Evaluation; KOSIS, Korean Statistical Information Service; LMIC, low- and middle-income country or countries; MCMC, Markov chain Monte Carlo; MHLW, Ministry of Health, Labour and Welfare; MOH, Ministry of Health; NA, not applicable; NATSAL, National Survey of Sexual Attitudes and Lifestyles; NHI, National Health Insurance; NHIFA, National Health Insurance Fund Administration; NHIS, National Health Insurance Service; NHS, National Health Service; NNV, number-needed-to-vaccinate; NPCDCS, National Programme for Prevention and Control of Cancer, Diabetes, Cardiovascular Diseases and Stroke; NPV, net present value; NR, not reported; ODE, ordinary differential equation; PRIME, Papillomavirus Rapid Interface for Modelling and Economics; PSA, probabilistic sensitivity analysis; QALY, quality-adjusted life year; SGK, Social Security Institution; SHI, statutory health insurance; SIRS, susceptible-infected-recovered-susceptible; SIRS-V, susceptible-infected-recovered-susceptible-vaccinated; SSA, sub-Saharan Africa; SSN, Servizio Sanitario Nazionale; UK, United Kingdom; US, United States; USD, United States dollars; UV4V, universal vaccination with 4-valent HPV vaccine; UV9V, universal vaccination with 9-valent HPV vaccine; WTP, willingness to pay.*

***Row shading:*** *Green shading indicates full comparative economic evaluations included in the primary synthesis. Amber shading indicates contextual economic evidence records included for contextual mapping only. Row shading is used only as a visual aid; the classification key should be used as the authoritative interpretation if colors are not visible in print or journal formatting.*

**S2 Table (continued). Record-level structured methodological credibility appraisal (33 full comparative economic evaluations; 11 contextual records shown for completeness).**

| **Author(s), Year, Country** | **Comparator appropriateness** | **Model structure suitability** | **Local calibration and validation** | **Herd immunity modeling** | **Uncertainty analysis adequacy** | **Threshold justification** |
| --- | --- | --- | --- | --- | --- | --- |
| Goldie et al., 2004, United States [3] | Partial | Adequate | Partial | Partial | Partial | Adequate |
| Elbasha et al., 2007, United States [6] | Adequate | Adequate | Adequate | Adequate | Partial | Adequate |
| Boot et al., 2007, The Netherlands [34] | Partial | Partial | Partial | Partial | Partial | Partial |
| French et al., 2007, Finland [60] | — | — | — | — | — | — |
| Kulasingam et al., 2008, United Kingdom [35] | Adequate | Adequate | Adequate | Partial | Limited | Adequate |
| Coupé et al., 2009, The Netherlands [36] | Adequate | Adequate | Adequate | Partial | Adequate | Adequate |
| Kim et al., 2009, United States [9] | Adequate | Adequate | Adequate | NA | Adequate | Adequate |
| Reynales-Shigematsu et al., 2009, Mexico [37] | Partial | Partial | Partial | NA | Limited | Partial |
| Dasbach et al., 2010, Hungary [38] | Adequate | Adequate | Partial | Adequate | Adequate | Adequate |
| Demarteau et al., 2010, France; Ireland; Italy [29] | Partial | Partial | Limited | NA | Partial | Partial |
| Capri et al., 2011, Italy [48] | — | — | — | — | — | — |
| Praditsitthikorn et al., 2011, Thailand [21] | Adequate | Adequate | Adequate | NA | Adequate | Adequate |
| Schobert et al., 2012, Germany [27] | Adequate | Adequate | Partial | Adequate | Limited | Partial |
| Termrungruanglert et al., 2012, Thailand [39] | Partial | Partial | Limited | NA | Adequate | Partial |
| Aponte-González et al., 2013, Colombia [22] | Adequate | Adequate | Adequate | NA | Adequate | Adequate |
| Berkhof et al., 2013, CEECA region [40] | Adequate | Adequate | Adequate | NA | Partial | Partial |
| Bresse et al., 2013, France [50] | — | — | — | — | — | — |
| Kim et al., 2013a, Sub-Saharan Africa [23] | Adequate | Adequate | Adequate | NA | Partial | Partial |
| Kim et al., 2013b, EMENA region [24] | Adequate | Adequate | Adequate | NA | Partial | Partial |
| Jit et al., 2014, England and Canada [7] | — | — | — | — | — | — |
| Bonanni et al., 2015, Italy [49] | — | — | — | — | — | — |
| Aljunid et al., 2016, Malaysia [41] | Limited | Partial | Limited | NA | Limited | Partial |
| Setiawan et al., 2016a, Indonesia [42] | Adequate | Partial | Partial | Limited | Adequate | Partial |
| Setiawan et al., 2016b, Indonesia [54] | — | — | — | — | — | — |
| Vargas Parada and Veerman, 2016, Chile [33] | Partial | Adequate | Partial | NA | Limited | Limited |
| Kosen et al., 2017, Indonesia [43] | Adequate | Adequate | Partial | Adequate | Limited | Partial |
| Mennini et al., 2017, Italy [44] | Adequate | Adequate | Adequate | Adequate | Adequate | Adequate |
| Chesson et al., 2018, United States [28] | Adequate | Adequate | Adequate | Adequate | Adequate | Adequate |
| Yaghoubi et al., 2018, Iran [32] | Adequate | Limited | Limited | NA | Partial | Partial |
| Bahr et al., 2019, Lebanon [53] | — | — | — | — | — | — |
| Datta et al., 2019, United Kingdom [10] | Adequate | Adequate | Adequate | Adequate | Adequate | Adequate |
| Lee et al., 2019, South Korea [30] | Partial | Partial | Partial | Partial | Limited | Adequate |
| Messoudi et al., 2019, Morocco [45] | Adequate | Partial | Partial | NA | Limited | Partial |
| Smith et al., 2019, Canada [55] | — | — | — | — | — | — |
| Tang et al., 2019, Taiwan [31] | Partial | Partial | Partial | Partial | Adequate | Adequate |
| Chauhan et al., 2020, India [46] | Adequate | Adequate | Adequate | NA | Adequate | Adequate |
| Kitano, 2022, Japan [56] | — | — | — | — | — | — |
| Owusu-Edusei et al., 2022, United Kingdom [25] | Adequate | Adequate | Adequate | Adequate | Partial | Adequate |
| Sargazi et al., 2022, Iran [52] | — | — | — | — | — | — |
| Palmer et al., 2023, Japan [11] | Adequate | Adequate | Adequate | Adequate | Adequate | Adequate |
| Slavkovsky et al., 2024, Ethiopia et al. [51] | — | — | — | — | — | — |
| Şahin and Karabey, 2025, Türkiye [26] | Partial | Limited | Partial | NA | Partial | Limited |
| Termrungruanglert et al., 2024, Thailand [57] | Adequate | Adequate | Partial | NA | Adequate | Adequate |
| Gao et al., 2025, Nigeria [47] | Adequate | Partial | Adequate | NA | Adequate | Limited |

***Note:*** *Each domain was rated Adequate, Partial, Limited, or NA (not applicable) using the criteria described in Methods Section 3.5.3. Aggregate domain-level results are reported in manuscript Table 4. Blue shading indicates full comparative economic evaluations (n = 33, primary synthesis); orange shading indicates contextual economic evidence records (n = 11, descriptive mapping only). The herd immunity modeling domain uses NA for evaluations where herd effects were not central to the decision problem. Contextual records are marked with an em dash (—) because the structured credibility appraisal was applied only to the 33 full comparative economic evaluations.*
