# Supplementary material for: Methodological drivers, reporting completeness, and transferability in HPV vaccination economic evaluations: A scoping review
Source: PLoS One. 2026 Oct 14;21(10):e0360732. doi: 10.1371/journal.pone.0360732 (PMC13667800; doi:10.1371/journal.pone.0360732)
Supplement: Supplementary file 3 [file pone.0360732.s003.docx]

**S3 Table. Economic outcomes and drivers of variation in included records.**

| **Author(s), Year, Country** | **Primary outcome metric** | **Base-case economic**  **result** | **Decision threshold**  **and benchmark** | **Threshold source** | **Main conclusion** | **Key uncertainty drivers** | **Reported conclusion** | **Methodological and**  **comparability notes** |
| --- | --- | --- | --- | --- | --- | --- | --- | --- |
| Goldie et al.,  2004,  United States [3] | QALY | Vaccination plus triennial conventional cytology starting at age 25 versus the next best comparator: US$58,500/QALY, assuming 90% vaccine efficacy. Vaccination alone added to current screening: US$20,600–US$33,700/QALY, depending on 70–100% vaccine efficacy. | The US willingness-to-pay benchmark of US$50,000 to US$75,000 per QALY. | Country-specific US willingness to pay convention. | Cost-effective under the study assumptions. | Duration of vaccine efficacy, proportion of persistent HPV in older women due to new infection versus reactivation, screening frequency, screening age, screening cost, and quality-of-life decrements from positive screening. | HPV vaccination was projected to be a cost-effective use of health care resources when it allowed later initiation and less frequent cervical screening; vaccination alone added to current screening was estimated at approximately $20,600–$33,700/QALY, depending on vaccine efficacy assumptions. | Pre-licensure US static Markov cost-effectiveness analysis from a societal perspective. The study supported HPV vaccination as cost-effective relative to the US willingness-to-pay benchmarks used in the study. |
| Elbasha et al.,  2007,United States [6] | QALY | Girls aged 12 years plus female catch-up vaccination: US$4,666/QALY. Girls and boys aged 12 years plus female and male catch-up vaccination: US$45,056/QALY. | No single formal US threshold was used. The study benchmarked results against commonly accepted medical interventions in the Tufts CEA Registry. | US benchmark based on commonly accepted medical interventions in the Tufts Cost-Effectiveness Analysis Registry. | Cost-effective under the study assumptions. | Duration of protection, vaccine coverage, quality-of-life weights, vaccine cost, and herd-immunity contribution. A 10-year protection duration substantially increased ICERs, and lower coverage made male vaccination more efficient. | In a setting with organized cervical cancer screening, quadrivalent HPV vaccination was projected to reduce genital warts, CIN, and cervical cancer, improve quality of life and survival, and be cost-effective; the girls-only plus female catch-up strategy was estimated at $4,666/QALY, while broader female-and-male strategies had higher ICERs. | Industry-funded dynamic transmission cost-effectiveness analysis that contributed to an early quadrivalent HPV gender-neutral vaccination modeling framework. |
| Boot et al.,  2007,  The Netherlands [34] | LYG; QALY reported, but not the primary outcome | Scenario A-0, no boosters, €50/dose: €12,000/LYG. Scenario A-0, €100/dose: €24,000/LYG. Scenario A-4, four boosters, €100/dose: €39,500/LYG. | Dutch preventive intervention benchmark of €20,000 per QALY. Dutch GDP per capita was also referenced at €31,000. | Country-specific Dutch preventive intervention benchmark. Dutch GDP per capita was also referenced. | Cost-effective under the study assumptions. | Vaccine price, number of boosters, vaccine efficacy, and discount rate. Vaccine efficacy was conservatively set at 80%, and discount rates differed between Dutch guidelines and a uniform 4% rate. | Universal vaccination of preadolescent girls was projected to have an acceptable cost-effectiveness profile in the Netherlands under conservative assumptions, with estimates ranging from approximately €12,000/LYG to €39,500/LYG depending on vaccine price and booster assumptions. | Hybrid HTA-Markov analysis with friction-cost productivity inclusion and lower CHEERS reporting completeness. |
| French et al.,  2007,  Finland [60] | HPV16-associated cervical cancer cases prevented — epidemiological outcome only; no formal economic outcome | Not applicable; no formal ICER calculated. Epidemiological transmission model only; cost-effectiveness was explicitly excluded. | Not applicable. This was an epidemiological model only. No formal economic threshold was used. | Not applicable. Epidemiological model only. No formal economic threshold was used. | Cost-effectiveness was not assessed. The record was an epidemiological model and explicitly excluded cost-effectiveness analysis. | Age at vaccination, gender-neutral strategy, catch-up duration of 3, 6, 9, or 12 years, sexual behavior assumptions, and duration of natural immunity. | Vaccination before sexual debut was projected to maximize long-term epidemiological impact; catch-up programs accelerated population benefits, and vaccinating males prevented additional cases, although the incremental effect was smaller than increasing female coverage. | Single-type HPV16 dynamic transmission epidemiological model without economic outcomes. |
| Kulasingam et al.,  2008,  United Kingdom [35] | QALY; LYS also reported | Vaccination plus screening versus screening alone: £21,059/QALY and £34,687/LYS in the base case, assuming lifetime duration, 85% coverage, and 98% efficacy. | UK NICE threshold of £30,000 per QALY. | Country-specific UK NICE threshold. | Cost-effective under the study assumptions. | Duration of vaccine protection, booster requirement, discount rate, and combined worst-case assumptions. A 10-year protection duration increased the ICER to £68,417/QALY, and the multivariate worst-case scenario increased it to £84,925/QALY. | Adding quadrivalent HPV vaccination to the current UK cervical screening was projected to be cost-effective for reducing cervical cancer burden, with a base-case estimate of £21,059/QALY. | UK NHS static Markov cost-effectiveness analysis supporting national program implementation. |
| Coupé et al.,  2009,  The Netherlands [36] | QALY; cervical cancer cases and deaths also reported | Vaccination plus screening versus screening only: €19,429/QALY in the base case, assuming 95% efficacy, lifelong protection, 85% coverage, €125/dose, and no booster. Across 39 alternative parameter sets, estimates ranged from approximately €11,000 to €25,000/QALY. | The Dutch preventive intervention threshold of €20,000 per QALY. | Country-specific Dutch preventive intervention threshold. | Cost-effective under the study assumptions. | Waning vaccine efficacy, vaccine price, booster requirement, and vaccine efficacy. Fast waning raised the ICER to €35,000/QALY. Vaccine price had a linear relationship with the ICER. Booster requirement raised the ICER to €23,000/QALY, and reducing vaccine efficacy from 95% to 85% raised the ICER by 15%. | The model supported the implementation of HPV16/18 vaccination for young women alongside cervical cancer screening in the Netherlands, with a base-case ICER of €19,429/QALY, results sensitive to waning protection, vaccine price, and booster assumptions. | Industry-funded 14-type Markov analysis with extensive Dutch local calibration. |
| Kim et al.,  2009,  United States [9] | QALY | Vaccination at ages 35–45 years was not cost-effective. ICERs ranged from US$116,950 to US$272,350/QALY for cytology with HPV triage and from US$193,690 to US$381,590/QALY for combined cytology and HPV testing. Most strategies were dominated or weakly dominated by screening alone. | US benchmark range of US$50,000 to US$100,000 per QALY, based on commonly cited willingness to pay and accepted intervention benchmarks. | Country-specific US willingness to pay convention based on multiple accepted intervention and willingness to pay sources. | Cost-effective for adolescent vaccination, but not cost-effective for screened women aged 35 to 45 years. | Vaccine efficacy in unexposed women, waning protection, vaccine cost, and uncertainty about prior exposure status in older women. At 70% efficacy, ICERs increased by approximately 50% and exceeded US$400,000/QALY. Five-year waning exceeded US$400,000/QALY, and 10-year waning exceeded US$200,000/QALY. Changing vaccine cost from US$250 to US$750 changed ICERs three- to four-fold. | HPV vaccination of screened women older than 30 years produced relatively small average health gains and less attractive cost-effectiveness ratios than established US interventions, with ICERs ranging from approximately $116,950 to $381,590/QALY depending on age and screening strategy. | Microsimulation analysis challenging the economic value of older-women catch-up vaccination at prevailing US prices. |
| Reynales-Shigematsu et al.,  2009,  Mexico [37] | LYS; cervical cancer cases and deaths averted also reported | Vaccine alone versus no intervention: US$68/LYS at US$45 for the three-dose vaccine series. Vaccine plus Pap smear every three years versus vaccine alone: US$15,935/LYS. | WHO-CHOICE GDP-based benchmark using Mexican GDP per capita of US$6,178. | WHO-CHOICE GDP-based threshold using Mexican GDP per capita. | Cost-effective under the study assumptions. | Vaccine cost, age at vaccination, duration of vaccine efficacy, and vaccine coverage. Vaccine cost was linearly related to the ICER, and at least 30% coverage was required for a cost-effective ratio. | Quadrivalent HPV vaccination was projected to reduce cervical cancer burden and to be very cost-effective in Mexico if the vaccine price was sufficiently low; vaccine-only prevention at $45 for three doses was estimated at $68/LYS, and public implementation was considered most relevant if the dose price was no more than $15. | Industry-funded Mexican cost-effectiveness analysis adapting the Kulasingam framework. |
| Dasbach et al.,  2010,  Hungary [38] | QALY; cervical cancer, CIN, genital warts, and deaths averted also reported | Routine vaccination of 12-year-old girls versus no vaccination: €9,577/QALY. Routine vaccination plus 12–24-year catch-up versus routine vaccination alone: €10,646/QALY. | WHO-CHOICE GDP-based benchmark. An ICER below one times GDP per capita was classified as very cost-effective. An ICER between one and three times GDP per capita was classified as cost-effective. An ICER above three times GDP per capita was classified as not cost-effective. Hungarian 2006 GDP per capita was €12,098. | WHO-CHOICE GDP-based thresholds using Hungarian GDP per capita, applied because Hungary had no formal national cost-effectiveness threshold. | Cost-effective under the study assumptions. | Pessimistic combined assumptions, duration of vaccine protection, removal of HPV 6 and 11 protection, and QALY weights. The pessimistic scenario increased the catch-up ICER to €40,083/QALY. A 10-year protection scenario weakly dominated routine vaccination and increased the catch-up ICER to €23,291/QALY. Removing HPV 6 and 11 protections increased the routine ICER by 75% to €16,880/QALY. | Quadrivalent HPV vaccination was projected to reduce cervical cancer, CIN, and genital warts in Hungary and was considered cost-effective under WHO-CHOICE thresholds, with ICERs of €9,577/QALY for routine vaccination and €10,646/QALY for routine vaccination plus catch-up. | Industry-funded Hungarian dynamic cost-effectiveness analysis. |
| Demarteau et al.,  2010,  France, Ireland, Italy [29] | QALY; cervical cancer cases and genital warts averted also reported | Under base-case assumptions of 10-year sustained protection for both vaccines and no cross-protection, the quadrivalent vaccine dominated the bivalent vaccine in all three countries. Minimum cross-protection required for the bivalent vaccine to become cost-effective was 13% in Ireland, 15% in France, and 15% in Italy undiscounted; discounted values were 22% in France, 41% in Italy, and 44% in Ireland. | WHO-CHOICE GDP-based thresholds were used. One times GDP per capita was used for very cost-effective and three times GDP per capita was used for cost-effective, applied by country. | WHO-CHOICE GDP-based thresholds applied separately to each country. | The base case favored the 4-valent vaccine when no cross-protection was assumed. Conclusions were sensitive to assumed sustained protection and cross-protection of the bivalent vaccine. | Cross-protection of Vaccine A, sustained protection of Vaccine A, low-risk HPV incidence, cervical cancer incidence, genital wart costs, vaccine costs, and sustained protection of Vaccine B. A 50% reduction in low-risk HPV incidence lowered the required cross-protection the most. Vaccine cost had less of an impact, and 50-year sustained protection for Vaccine B prevented Vaccine A dominance under discounted conditions. | Product-comparison conclusions depended strongly on sustained-protection and cross-protection assumptions; in the base case without cross-protection, the quadrivalent vaccine dominated the bivalent vaccine, but sufficient sustained and cross-protection against oncogenic HPV could offset the economic value of genital wart prevention. | Industry-funded multi-jurisdictional head-to-head vaccine comparison. Cross-protection assumptions were important for interpretation. |
| Capri et al.,  2011,  Italy [48] | Costs averted — cost-consequence analysis; lesions averted also reported | Not applicable; no formal ICER calculated. Cost-consequence analysis estimated an additional €2,385,354 in annual costs averted for the bivalent vaccine versus the quadrivalent vaccine under lifetime efficacy, price parity, and 100% coverage; at 70% coverage, the estimate was €1,669,748. | Not applicable. This was a cost-consequence analysis. No ICER threshold was used. | Not applicable. Cost-consequence analysis. No ICER threshold was used. | No formal cost-effectiveness conclusion was reported. | Genital wart costs, cervical cancer costs, genital-wart treatment costs, vaccine-effectiveness differences, incident cases, and duration of sustained protection. Genital wart and cervical cancer costs were most influential in the one-way sensitivity analysis. A sustained-protection difference of 10 years or more in favor of the quadrivalent vaccine could reverse the conclusion. | The bivalent vaccine was projected to prevent more precancerous and cervical cancer lesions than the quadrivalent vaccine in Italy and to generate greater treatment-cost offsets; under lifetime efficacy, price parity, and 100% coverage, the model estimated approximately €2.39 million in additional annual costs averted. | Industry-funded Italian cost-consequence analysis using a vaccine price parity assumption. The findings are not directly comparable to formal cost-effectiveness analyses. |
| Praditsitthikorn et al., 2011,  Thailand [21] | QALY; LY also reported | The best strategy from the societal perspective was VIA every five years at ages 30–45 plus Pap smear every five years at ages 50–60, which dominated alternatives by being cost-saving with QALY gains. HPV vaccination at age 15 versus the current Thai Pap policy was estimated at Bt 181,000/QALY, above the Thai Bt 100,000/QALY threshold. HPV vaccination at age 25 versus VIA plus Pap smear was estimated at Bt 317,008/QALY. | The Thai national threshold of Bt 100,000 per QALY. | Country-specific Thai national threshold defined by national health technology assessment institutions. | Price dependent. Screening strategies were most cost-effective at prevailing prices, while HPV vaccination was cost-effective only with a substantial price reduction. | Duration of vaccine protection, vaccine price, discount rate, program coverage, and HPV transmission assumptions. Vaccine duration was the most influential parameter. Discount-rate changes had a greater impact on vaccination than on screening, with 0%, 3%, 5%, and 10% discount rates producing large ICER variation. | Improving VIA and Pap smear screening was identified as the most cost-effective cervical cancer prevention option in Thailand at prevailing vaccine prices; HPV vaccination was not attractive at current prices and would require a substantial price reduction, approximately to 25% of the current price, to become policy relevant. | Non-industry Thai HITAP/World Bank semi-Markov cost-utility analysis. The vaccine price threshold was central to the conclusion. |
| Schobert et al.,  2012,  Germany [27] | QALY; LYG also reported | Vaccination of 12–17-year-old girls versus screening only: €5,525/QALY and €10,205/LYG. | No fixed German threshold was available. The UK NICE threshold of £30,000 per QALY was used as an external benchmark. | External UK NICE benchmark, used because Germany had no fixed national cost-effectiveness threshold. | Cost-effective under the study assumptions. | Discount rate, duration of vaccine protection, utility scores, inclusion of the HPV 6 and 11 component, and vaccination coverage. The discount rate was the most sensitive parameter. Excluding HPV 6 and 11 protections raised the ICER to €10,293/QALY, while coverage changes raised it to €5,807/QALY. | Quadrivalent HPV vaccination of girls aged 12–17 years was estimated to be cost-effective in Germany, with a base-case ICER of €5,525/QALY and €10,205/LYG. | Industry-funded German dynamic cost-effectiveness analysis. The ICER was positive at €5,525/QALY, so the intervention was cost-effective but not cost-saving or dominant. |
| Termrungruanglert et al., 2012,  Thailand [39] | QALY | Quadrivalent vaccination versus current practice: Bt 160,649.50/QALY. Sensitivity analyses ranged from Bt 51,688 to Bt 1,005,734/QALY across parameter variations. | WHO-CHOICE GDP-based threshold. Thai 2009 GNI per capita was US$3,869, equivalent to Bt 135,415 for one times GNI per capita. | WHO-CHOICE GDP-based threshold using Thai gross national income per capita. | Cost-effective under the broader WHO-CHOICE threshold, but not very cost-effective under the threshold based on one times GDP per capita, because the base-case ICER exceeded that stricter threshold. | Vaccine price, vaccine coverage, utility value for genital warts, vaccine efficacy, and HPV attribution to disease. Doubling the vaccine price exceeded the threshold. Coverage below 80% exceeded the threshold. Utility values for genital warts produced a wide ICER range from Bt 51,688 to Bt 1,005,734/QALY. | Quadrivalent HPV vaccination was estimated to be cost-effective in Thailand under the broader WHO-CHOICE threshold, with a base-case ICER of Bt 160,649.50/QALY; the conclusion was sensitive to vaccine price, vaccine coverage, utility values, and vaccine efficacy. | Industry-funded Thai TreeAge cost-effectiveness analysis with conclusions that contrast with Praditsitthikorn 2011. |
| Aponte-González et al., 2013,  Colombia [22] | DALY | Quadrivalent vaccination versus no vaccination: US$24,241/DALY averted. Bivalent vaccination versus quadrivalent vaccination: US$28,765/DALY averted. Both exceeded the 3×GDP per capita threshold of US$18,673 and were classified as not cost-effective under WHO-CHOICE. | WHO-CHOICE GDP-based threshold. Colombia's 2010 GDP per capita was US$6,224. The threshold based on three times GDP per capita was US$18,673. | WHO-CHOICE GDP-based threshold using Colombian GDP per capita. | Not cost-effective at prevailing vaccine prices. | Vaccine price, vaccine efficacy, cross-protection, discount rate, LSIL and HSIL progression risk, Pap sensitivity, and screening coverage. The vaccine price was the most influential factor, with a 32% to 36% reduction needed for cost-effectiveness. Including cross-protection made both vaccines cost-effective. A 0% discount rate made both vaccines very cost-effective. Lower Pap sensitivity favored vaccination, and vaccination became cost-effective when screening coverage fell to 0% to 20%. | HPV vaccination was not cost-effective at prevailing prices in Colombia, with ICERs exceeding the 3×GDP per capita threshold; threshold analyses indicated that vaccine prices would need to fall by approximately 32–36% for vaccination to become cost-effective in the local context. | Non-industry Colombian cost-effectiveness analysis with an explicit vaccine price threshold framework. |
| Berkhof et al.,  2013,  CEECA region; Slovenia, Poland, Georgia [40] | QALY; LY and lifetime cervical cancer risk reduction also reported | In the 28-country analysis, vaccination at I$100/girl was very cost-effective in 25 of 28 countries; Tajikistan, Turkmenistan, and Uzbekistan were exceptions. At I$200/girl, vaccination was very cost-effective in 20 of 28 countries. In the three-country detailed analysis, vaccination plus 10-year HPV screening was estimated at I$8,409–17,151/QALY in Slovenia, I$7,544–15,533/QALY in Poland, and I$8,346–10,697/QALY in Georgia. | WHO-CHOICE GDP-based thresholds were used. One times GDP per capita was used for very cost-effective, and three times GDP per capita was used for cost-effective, applied by country. Examples were Slovenia I$25,053, Poland I$17,348, and Georgia I$4,546. | WHO-CHOICE GDP-based thresholds applied using country-specific GDP per capita values. | Cost-effective under the study assumptions. | Resource constraints, CIN3-to-cancer duration, screening coverage, cytology sensitivity for CIN3, HPV incidence, and cross-protection. GDP per capita explained 56% of the variation in vaccination cost thresholds across 28 countries. CIN3-to-cancer duration was a highly uncertain natural-history parameter. Lower screening coverage favored more intensive screening, and 50% cytology sensitivity favored HPV testing. | Combined HPV16/18 vaccination and HPV screening was projected to be very cost-effective in countries with sufficient resources, while low vaccine pricing was essential for cost-effectiveness in lower-resource CEECA settings. | Non-industry CEECA 28-country regional two-tier modeling framework. |
| Bresse et al.,  2013,  France [50] | Cases averted and disease cost savings — cost-consequence analysis; no QALY/DALY | Not applicable; no formal ICER calculated. Cost-consequence analysis reported incremental cost savings and cases averted; vaccine acquisition costs were excluded. | Not applicable. This was a cost-consequence analysis. No ICER threshold was used. | Not applicable. Cost-consequence analysis. No ICER threshold was used. | No formal cost-effectiveness conclusion was reported. | Discount rate, treatment costs, and cross-protection assumptions. Discount rates were the most influential parameter, followed by treatment costs. Cross-protection assumptions had a limited impact on the conclusion, with the quadrivalent vaccine favored across scenarios A–H. | Quadrivalent vaccination was associated with greater projected economic benefits than bivalent vaccination in France, mainly because prevention of genital warts generated €306–380 million in healthcare cost savings over 100 years, while the marginal cost savings from cross-protection against cervical cancer were smaller. | Industry-funded French cost-consequence analysis excluding vaccine acquisition costs. The findings are not directly comparable to formal cost-effectiveness analyses. |
| Kim et al.,  2013a,  Sub-Saharan Africa 48 countries; Uganda, South Africa [23] | DALY; YLS also reported for Uganda and South Africa microsimulation analyses | In the 48-country analysis, vaccination at I$5/girl was cost-saving in 38 of 48 countries, with remaining countries at ≤I$300/DALY averted. At I$25/girl, vaccination was cost-effective in nearly all sub-Saharan African countries. In Uganda, vaccination alone at I$5/girl was cost-saving, and vaccination plus HPV DNA testing three times per lifetime was I$300/YLS. In South Africa, vaccination alone at I$10/girl was cost-saving, and vaccination plus HPV DNA testing three times per lifetime was I$1,600/YLS. | WHO-CHOICE GDP-based thresholds were used. One times GDP per capita was used for very cost-effective and three times GDP per capita was used for cost-effective, applied by country. Examples were Uganda I$1,141 and South Africa I$9,476. | WHO-CHOICE GDP-based thresholds applied using country-specific GDP per capita values. | Cost-effective under the study assumptions. | Vaccine cost, discount rate, cancer incidence, cancer cost, and mortality-to-incidence ratio. Vaccine cost was most influential, with a cost-effectiveness inflection point around I$25 to I$50 per vaccinated girl. A 0% discount rate made vaccination more attractive, while a 6% rate made it less attractive. Cancer incidence changes of ±50% had moderate impact, while cancer cost and mortality-to-incidence ratio had smaller effects. | HPV vaccination was projected to be cost-effective across much of sub-Saharan Africa, especially at low vaccine prices; at I$5 per vaccinated girl, vaccination was cost-saving in 38 of 48 countries, and at I$25 per vaccinated girl it remained cost-effective in nearly all countries. | Gates Foundation 48-country sub-Saharan Africa regional two-tier framework with macro-extrapolation. |
| Kim et al.,  2013b,  EMENA region 20 countries; Algeria,  Lebanon, Turkey [24] | DALY; YLS also reported for Algeria, Lebanon, and Turkey microsimulation analyses | In the 20-country analysis, vaccination at I$5/girl was cost-saving in 8 of 20 countries and cost-effective in the remainder. At I$25/girl, vaccination was cost-effective in 15 of 20 countries; it was not cost-effective in Egypt, Iran, Iraq, Saudi Arabia, and Syria. At I$200/girl, vaccination was cost-effective only in Pakistan, Morocco, Algeria, Bahrain, and Kuwait. | WHO-CHOICE GDP-based threshold was used. One times GDP per capita was used for very cost-effective, applied by country. Examples were Algeria I$7,521, Lebanon I$12,605, and Turkey I$12,540. | WHO-CHOICE GDP-based thresholds applied using country-specific GDP per capita values. | Cost-effective under the study assumptions. | Vaccine cost, discount rate, cancer incidence, HPV 16 and 18 type distribution, cancer cost, and mortality-to-incidence ratio. Vaccine cost was most influential, with strong threshold effects. A 0% discount rate made vaccination more attractive, while a 6% rate made it less attractive. Cancer incidence changes of ±50% had a moderate impact, including a Pakistan registry versus Globocan scenario, changing the threshold from I$100 to I$50 per girl. HPV 16 and 18 type distribution in Pakistan had minimal impact, and cancer cost and mortality-to-incidence ratio had modest effects. | Pre-adolescent HPV vaccination was projected to offer good value for money in most EMENA countries at low vaccine costs; at I$25 per vaccinated girl, it was cost-effective in 15 of 20 countries, but affordability, feasibility, acceptability, and screening-program organization remained important policy considerations. | Gates Foundation 20-country EMENA regional two-tier framework. |
| Jit et al.,  2014,  England and Canada [7] | Cancer cases prevented and number needed to vaccinate; no QALY/DALY | Not applicable; no formal ICER calculated. Clinical impact analysis reported cancer cases prevented, and the number needed to vaccinate. | Not applicable. This was a clinical impact analysis. No economic threshold was used. | Not applicable. Clinical impact analysis. No economic threshold was used. | No formal cost-effectiveness conclusion was reported. | Duration of two-dose protection, coverage, cross-protection, and vaccine waning function. Duration of two-dose protection was the most influential parameter, with an important threshold around 20 years. Coverage assumptions (40% versus 80%), inclusion of cross-protection, and fixed versus exponential waning also affected results. | Two-dose schedules were projected to provide most of the cancer-prevention benefits of a three-dose schedule if protection lasted at least 20 years; if two-dose protection lasted only 10 years, the third dose could prevent as many cancers as the first two doses. | UK and Canada number-needed-to-vaccinate clinical impact analysis. This was not a formal cost-effectiveness analysis. |
| Bonanni et al.,  2015,  Italy [49] | Cervical cancer cases prevented; no QALY/DALY | Not applicable; no formal ICER calculated. Cases-prevented analysis reported population impact only. | Not applicable. This was a case-prevention analysis. No economic threshold was used. | Not applicable. Cases prevented analysis. No economic threshold was used. | No formal cost-effectiveness conclusion was reported. | Vaccination coverage, mismatch in vaccination between boys and girls, duration of vaccine protection, and vaccine efficacy. Coverage in the catch-up female cohort and primary 11-year-old girls was most influential. A 0% mismatch provided no additional cervical cancer prevention for boys, while a 100% mismatch provided the maximum benefit. Protection duration of 10, 20, 30 years, or lifetime affected results, while vaccine efficacy changes had less impact. | With a fixed additional budget, extending vaccination to girls or young women was estimated to prevent more cervical cancer cases than vaccinating boys; boys' vaccination became more favorable only under scenarios with low vaccination coverage in the primary female target group. | Industry-funded Italian cases-prevented model. This was not a formal cost-effectiveness analysis. |
| Aljunid et al.,  2016,  Malaysia [41] | QALY | Two-dose vaccination versus three-dose vaccination produced the same number of cervical cancer cases averted, deaths averted, and QALYs, but saved RM 36,722,700, approximately US$9 million in 2013, over the lifetime horizon. If the savings were used to vaccinate an additional 137,025 girls, a further 180 cervical cancer cases and 63 deaths could be avoided. | WHO-CHOICE GDP-based threshold was referenced, but the explicit threshold value was not reported. | WHO-CHOICE GDP-based threshold using Malaysian GDP per capita. | Cost-saving versus the three-dose schedule. Interpretation depended on assumed dose non-inferiority and price or dose assumptions. | Not reported. The analysis was single deterministic. | A two-dose HPV vaccination schedule was projected to achieve similar cervical cancer outcomes to a three-dose schedule while saving approximately RM 36.7 million over the lifetime horizon; interpretation depends on assumptions about dose non-inferiority, price parity, full coverage, and the exclusion of herd immunity. | Industry-funded Malaysian two-dose versus three-dose cost-effectiveness analysis. Interpretation depends on assumed dose non-inferiority and vaccine price assumptions. |
| Setiawan et al.,  2016a,  Indonesia [42] | QALY | VIA screening alone versus no intervention: I$3,126/QALY. VIA plus HPV vaccination versus no intervention: I$1,863/QALY. Both were below Indonesia's per-capita GDP threshold of I$3,475 and were therefore classified as very cost-effective under WHO-CHOICE. | WHO-CHOICE GDP-based thresholds were used. One times GDP per capita was used for very cost-effective classification, and three times GDP per capita was used for cost-effective. Indonesia 2013 GDP per capita was I$3,475. | WHO-CHOICE GDP-based thresholds applied using Indonesian GDP per capita. | Cost-effective under the study assumptions. | Discount rate, cervical cancer treatment costs, QALY utilities, cryotherapy coverage, VIA detection rate, and vaccine price. Discount rates of 1.5%, 3%, and 5% were tested. Cervical cancer treatment costs varied by ±50%. Cervical cancer utility ranged from 0.48 to 0.84. Cryotherapy coverage ranged from 13% to 100%, and VIA detection rate ranged from 2.5% to 12.8%. The vaccine price was the most influential factor in the scenario analysis. | Adding HPV vaccination to VIA screening was projected to be very cost-effective in Indonesia, with VIA plus vaccination estimated at I$1,863/QALY compared with no intervention; strengthening the screening program remained important. | Indonesian DIKTI-funded cost-effectiveness analysis conducted under lower vaccine price assumptions. |
| Setiawan et al.,  2016b,  Indonesia [54] | Net present value — fiscal outcome; no QALY/DALY | Not applicable; no formal ICER calculated. Fiscal net present value analysis reported net present value per strategy. | Fiscal benchmark. Positive net present value or positive return on investment from the Indonesian government perspective. No ICER threshold was used. | Fiscal benchmark based on positive net present value or positive return on investment from the Indonesian government perspective. No ICER threshold was used. | Fiscally favorable from the government perspective. No formal ICER-based cost-effectiveness conclusion was reported. | Inflation rate, discount rate, GDP as an earnings proxy, tax compliance, health and social security membership, earnings consumed, and work-related accident premium. The 6.42% inflation rate and the 3% discount rate were the most influential. GDP and tax compliance were the next most influential, while health and social security membership, earnings consumed, and work-related accident premiums were less influential. | Investment in HPV vaccination and cervical screening was projected to generate fiscal benefits for the Indonesian government through reduced cervical cancer morbidity and mortality, with epidemiological gains translating into long-term health-economic and fiscal benefits. | Indonesian fiscal net present value analysis using a government tax-revenue framework. This was not a formal cost-effectiveness analysis. |
| Vargas Parada and Veerman, 2016,  Chile [33] | DALY | Base-case ICER: US$138,269/DALY, with 95% uncertainty interval $95,936–$221,862. This exceeded the 3×GDP per capita threshold of US$55,758/DALY and was classified as not cost-effective. | Chilean GDP-based benchmark. One times GDP per capita was US$18,586 for very cost-effective. Three times GDP per capita was US$55,758 for cost-effective. | Chilean Economic Evaluation Guidelines using GDP-based thresholds. Chilean health authorities generally use one time GDP per capita as the acceptable threshold. | Not cost-effective at the base-case vaccine price. Potentially cost-effective only with a substantial vaccine price reduction. | Vaccine price, discount rate, target population, and vaccine efficacy. The vaccine price was the most influential factor, with threshold analysis indicating the need for a price reduction. A 0% discount rate produced an ICER of US$44,411 and was cost-effective, while a 6% discount rate increased the ICER to US$346,719. A younger HIV-positive male target population aged 15 to 25 years reduced the ICER to US$123,507 compared with the base-case US$138,269. Vaccine efficacy had modest impact, comparing per-protocol efficacy of 77.5% with intention-to-treat efficacy of 50.3%. | HPV vaccination for HIV-positive men was not cost-effective in Chile at the base-case price, with an ICER of US$138,269/DALY compared with a 3×GDP per capita threshold of US$55,758/DALY; substantial vaccine-price reductions would be required for the strategy to become cost-effective. | Chilean academic multistate Markov analysis with DisMod II adaptation. |
| Kosen et al.,  2017,  Indonesia [43] | QALY | Without catch-up vaccination versus screening only: US$450/QALY. Catch-up vaccination versus no catch-up vaccination: US$390/QALY. Both were well below Indonesia's 2014 1×GDP per capita threshold of US$3,531.80 and were classified as very cost-effective under WHO-CHOICE. | WHO-CHOICE GDP-based threshold. Indonesia 2014 GDP per capita was US$3,531.80 for very cost-effective. | WHO-CHOICE GDP-based threshold using Indonesian GDP per capita. | Cost-effective under the study assumptions. | Not reported in detail. Extensive one-way and multivariate sensitivity analyses were reported, but the specific influential parameters were not detailed in the main paper. | Quadrivalent HPV vaccination of girls in Indonesia, with or without catch-up vaccination, was projected to substantially reduce genital warts, CIN, and cervical cancer, improve quality of life, and be very cost-effective under the applied GDP-based threshold, with ICERs of approximately US$450/QALY without catch-up and US$390/QALY for catch-up. | Industry-funded Indonesian cost-effectiveness analysis with a methodology that contrasts with that of Setiawan 2016a. |
| Mennini et al.,  2017, Italy [44] | QALY | Across six strategies, 9-valent girls-only vaccination versus 4-valent girls-only vaccination: €4,483/QALY. 9-valent universal vaccination versus 4-valent universal vaccination: €10,463/QALY. 9-valent universal vaccination versus 4-valent girls-only vaccination: €13,541/QALY. 9-valent girls-only vaccination versus screening only: €2,592/QALY. 9-valent universal vaccination versus screening only: €5,855/QALY. 9-valent girls-only vaccination dominated bivalent vaccination. | Italian pharmacoeconomic guideline threshold range of €25,000 to €40,000 per QALY. | Country-specific Italian pharmacoeconomic guideline threshold from the Italian Association of Health Economics. | Cost-effective under the study assumptions. | Discount rate, duration of protection, vaccine prices, including the low alternative price scenario, inclusion of recurrent respiratory papillomatosis, penile cancer, and head and neck cancers, utility values, lower coverage rates, and lower male adherence. The discount rate was influential: 6% increased ICERs substantially, whereas 0% or 1.5% outcomes-only discounting reduced ICERs. A 20-year protection duration resulted in worse outcomes than lifelong protection. Including recurrent respiratory papillomatosis, penile cancer, and head and neck cancers reduced the ICER for universal versus girls-only vaccination. Utility values, 60% coverage, and 85% male adherence were less influential. | Nine-valent HPV vaccination was projected to be cost-effective in Italy across girls-only and universal vaccination scenarios; 9v girls-only vaccination was estimated at €4,483/QALY versus 4v girls-only, and 9v universal vaccination at €10,463/QALY versus 4v universal vaccination, with additional benefits from prevention of HPV-related cancers and genital warts. | Industry-funded Italian SPMSD nine-valent dynamic cost-effectiveness analysis using the Elbasha framework lineage. |
| Chesson et al.,  2018,  United States [28] | QALY | The comparison scenario: females aged 12–26 and males aged 12–21 versus no vaccination: US$16,600/QALY. The expanded scenario, females and males aged 12–26, versus the comparison scenario: US$228,800/QALY, with a PSA 5th–95th percentile range of US$137,900–US$367,300 and a one-way sensitivity range of US$146,600–US$296,800. | No official ACIP or US government threshold was used. Results were interpreted against common US willingness-to-pay conventions, typically US$50,000 to US$150,000 per QALY. | No official ACIP or US government threshold was used. Results were interpreted against common US willingness-to-pay conventions. | Mixed and uncertain. The comparison scenario was cost-effective, but expanded male vaccination through age 26 years had a high ICER and did not make a strong economic case. | Vaccine cost, QALYs lost per outcome, HPV vaccine-type attribution, coverage scenarios, and medical cost per outcome. Vaccine cost changed the ICER from US$159,400/QALY at the lower bound to US$296,800/QALY at the upper bound. QALYs lost per outcome changed the ICER from US$146,600 to US$279,600. HPV vaccine-type attribution changed it from US$186,800 to US$275,100. Medical cost per outcome changed it from US$223,100 to US$235,000. | Expanding male HPV vaccination through age 26 years yielded high and uncertain ICERs; the expanded strategy had an estimated ICER of $228,800/QALY compared with the comparison strategy, which did not provide a strong economic case for expansion but did not rule out consideration. | CDC US dynamic transmission model with 5,000-sample probabilistic sensitivity analysis, evaluating expanded male and gender-neutral catch-up vaccination through age 26. |
| Yaghoubi et al.,  2018, Iran [32] | DALY | Government perspective ICER: US$15,205/DALY. Societal perspective ICER: US$14,999/DALY. Both exceeded the 3×GDP per capita threshold of US$14,289 and were classified as not cost-effective for the base-case three-dose program. | WHO-CHOICE GDP-based thresholds were used. One times GDP per capita was US$4,763 for very cost-effective. Three times GDP per capita was US$14,289 for cost-effective. | WHO-CHOICE GDP-based thresholds, with reference to published discussions of limitations in GDP-based thresholds. | Not cost-effective at the base-case three-dose price. A two-dose schedule or a reduction in vaccine price could change the conclusion. | Vaccine price, number of doses, coverage, vaccine efficacy, cervical cancer treatment costs, HPV 16 and 18 attributions, and access to treatment. The vaccine price was most influential, with a base-case price of US$13.75, a lower bound of US$12.37, and a threshold around US$4 per dose for high cost-effectiveness. A two-dose schedule, coverage, vaccine efficacy, treatment costs, HPV attribution, and treatment access also affected results. | A three-dose HPV vaccination program was not cost-effective in Iran at base-case prices, with ICERs of US$15,205/DALY from the government perspective and US$14,999/DALY from the societal perspective, both above the 3×GDP per capita threshold of US$14,289/DALY; lower prices or a two-dose schedule could change the conclusion. | WHO-funded Iranian CERVIVAC standardized LMIC framework |
| Bahr et al.,  2019,  Lebanon [53] | Cost-benefit ratio — simplified cost-benefit analysis; no QALY/DALY | Not applicable; no formal ICER calculated. Simplified cost-benefit analysis reported a cost ratio of 3.4:1 against vaccination. | Cost-benefit benchmark. A cost ratio of one-to-one was used as the break-even point. No ICER threshold was used. | Cost-benefit benchmark based on treasury cost ratio interpretation. The break-even point was a cost ratio of one-to-one. | Not cost-beneficial under the study assumptions. | Vaccine price, cervical cancer incidence, HPV prevalence, and cervical cancer management cost growth. Vaccine price was the primary driver, with the break-even price decreasing from US$71 to US$20.88 per dose. Other influential factors were low cervical cancer incidence at 5.3 per 100,000, low HPV prevalence at 4.9%, and 15.6% annual growth in cervical cancer management costs. | Mass HPV vaccination was not cost-beneficial in Lebanon under the 2016 assumptions, with a cost-benefit ratio of 3.4:1 against vaccination; the authors suggested reassessment if vaccine prices, cervical cancer incidence, or cost conditions changed. | Lebanese cost-benefit analysis using a simplified cost-ratio framework. This was not a cost-effectiveness or cost-utility analysis. Vaccine acquisition costs were included, but administrative/operational vaccination costs and indirect cancer costs were excluded. The conclusion applies only under the stated cost-ratio assumptions. |
| Datta et al.,  2019,  United Kingdom [10] | QALY | Threshold dose-price analysis rather than traditional ICER reporting. Girls-only vaccination versus halted vaccination at 3.5% discounting: bivalent £55.80, quadrivalent £99.64, and nonavalent £108.05 per dose. Gender-neutral vaccination versus halted vaccination at 3.5% discounting: bivalent £25.08, quadrivalent £48.38, and nonavalent £52.77 per dose. Gender-neutral versus girls-only vaccination was not favorable at 3.5% discounting, with negative threshold dose prices from -£5.67 to -£2.56, but became favorable at 1.5% discounting, with threshold prices from £36.46 to £46.88. | UK NICE and JCVI benchmark. £20,000 per QALY was used as the base threshold, and £30,000 per QALY was used as the upper threshold. | UK NICE Reference Case and JCVI 2018 guideline thresholds. | Girls-only vaccination was highly cost-effective versus no vaccination. Gender-neutral vaccination was cost-effective only at 1.5% discounting and was not favorable at the UK base-case discount rate of 3.5%. | Uptake rate, discount rate, vaccine price, vaccine type, cross-protection assumptions, and prior vaccination context. Lower uptake made adding boys more cost-effective, while high UK uptake reduced incremental value through herd immunity. Discounting at 3.5% versus 1.5% strongly affected results. Vaccine type ranked nonavalent, quadrivalent, then bivalent. Vaccination-naïve scenarios made adding boys more cost-effective. | Girls-only HPV vaccination was highly cost-effective compared with no vaccination, while adding boys to an already successful girls-only program was less favorable and depended strongly on discounting; gender-neutral vaccination became more favorable under a 1.5% outcome discount rate. | UK Department of Health individual-based MCMC modeling using NATSAL behavioral data and threshold dose-price methodology. |
| Lee et al.,  2019,  South Korea [30] | QALY; LY also reported | At the Korean 5% base-case discount rate, AS04 vaccination versus screening alone: KRW 23.9 million/QALY. AS04 vaccination versus quadrivalent vaccination produced a negative ICER of -KRW 58.4 million/QALY, reflecting cost savings of KRW 291.9 million with five fewer QALYs. At 3% discounting, AS04 vaccination versus screening alone was KRW 7.3 million/QALY, and AS04 dominated quadrivalent vaccination. At 1.5% discounting, AS04 vaccination versus screening alone was KRW 2.1 million/QALY, and AS04 again dominated quadrivalent vaccination. | Korean implicit willingness to pay threshold of KRW 20 million to KRW 30 million per QALY. | Country-specific Korean implicit threshold from NECA. | Cost-effective versus screening at all discount rates. Dominant versus quadrivalent vaccination at 3% and 1.5% discounting. The negative ICER with fewer QALYs at the 5% base-case discount rate requires cautious interpretation. | Discount rate, HPV 16 and 18 distribution in cervical cancer, vaccine cost, screening parameters, HPV distribution in cervical cancer and genital warts, screening participation, HPV incidence, and duration of cross-protection efficacy. The discount rate was most influential because of the timing difference between long-term cervical cancer benefits and short-term genital wart benefits. | Two-dose AS04-HPV16/18 vaccination was cost-effective compared with screening alone in Korea, with an ICER of KRW 23.9 million/QALY at the 5% base-case discount rate; compared with quadrivalent vaccination, AS04-HPV16/18 was projected to provide comparable or better outcomes at lower cost under the model assumptions. | Industry-funded Korean head-to-head comparison using the Demarteau methodology. |
| Messoudi et al.,  2019,  Morocco [45] | LYS; no QALY/DALY | Vaccination alone at 70% coverage for 14-year-old girls: US$1,150/LYS. VIA every three years at 10% coverage: US$551/LYS. Combined VIA every three years at 10% coverage plus vaccination at 70% coverage: US$2,843/LYS. VIA every three years at 70% coverage plus vaccination at 70% coverage: US$14,170/LYS and not cost-effective above 50% VIA coverage. VIA once in a lifetime dominated higher VIA frequencies. | WHO-CHOICE GDP-based thresholds were used. One times GDP per capita was US$2,860 for highly cost-effective. Three times GDP per capita was US$8,580 for cost-effective. | WHO-CHOICE GDP-based thresholds, with acknowledgement of published discussions of limitations in GDP-based thresholds. | Cost-effective under the study assumptions. | VIA coverage, vaccine cost per dose, vaccination coverage, VIA sensitivity, and VIA cost. VIA coverage was most influential, ranging from 5% to 70% and producing ICERs from US$997 to US$5,702/LYS. Vaccine cost per dose ranged from US$5 to US$35. VIA sensitivity ranged from 30% to 80%. | HPV vaccination and VIA screening were projected to be cost-effective cervical cancer prevention options in Morocco; vaccination alone was estimated at US$1,150/LYS, VIA every three years at US$551/LYS, and combined VIA plus vaccination at US$2,843/LYS under base-case coverage assumptions. | Spanish ICO-funded Moroccan static Markov analysis using life-years saved as the main outcome. |
| Smith et al.,  2019,  Canada [55] | Cervical cancer cases and costs — coverage-uptake impact analysis; no QALY/DALY | Not applicable; no formal ICER calculated. Coverage-uptake impact analysis reported cancer cases averted and cost differences only. Increasing uptake from 0% to 67% produced estimated lifetime cost savings of US$160 million. Increasing uptake from 67% to 90% produced US$9 million in additional costs, approximately a 1% increase. | Not applicable. This was a coverage uptake impact analysis. No formal ICER threshold was used. | Not applicable. Coverage uptake impact analysis. No formal ICER threshold was used. | No formal cost-effectiveness conclusion was reported. | Vaccination coverage uptake, jurisdictional variation, transition to 9-valent vaccine, boys' inclusion, partial vaccine-course protection, and marketing or promotional expenditures. Coverage uptake was the primary variable analyzed. Jurisdictional uptake ranged from 55% in the Northwest Territories to 92% in Newfoundland. The 9-valent transition, boys' inclusion, and partial course protection were acknowledged, and marketing or promotional expenditures were not included. | Increasing HPV vaccination uptake in Canada was projected to avert additional cervical cancer cases and generate long-term economic benefits; moving from 0% to 67% uptake produced estimated lifetime cost savings of about US$160 million, while increasing uptake from 67% to 90% produced additional health gains at modest additional cost. | Canadian OncoSim coverage-uptake impact analysis. This was not a formal cost-effectiveness analysis. |
| Tang et al.,  2019,  Taiwan [31] | QALY; LY also reported | At 3% discounting, AS04 vaccination versus screening alone: NT$158,157/QALY. AS04 vaccination dominated two-dose quadrivalent vaccination, with 0.0201 additional QALYs and NT$293 cost savings. AS04 vaccination dominated two-dose nonavalent vaccination, with 0.0049 additional QALYs and NT$2,687 cost savings. AS04 vaccination dominated three-dose nonavalent vaccination, with 0.0049 additional QALYs and NT$7,226 cost savings. | WHO-CHOICE GDP-based threshold. One times GDP per capita was NT$727,818 per QALY. | WHO-CHOICE GDP-based threshold using Taiwanese GDP per capita. | Dominant versus two-dose quadrivalent, two-dose nonavalent, and three-dose nonavalent vaccination strategies. Cost-effective versus screening alone. | Vaccine cost, vaccine effectiveness, discount rate, HPV prevalence, and treatment costs. Vaccine effectiveness, especially against CIN3 and cervical cancer, was important. AS04 dominance over 9-valent vaccination reversed only when lower-limit AS04 effectiveness and upper-limit 9-valent effectiveness were assumed simultaneously. | Two-dose AS04-HPV16/18 vaccination alongside screening was projected to be cost-effective compared with screening alone, with an ICER of NT$158,157/QALY, and dominant compared with two-dose quadrivalent and two- or three-dose nonavalent vaccine strategies in the Taiwanese model. | Industry-funded Taiwan head-to-head four-way vaccine comparison using the Demarteau framework and probabilistic sensitivity analysis. |
| Chauhan et al.,  2020,  India [46] | QALY; LY also reported | At 3% discounting, VIA every five years versus no screening: US$829/QALY and was the recommended strategy. VIA every 10 years versus no screening: US$564/QALY. VIA every three years versus no screening: US$2,310/QALY and was extended dominated. HPV DNA every three years versus no screening: US$20,151/QALY and was not cost-effective. Pap smear strategies were dominated. HPV vaccination alone versus no vaccination/no screening: US$86/QALY. HPV vaccination plus VIA every 10 years: US$402/QALY. HPV vaccination plus VIA every five years: US$476/QALY. | WHO-CHOICE GDP-based thresholds were used. One times GDP per capita was US$1,890 for highly cost-effective. Three times GDP per capita was US$5,670 for cost-effective. | WHO-CHOICE GDP-based threshold using Indian GDP per capita. | Cost-effective under the study assumptions. | Treatment coverage, lifetime cervical cancer risk, VIA sensitivity, HPV vaccination coverage, discount rate, vaccine effectiveness, vaccine cost, and treatment costs. VIA requires treatment coverage above 30%, lifetime cervical cancer risk above 0.70%, and VIA sensitivity above 17% to remain cost-effective every five years. A 50% HPV vaccination coverage threshold affected the combined strategy comparison. | VIA screening every five years was recommended as the most cost-effective screening strategy in India, with an ICER of US$829/QALY; HPV vaccination alone was very cost-effective at US$86/QALY, and future screening frequency should depend on vaccination coverage. | Indian Department of Health Research comprehensive nine-strategy screening and HPV vaccination cost-effectiveness analysis with probabilistic sensitivity analysis. |
| Kitano,  2022,  Japan [56] | QALY; risk-benefit ratio also reported | The primary outcome was a risk-benefit ratio of 0.022, corresponding to a benefit-to-risk ratio of 44.670. Secondary ICERs were JPY 12,660,298/QALY for a three-dose program and JPY 8,179,493/QALY for a two-dose program. Without discounting, ICERs were JPY 10,045,252/QALY for three doses and JPY 5,564,447/QALY for two doses. Sensitivity analyses showed a risk-benefit ratio range of 0.0001–0.11, with benefits consistently exceeding risks. | No formal threshold was cited. ICER was reported as secondary to the risk-benefit analysis. | No formal threshold was cited. The primary decision framework was risk-benefit analysis, with ICER reported as a secondary result. | No formal cost-effectiveness conclusion was primary. The main decision framework was risk-benefit analysis. | Chronic adverse reaction incidence, HPV infection burden, duration of protection, vaccine efficacy, and discount rate. Chronic adverse reaction causality ranged from 0% to 100%. HPV infection burden ranged from 80% lower to 10% higher than the base case. Duration of protection ranged from until age 30 to lifelong. Vaccine efficacy ranged from 90.4% to 100%. The discount rate ranged from 0% to 6%. Vaccine efficacy was the least sensitive parameter. | The risk-benefit analysis suggested that the health benefits of 9-valent HPV vaccination for adolescent boys in Japan outweighed potential vaccine-related risks; the benefit-to-risk ratio was 44.670, while secondary ICER estimates were JPY 12,660,298/QALY for a three-dose program and JPY 8,179,493/QALY for a two-dose program. | Single-author Japanese decision-tree risk-benefit analysis. This was not a formal cost-effectiveness analysis. |
| Owusu-Edusei et al.,  2022,  United Kingdom [25] | QALY | Universal nonavalent vaccination versus universal quadrivalent vaccination at 3.5% discounting: £8,600/QALY. At 1.5% discounting: £3,300/QALY. Sensitivity analyses ranged from £3,300 to £27,900/QALY. | UK NICE thresholds were used. £20,000 per QALY was used as the lower threshold, and £30,000 per QALY was used as the upper threshold. | UK NICE conventional thresholds for the National Health Service context. | Cost-effective under the study assumptions. | Disease utility, discount rate, vaccine efficacy, vaccine coverage, and duration of protection. Disease utility was most influential, raising the base-case ICER from £8,600 to £27,900 when increased by 20%. The discount rate changed the ICER from £3,300 at 1.5% to higher values at 5%. Vaccine efficacy was also influential. Vaccine coverage and duration of protection were less influential. | Switching from universal quadrivalent to universal nonavalent HPV vaccination in the UK was projected to be cost-effective, with an ICER of £8,600/QALY at 3.5% discounting and £3,300/QALY at 1.5% discounting, while preventing additional HPV-related disease across both sexes. | Industry-funded UK quadrivalent-to-nonavalent dynamic transmission cost-effectiveness analysis. |
| Sargazi et al.,  2022,  Iran [52] | Cost-benefit ratio and monetary net benefit; no QALY/DALY | The primary outcome was the cost-benefit ratio rather than the ICER. Bivalent vaccination had a cost-benefit ratio of 258.12 using the cost-of-illness approach and -15.11 using the willingness-to-pay approach. Quadrivalent vaccination had a cost-benefit ratio of 43.51 using the cost-of-illness approach and 2.51 using the willingness-to-pay approach. Net benefits per person were US$7,346 for bivalent vaccination using cost-of-illness, -US$460 for bivalent vaccination using willingness-to-pay, US$6,439 for quadrivalent vaccination using cost-of-illness, and US$229 for quadrivalent vaccination using willingness-to-pay. | Cost-benefit benchmark. Net benefit greater than zero or cost-benefit ratio greater than one. No formal ICER threshold was used. | Cost-benefit analysis criteria based on a positive net benefit or a cost-benefit ratio greater than one. | Cost-beneficial under the cost-benefit analysis framework. | Discount rate, vaccine cost, cervical cancer incidence, proportion of cervical cancer attributable to HPV, and vaccine effectiveness. Alternative discount rates of 5% and 7% had the largest effect on cost-benefit ratios: bivalent vaccine ratios under the cost-of-illness approach decreased from 258.12 to 131.08 and 71.36, respectively; quadrivalent ratios decreased from 43.51 to 22.10 and 12.03, respectively. | HPV vaccination showed favorable economic value in several cost-benefit scenarios in Iran, but conclusions varied by valuation method; the quadrivalent vaccine was cost-beneficial under both cost-of-illness and willingness-to-pay approaches, while the bivalent vaccine was favorable under the cost-of-illness approach but not under the willingness-to-pay approach. | Iranian TUMS cost-benefit analysis combining willingness-to-pay discrete choice experiment and cost-of-illness components. The results are not directly comparable to ICER-based cost-effectiveness analyses. |
| Palmer et al.,  2023,  Japan [11] | QALY | Gender-neutral vaccination versus female-only vaccination at 3% discounting: ¥4,732,320/QALY, equivalent to US$35,987/QALY. Sensitivity analyses ranged from ¥2,444,156 to ¥13,119,042/QALY. Scenario analyses showed that gender-neutral vaccination was cost-effective only at 30% primary female vaccination coverage, not at 50% or 70%. A 50-year time horizon increased the ICER to ¥10,184,538/QALY. | Japanese conventional willingness-to-pay threshold of ¥5 million per QALY, equivalent to US$38,023 per QALY. | Country-specific Japanese conventional willingness to pay threshold. | Cost-effective under the study assumptions. | Discount rate, disease disutility, survivor disutility, duration of vaccine protection, calibrated parameter combinations, utility among the healthy population, vaccine price, and treatment cost. The discount rate was most influential, with ICERs ranging from ¥2,444,156 to ¥13,119,042. Disease disutility changed the ICER from ¥2,487,399 to ¥11,575,375. Removing cancer survivor disutility changed the ICER from ¥4,732,329 to ¥7,352,291. A 20-year protection duration changed the ICER from ¥4,732,329 to ¥7,072,172. Ten calibrated parameter combinations produced ICERs from ¥4,282,133 to ¥5,069,389. Vaccine price and utility among healthy people had further impact, while treatment cost was the least influential. | Gender-neutral HPV vaccination was projected to avert additional HPV-related disease and deaths compared with female-only vaccination and was cost-effective in Japan at 30% female vaccine coverage, with a base-case ICER of ¥4,732,320/QALY against a ¥5 million/QALY threshold. | Industry-funded Japanese gender-neutral quadrivalent-to-nonavalent dynamic transmission analysis with Bayesian calibration. |
| Slavkovsky et al.,  2024,  Ethiopia, Guyana, Rwanda, Sri Lanka, and Uganda [51] | Vaccine delivery cost; no health outcome or ICER | Not applicable; no formal ICER reported. The study reported cost per dose, cost per adolescent receiving the full schedule, and national-level vaccine procurement costs. Single-dose schedules reduced financial cost per adolescent receiving the full schedule by 29% in Ethiopia, to 45% in Sri Lanka, and economic cost by 35% in Ethiopia, to 48% in Sri Lanka. Combined delivery and procurement costs per adolescent fell by approximately 40–50% in financial terms and 40–49% in economic terms across the reported settings. | Not applicable. This was a cost-of-delivery analysis. No ICER or cost-effectiveness threshold was used. | Not applicable. Cost- of-delivery analysis. No ICER or cost-effectiveness threshold was used. | Cost reduction for vaccine delivery only. No health outcome or ICER-based cost-effectiveness conclusion was reported. | Dose-based adjustment factors, session-based adjustment factors, frequency-based adjustment factors, quantity-based adjustment factors, and volume-based adjustment factors. Dose-based and session-based factors had the greatest impact on cost per dose and cost per adolescent under a single-dose schedule. Frequency-based factors, such as training and social mobilization, were influential where these activities represented a larger share of economic costs, for example, in Uganda. Quantity-based and volume-based adjustments had minimal impact. | Single-dose HPV vaccination schedules were projected to reduce program delivery and procurement costs compared with two-dose schedules across five LMIC settings; financial cost per fully vaccinated adolescent fell by approximately 29–45%, and economic cost fell by approximately 35–48%. | Gates Foundation five-LMIC cost-of-delivery study comparing one-dose and two-dose schedules. No health outcomes were assessed. |
| Şahin and Karabey,  2025,  Türkiye [26] | DALY | From the SGK perspective, Scenario 1 had an ICER of US$2,231/DALY and Scenario 2 had an ICER of US$2,342/DALY. The direct comparison between Scenario 2 and Scenario 1 yielded an incremental ICER of US$2,592/DALY. From the societal perspective, Scenario 1 had an ICER of US$2,175/DALY and Scenario 2 had an ICER of US$2,285/DALY. Both scenarios were classified as very cost-effective under WHO-CHOICE and Ochalek opportunity-cost thresholds. Threshold dose-price analysis showed Scenario 1 was highly cost-effective up to US$54 and not cost-effective above US$167, while Scenario 2 was highly cost-effective up to US$78 and not cost-effective above US$242. | WHO-CHOICE GDP-based thresholds were used. The threshold range was US$9,693 to US$29,079 per DALY averted. A supplementary opportunity cost threshold based on Ochalek et al. was also used. This was 0.30 to 0.60 times GDP per capita, equivalent to US$2,908 to US$5,816. | WHO-CHOICE GDP-based thresholds and supplementary opportunity cost-based thresholds from Ochalek et al. | Highly cost-effective under both WHO-CHOICE and opportunity-cost thresholds. | Discount rate, vaccine efficacy, HPV subtype distribution, vaccine price, DALY coefficients, and treatment costs. The discount rate from 1% to 5% was the most influential factor and produced the largest ICER changes. Vaccine efficacy and HPV subtype distribution were the second most impactful variables. Vaccine price reflected procurement and market dynamics. DALY coefficients and treatment costs had a lesser influence. | HPV vaccination was projected to be highly cost-effective in Türkiye, with ICERs of US$2,231/DALY for bivalent or quadrivalent vaccination and US$2,342/DALY for nonavalent vaccination from the SGK perspective, both below WHO-CHOICE and opportunity-cost thresholds. | Turkish PRIME standardized static cohort cost-effectiveness analysis using WHO-CHOICE and Ochalek opportunity-cost thresholds. The study addressed a previously unfilled Turkish evidence gap. |
| Termrungruanglert et al., 2024, Thailand [57] | QALY | Two-dose 9vHPV versus one-dose 9vHPV: 13,855.56 THB/QALY, equivalent to US$406/QALY. One-dose 9vHPV versus no vaccination was cost-saving, with an ICER of -6,101.48 THB/QALY. All vaccination strategies were cost-saving compared with no vaccination, with savings of 508.7–676.1 million THB (equivalent to US$14.9–19.2 million). Two-dose 9vHPV was the most effective strategy, producing 2,920,875.77 QALYs. | The Thai willingness to pay threshold of 160,000 THB per QALY gained, equivalent to 1.2 times gross national income per capita. | Country-specific Thai willingness-to-pay thresholds for pharmaceutical reimbursement decisions. | Cost-effective. Two-dose 9-valent HPV vaccination was the most cost-effective strategy below the Thai willingness-to-pay threshold. Single-dose and two-dose 9-valent strategies were cost-saving versus no vaccination. | Vaccine cost and vaccine efficacy were most influential. A 20% change in vaccine cost led to a 38% swing in the ICER. A 10% decrease in vaccine efficacy raised the ICER to 22,029 THB/QALY, a 59% increase. PSA showed an 80% probability that two-dose 9-valent HPV vaccination was cost-effective at a willingness-to-pay threshold of 160,000 THB/QALY. | Two-dose 9-valent HPV vaccination for 12-year-old girls, alongside primary HPV screening, was identified as the most cost-effective strategy in Thailand, with an ICER of 13,855.56 THB/QALY compared with one-dose 9vHPV, and cost savings with single-dose 9vHPV versus no vaccination. | First Thai study to include bivalent, quadrivalent, nine-valent, and single-dose vaccination strategies. QALY-based cost-utility analysis with primary HPV screening from a provider perspective. |
| Gao et al., 2025, Nigeria [47] | QALY | One-dose quadrivalent vaccination versus no vaccination: US$268.67/QALY from the health-system perspective, with 95% CI US$183.76–385.53, and US$217.85/QALY from the societal perspective, with 95% CI US$136.99–328.02. The program averted 924 cervical cancer cases and 287 deaths and gained 1,539 QALYs per 100,000 girls. | WHO-CHOICE GDP-based thresholds were used. One times GDP per capita was US$806.95 for very cost-effective. The range from one to three times GDP per capita, equivalent to US$806.95 to US$2,420.85, was used for cost-effective. | WHO-CHOICE GDP-based cost-effectiveness thresholds using Nigerian GDP per capita. | Very cost-effective from both health-system and societal perspectives. Results remained below the threshold across sensitivity analyses. | Vaccine cost, treatment success rate, cervical cancer treatment coverage, and cervical cancer utility. Vaccine cost was most influential. ICERs ranged from US$140 to US$296 when vaccine price varied from US$3 to US$6 per dose and increased to US$1,280 at US$25 per dose. Treatment success rate produced ICERs from US$184 to US$235 across probabilities of 0.82 to 0.96. | Single-dose quadrivalent HPV vaccination for girls aged 9–14 years was projected to be very cost-effective in Nigeria compared with no vaccination, with ICERs of US$268.67/QALY from the health-system perspective and US$217.85/QALY from the societal perspective; sustainable financing remains important as Gavi graduation approaches. | First economic evaluation of the single-dose HPV vaccination strategy adopted in Nigeria, using real-world program cost and coverage data. QALY-based cost-utility analysis from health-system and societal perspectives. |

***Classification key:*** *Full comparative economic evaluation, primary synthesis, n = 33. Contextual economic evidence records, contextual mapping only, n = 11. Contextual economic evidence records were included for descriptive mapping only and were not included in the primary methodological credibility or transferability synthesis.*

***Thresholds:*** *Decision thresholds and benchmarks are reported as described by the original publications. WHO-CHOICE refers to GDP-based cost-effectiveness benchmarks used by several included studies. Country-specific thresholds were reported where studies used national or jurisdiction-specific benchmarks.*

***Methodological and comparability notes:*** *These notes summarize information reported in the included articles, including model type, funding or institutional context where reported, analytic approach, and comparability considerations. They were used for descriptive mapping and should not be interpreted as a formal risk-of-bias assessment.*

***Abbreviations:*** *ACIP, Advisory Committee on Immunization Practices. AS04, adjuvant system 04. CBA, cost-benefit analysis. CEA, cost-effectiveness analysis. CI, confidence interval. CIN, cervical intraepithelial neoplasia. DALY, disability-adjusted life year. GDP, gross domestic product. GNI, gross national income. HITAP, Health Intervention and Technology Assessment Program. HPV, human papillomavirus. HTA, health technology assessment. ICER, incremental cost-effectiveness ratio. I$, international dollars. JCVI, Joint Committee on Vaccination and Immunisation. JPY, Japanese yen. KRW, Korean won. LMIC, low- and middle-income country or countries. LY, life year. LYG, life years gained. LYS, life years saved. NA, not applicable. NATSAL, National Survey of Sexual Attitudes and Lifestyles. NECA, National Evidence-based Healthcare Collaborating Agency. NICE, National Institute for Health and Care Excellence. NPV, net present value. QALY, quality-adjusted life year. RM, Malaysian ringgit. SGK, Social Security Institution. SPMSD, Sanofi Pasteur MSD. Bt/THB, Thai baht. TUMS, Tehran University of Medical Sciences. UK, United Kingdom. US, United States. USD, United States dollars. VIA, visual inspection with acetic acid. WHO-CHOICE, World Health Organization Choosing Interventions that are Cost-Effective. WTP, willingness to pay. YLS, years of life saved.*

***Row shading:*** *Green shading indicates full comparative economic evaluations included in the primary synthesis. Amber shading indicates contextual economic evidence records included for contextual mapping only. Row shading is used only as a visual aid; the classification key should be used as the authoritative interpretation if colors are not visible in print or journal formatting.*
