# Supplementary material for: Methodological drivers, reporting completeness, and transferability in HPV vaccination economic evaluations: A scoping review
Source: PLoS One. 2026 Oct 14;21(10):e0360732. doi: 10.1371/journal.pone.0360732 (PMC13667800; doi:10.1371/journal.pone.0360732)
Supplement: Supplementary file 4 [file pone.0360732.s004.docx]

**S4 Table. Per-publication summary of contextualization and transferability.**

| **Author(s), Year, Country** | **Contextualization rating** | **Main contextualization feature or transferability barrier** |
| --- | --- | --- |
| Goldie et al., 2004, United States [3] | High | Strong US target-context contextualization. External use would require reassessment of the societal perspective, screening practice, vaccine price, and threshold assumptions. |
| Elbasha et al., 2007, United States [6] | High | Strong US contextualization through dynamic transmission modeling. External adaptation would require inputs on local epidemiology, screening practices, vaccine prices, coverage, and thresholds. |
| Boot et al., 2007, The Netherlands [34] | High | Strong Dutch contextualization with friction-cost productivity approach. External adaptation would require reassessment of productivity costing conventions, screening practices, and threshold assumptions. |
| Kulasingam et al., 2008, United Kingdom [35] | High | Strong UK NHS contextualization. External adaptation would require reassessment of screening practices, payer perspectives, vaccine prices, and threshold assumptions. |
| Coupé et al., 2009, The Netherlands [36] | High | Strong Dutch contextualization through organized screening modeling and 14-type HPV genotype data. External adaptation would require local genotype distribution, screening practice, costs, and threshold inputs. |
| Kim et al., 2009, United States [9] | High | Strong US microsimulation with a clear age-specific value boundary for women aged 35 to 45 years. External adaptation would require local screening practice, prior exposure, vaccine price, and threshold assumptions. |
| Reynales-Shigematsu et al., 2009, Mexico [37] | Moderate | Moderate Mexican contextualization through adaptation of an existing framework. External adaptation would require additional local recalibration of costs, screening practice, and threshold assumptions. |
| Dasbach et al., 2010, Hungary [38] | High | Strong Hungarian contextualization through dynamic cost-effectiveness analysis. External adaptation would require inputs on local epidemiology, costs, screening practices, vaccine prices, and thresholds. |
| Demarteau et al., 2010, France, Ireland, Italy [29] | Moderate | Multi-jurisdictional product-comparison framework for France, Ireland, and Italy. Conclusions should be interpreted in relation to assumed cross-protection profiles and sustained-protection duration. |
| Praditsitthikorn et al., 2011, Thailand [21] | High | Strong Thai contextualization based on local epidemiology, costs, screening options, and the national threshold. Adaptation to other LMICs would require local recalibration. |
| Schobert et al., 2012, Germany [27] | High | Strong German SHI contextualization. External adaptation would require reassessment of the payer perspective, screening practices, vaccine prices, and threshold assumptions. |
| Termrungruanglert et al., 2012, Thailand [39] | Moderate | Moderate Thai contextualization using an industry-funded framework. Interpretation differs from Praditsitthikorn (2011) because the comparator choice, screening context, and price assumptions differ. |
| Aponte-González et al., 2013, Colombia [22] | High | Colombian price-threshold framework. Adaptation to other pre-introduction settings would require local inputs on vaccine prices, screening coverage, epidemiology, and thresholds. |
| Berkhof et al., 2013, CEECA region, detailed analysis of Slovenia, Poland, and Georgia [40] | Moderate | CEECA regional two-tier framework. Country-level estimates require local refinement. |
| Kim et al., 2013a, sub-Saharan Africa region, detailed analysis of Uganda and South Africa [23] | Moderate | Sub-Saharan Africa 48-country regional analysis with extrapolated cost data. Country-specific application would require local cost, epidemiology, and threshold inputs. |
| Kim et al., 2013b, EMENA region, detailed analysis of Algeria, Lebanon, and Turkey [24] | Moderate | EMENA 20-country regional analysis with extrapolated cost data. Country-specific application would require local cost, epidemiology, and threshold inputs. |
| Aljunid et al., 2016, Malaysia [41] | Moderate | Malaysian two-dose versus three-dose framework. External adaptation would require reassessment of price parity, coverage assumptions, and herd-immunity treatment. |
| Setiawan et al., 2016a, Indonesia [42] | High | Strong Indonesian contextualization through primary local cost data. External adaptation would require local epidemiological data, screening practices, and threshold inputs. |
| Vargas Parada and Veerman, 2016, Chile [33] | Moderate | Moderate Chilean contextualization. Cost proxies were used for anal cancer treatment. External adaptation would require disease-specific cost, epidemiology, and threshold inputs. |
| Kosen et al., 2017, Indonesia [43] | Moderate | Moderate Indonesian contextualization using dynamic transmission modeling. External adaptation would require stronger local cost inputs, screening assumptions, and threshold justification. |
| Mennini et al., 2017, Italy [44] | High | Strong Italian contextualization through nine-valent dynamic cost-effectiveness analysis with conservative no-cross-protection base case. External adaptation would require inputs on local epidemiology, screening practices, vaccine prices, and thresholds. |
| Chesson et al., 2018, United States [28] | High | Strong US contextualization and uncertainty analysis. External adaptation would require inputs on local transmission, coverage, screening, price, and threshold. |
| Yaghoubi et al., 2018, Iran [32] | Moderate | Iranian application of the CERVIVAC standardized LMIC framework. External adaptation to other LMICs would require local epidemiological, cost, and threshold data. |
| Datta et al., 2019, United Kingdom [10] | High | Strong UK contextualization using NATSAL behavioral data and individual-based transmission modeling. External adaptation would require local sexual behavior, screening, vaccine price, and threshold inputs. |
| Lee et al., 2019, South Korea [30] | Moderate | Korean product-comparison framework. Conclusions depend on the comparator vaccine, cross-protection assumptions, and discount rate. Interpretation requires careful attention to the context of the industry-funded head-to-head comparison between bivalent and quadrivalent vaccines. |
| Messoudi et al., 2019, Morocco [45] | Moderate | Moderate Moroccan contextualization through a static Markov model adapted from the Spanish ICO methodology. External adaptation would require local epidemiology, costs, screening, and threshold inputs. |
| Tang et al., 2019, Taiwan [31] | Moderate | Taiwanese product-comparison framework. Conclusions depend on vaccine-efficacy assumptions, broader efficacy irrespective of HPV type, methodology, and price assumptions. |
| Chauhan et al., 2020, India [46] | High | Strong Indian contextualization with local utility and screening strategy inputs. Adaptation to other LMICs would require recalibration of local costs, screening, epidemiology, and thresholds. |
| Owusu-Edusei et al., 2022, United Kingdom [25] | High | Strong UK contextualization through quadrivalent-to-nonavalent dynamic transmission modeling. External adaptation would require inputs on local epidemiology, screening practices, vaccine prices, and thresholds. |
| Palmer et al., 2023, Japan [11] | High | Strong Japanese contextualization through gender-neutral quadrivalent-to-nonavalent Bayesian metapopulation modeling. External adaptation would require inputs on local epidemiology, screening practices, vaccine prices, and thresholds. |
| Şahin and Karabey, 2025, Türkiye [26] | High | Turkish PRIME-based contextualization with WHO-CHOICE and opportunity-cost thresholds. Adaptation to other upper-middle-income countries would require local epidemiology, vaccine price, screening context, and threshold assumptions. |
| Termrungruanglert et al., 2024, Thailand [57] | High | Strong Thai contextualization through country-specific cervical disease utilities, costs, primary HPV screening context, and Thai willingness-to-pay threshold. External adaptation would require local schedule-specific vaccine effectiveness, duration of protection, vaccine price, screening context, and threshold assumptions. |
| Gao et al., 2025, Nigeria [47] | High | Strong Nigerian contextualization using real-world program cost and coverage data. External adaptation would require local cervical cancer epidemiology, treatment coverage, vaccine price, program financing context, and threshold assumptions. |

***Note:*** *This table summarizes target-context contextualization and one key transferability concern for each of the 33 full comparative economic evaluations. Ratings are based on the Welte-informed assessment described in the Methods. Contextualization to the original decision setting is distinct from cross-country transferability. A study may be highly contextualized to its own setting while still having limited transferability to another country. This table is intended as a per-publication summary and is not a full item-by-item transferability matrix. Methodological credibility is reported separately in the main text (Table 4).*

***Contextualization rating:*** *High indicates that several key target-context dimensions were substantially reflected, such as local epidemiology, cost data, demographics, or behavioral inputs, screening context, vaccine price, and/or locally relevant thresholds. Moderate indicates that some local inputs were used, but they were combined with key imported assumptions or regional extrapolations. Low indicates limited use of local data.*
