# Supplementary material for: Methodological drivers, reporting completeness, and transferability in HPV vaccination economic evaluations: A scoping review
Source: PLoS One. 2026 Oct 14;21(10):e0360732. doi: 10.1371/journal.pone.0360732 (PMC13667800; doi:10.1371/journal.pone.0360732)
Supplement: Supplementary file 5 [file pone.0360732.s005.docx]

**S5 Table. Item-level CHEERS 2022 reporting completeness matrix.**

| **Author(s), Year, Country** | **1. Title** | **2. Abstract** | **3. Background and Objectives** | **4. Health Economic Analysis Plan** | **5. Study Population** | **6. Setting and Location** | **7. Comparators** | **8. Perspective** | **9. Time Horizon** | **10. Discount Rate** | **11. Selection of Outcomes** | **12. Measurement of Outcomes** | **13. Valuation of Outcomes** | **14. Measurement and**  **Valuation of Resources/Costs** | **15. Currency, Price Date, Conversion** | **16. Rationale & Description of Model** | **17. Analytics and Assumptions** | **18. Characterizing Heterogeneity** | **19. Characterizing Distributional Effects** | **20. Characterizing Uncertainty** | **21. Approach to engagement with patients and others affected by the study** | **22. Study Parameters** | **23. Summary of Main Results** | **24. Effect of Uncertainty** | **25. Effect of engagement with patients and others affected by the study** | **26. Study findings, limitations, generalizability, and current knowledge** | **27. Source of Funding** | **28. Conflicts of Interest** | **Items Fully Reported (count)** | **Items Applicable (count, max 28)** | **% Fully Reported** | **Reviewer-defined reporting completeness band** |
| --- | --- | --- | --- | --- | --- | --- | --- | --- | --- | --- | --- | --- | --- | --- | --- | --- | --- | --- | --- | --- | --- | --- | --- | --- | --- | --- | --- | --- | --- | --- | --- | --- |
| Goldie et al., 2004, United States [3] | F | F | F | N | F | F | F | F | F | F | F | F | F | F | F | F | F | P | N | F | N | F | F | F | NA | F | F | F | 23 | 27 | 85.2% | High |
| Elbasha et al., 2007, United States [6] | P | F | F | N | F | F | F | P | F | F | F | F | P | F | F | F | F | P | N | F | N | F | F | F | NA | F | F | P | 19 | 27 | 70.4% | Moderate |
| Boot et al., 2007, The Netherlands [34] | F | F | F | N | F | F | P | F | F | F | P | P | P | P | P | P | P | N | N | P | N | P | F | P | NA | F | F | F | 12 | 27 | 44.4% | Low |
| French et al., 2007, Finland [60] | F | F | F | NA | F | F | F | NA | F | NA | F | F | NA | NA | NA | F | F | P | N | P | N | F | F | P | NA | F | F | F | 16 | 21 | 76.2% | Moderate |
| Kulasingam et al., 2008,  United Kingdom [35] | F | F | F | N | F | F | F | F | F | F | F | F | P | F | F | F | F | P | N | F | N | F | F | F | NA | F | F | F | 22 | 27 | 81.5% | High |
| Coupé et al., 2009,  The Netherlands [36] | F | F | F | N | F | F | F | P | F | F | F | F | P | F | F | F | F | P | N | F | N | F | F | F | NA | F | F | P | 20 | 27 | 74.1% | Moderate |
| Kim et al., 2009, United States [9] | F | F | F | N | F | F | F | F | F | F | F | F | P | F | F | F | F | P | N | F | N | F | F | F | NA | F | F | F | 22 | 27 | 81.5% | High |
| Reynales-Shigematsu et al., 2009, Mexico [37] | F | F | F | N | F | F | F | P | F | F | F | F | P | F | F | F | P | P | N | P | N | F | F | F | NA | F | F | F | 19 | 27 | 70.4% | Moderate |
| Dasbach EJ et al., 2010, Hungary [38] | F | F | F | N | F | F | F | F | F | F | F | F | P | F | F | P | P | P | N | F | N | P | F | F | NA | F | F | P | 18 | 27 | 66.7% | Moderate |
| Demarteau et al., 2010,  France; Ireland; Italy [29] | F | F | F | N | F | F | F | F | F | F | F | F | P | F | P | F | F | P | N | F | N | F | F | F | NA | F | F | P | 20 | 27 | 74.1% | Moderate |
| Capri et al., 2011, Italy [48] | F | F | F | NA | F | F | F | F | P | NA | F | F | NA | F | F | P | F | P | N | F | N | F | F | F | NA | F | F | F | 19 | 24 | 79.2% | Moderate |
| Praditsitthikorn et al., 2011, Thailand [21] | F | F | F | N | F | F | F | F | F | F | F | F | F | F | F | F | F | F | N | F | N | F | F | F | NA | F | F | F | 24 | 27 | 88.9% | High |
| Schobert et al., 2012, Germany [27] | F | F | F | N | F | F | P | F | F | F | F | F | P | F | P | F | F | P | N | P | N | P | F | F | NA | F | F | P | 17 | 27 | 63.0% | Moderate |
| Termrungruanglert et al., 2012, Thailand [39] | F | F | F | N | F | F | P | F | F | F | F | F | P | P | P | F | F | P | N | F | N | F | F | F | NA | F | F | F | 19 | 27 | 70.4% | Moderate |
| Aponte-González et al., 2013, Colombia [22] | F | F | F | N | F | F | F | F | F | F | F | F | F | F | F | F | F | F | N | F | N | F | F | F | NA | F | P | F | 23 | 27 | 85.2% | High |
| Berkhof et al., 2013,  CEECA region; Slovenia, Poland, Georgia [40] | F | F | F | N | F | F | F | F | F | F | F | F | P | P | F | F | F | F | N | F | N | F | F | F | NA | F | F | F | 22 | 27 | 81.5% | High |
| Bresse et al., 2013, France [50] | F | F | F | NA | F | F | P | F | F | F | P | F | NA | F | F | F | P | P | N | F | N | F | F | F | NA | F | F | P | 18 | 25 | 72.0% | Moderate |
| Kim et al., 2013a,  Sub-Saharan Africa  48 countries; Uganda,  South Africa [23] | F | F | F | N | F | F | F | F | F | F | F | F | F | P | F | F | F | F | P | F | P | F | F | F | N | F | F | F | 23 | 28 | 82.1% | High |
| Kim et al., 2013b,  EMENA region 20 countries; Algeria, Lebanon, Turkey [24] | F | F | F | N | F | F | F | F | F | F | F | F | F | P | F | F | F | F | P | F | P | F | F | F | N | F | F | F | 23 | 28 | 82.1% | High |
| Jit M et al., 2014,  England and Canada [7] | F | F | F | NA | F | F | P | NA | F | P | P | F | NA | NA | NA | F | F | P | P | F | N | F | F | F | NA | F | F | F | 16 | 22 | 72.7% | Moderate |
| Bonanni et al., 2015, Italy [49] | F | F | F | NA | F | F | P | NA | F | P | P | F | NA | NA | NA | F | F | P | P | F | N | F | F | F | NA | F | F | F | 16 | 22 | 72.7% | Moderate |
| Aljunid et al., 2016, Malaysia [41] | F | F | F | N | F | F | P | F | F | F | F | F | P | F | F | F | P | P | N | F | N | F | F | F | NA | F | F | F | 20 | 27 | 74.1% | Moderate |
| Setiawan et al., 2016a, Indonesia [42] | F | F | F | N | F | F | P | F | F | F | F | F | P | F | F | P | P | P | F | F | F | F | F | F | N | F | F | F | 21 | 28 | 75.0% | Moderate |
| Setiawan et al., 2016b, Indonesia [55] | F | F | F | NA | F | F | P | F | F | F | P | F | NA | F | F | F | F | P | P | F | N | F | F | F | NA | F | F | F | 20 | 25 | 80.0% | High |
| Vargas Parada and Veerman, 2016, Chile [33] | F | F | F | N | F | F | F | F | F | F | F | F | F | P | F | F | P | P | F | F | F | F | F | F | F | F | F | F | 24 | 28 | 85.7% | High |
| Kosen et al., 2017, Indonesia [43] | F | F | F | N | F | F | F | F | F | P | F | F | P | P | F | F | P | P | N | P | N | F | P | P | NA | F | P | P | 14 | 27 | 51.9% | Low |
| Mennini et al., 2017, Italy [44] | F | F | F | N | F | F | F | F | F | F | F | F | F | F | F | F | P | P | P | F | N | F | F | F | NA | F | F | F | 22 | 27 | 81.5% | High |
| Chesson et al., 2018,  United States [28] | F | F | F | N | F | F | F | F | F | F | F | F | F | F | F | F | F | P | F | F | F | F | F | F | F | F | F | F | 26 | 28 | 92.9% | High |
| Yaghoubi et al., 2018, Iran [32] | F | F | F | P | F | F | F | F | F | F | F | F | F | F | F | F | P | P | P | F | N | F | F | F | NA | F | F | F | 22 | 27 | 81.5% | High |
| Bahr et al., 2019, Lebanon [53] | F | F | F | NA | F | F | P | F | P | NA | NA | NA | NA | P | F | N | P | N | N | P | N | F | P | P | NA | F | F | F | 11 | 22 | 50.0% | Low |
| Datta et al., 2019, United Kingdom [10] | F | F | F | P | F | F | F | F | F | F | F | F | F | F | F | F | F | F | F | F | F | F | F | F | F | F | F | F | 27 | 28 | 96.4% | High |
| Lee et al., 2019, South Korea [30] | F | F | F | N | F | F | F | F | F | F | F | F | P | F | F | F | P | P | N | F | N | F | P | F | NA | F | F | F | 20 | 27 | 74.1% | Moderate |
| Messoudi et al., 2019, Morocco [45] | F | F | F | N | F | F | F | F | F | F | P | F | NA | F | F | F | F | P | N | F | N | F | P | F | NA | F | F | F | 20 | 26 | 76.9% | Moderate |
| Smith et al., 2019, Canada [55] | F | F | F | NA | F | F | F | F | F | F | P | F | NA | P | F | F | P | P | N | P | N | F | P | P | NA | F | F | F | 16 | 25 | 64.0% | Moderate |
| Tang et al., 2019, Taiwan [31] | F | F | F | N | F | F | F | F | F | F | F | F | F | F | F | F | P | P | F | F | F | F | F | F | N | F | F | F | 24 | 28 | 85.7% | High |
| Chauhan et al., 2020, India [46] | F | F | F | N | F | F | F | F | F | F | F | F | F | F | F | F | P | P | F | F | F | F | F | F | F | F | F | F | 25 | 28 | 89.3% | High |
| Kitano, 2022, Japan [56] | F | F | F | NA | F | F | F | F | F | F | P | F | F | F | F | F | P | P | N | F | N | F | P | F | NA | F | P | F | 19 | 26 | 73.1% | Moderate |
| Owusu-Edusei et al., 2022,  United Kingdom [25] | F | F | F | N | F | F | F | F | F | F | F | F | F | F | F | F | P | P | N | F | N | F | P | F | NA | F | F | P | 20 | 27 | 74.1% | Moderate |
| Sargazi et al., 2022, Iran [52] | F | F | F | NA | F | F | F | F | F | F | P | F | NA | F | F | F | P | P | N | F | N | F | P | F | NA | F | F | F | 19 | 25 | 76.0% | Moderate |
| Palmer et al., 2023, Japan [11] | F | F | F | P | F | F | F | F | F | F | F | F | F | F | F | F | P | P | P | F | P | F | P | F | F | F | F | P | 21 | 28 | 75.0% | Moderate |
| Slavkovsky et al., 2024, Ethiopia, Guyana, Rwanda, Sri Lanka, and Uganda [51] | F | F | F | NA | F | F | F | F | F | NA | NA | F | NA | F | F | F | P | P | N | F | N | F | P | F | NA | F | F | F | 18 | 23 | 78.3% | Moderate |
| Şahin and Karabey, 2025,  Türkiye (Turkey) [26] | F | F | F | P | F | F | F | F | F | F | F | F | P | F | F | F | F | P | N | F | N | F | P | F | NA | F | F | F | 21 | 27 | 77.8% | Moderate |
| Termrungruanglert et al., 2024, Thailand [57] | F | F | F | N | F | F | F | F | F | F | F | F | F | F | F | F | P | N | N | F | N | F | F | F | NA | F | F | P | 21 | 27 | 77.8% | Moderate |
| Gao et al., 2025,  Nigeria [47] | F | F | F | N | F | F | F | F | F | F | F | F | F | F | F | F | F | N | N | F | N | F | F | F | NA | P | F | P | 21 | 27 | 77.8% | Moderate |

***Coding:*** *F = fully reported; P = partially reported; N = not reported; NA = not applicable. CHEERS = Consolidated Health Economic Evaluation Reporting Standards. NA was used where a CHEERS item was not applicable to the design of the record, including contextual economic evidence records, epidemiological models without economic outcomes, cost-consequence analyses, cost-benefit analyses, number-needed-to-vaccinate analyses, fiscal net present value analyses, risk-benefit analyses, coverage-uptake impact analyses, and cost-of-delivery studies. This is a reporting-completeness assessment based on CHEERS 2022 and should not be interpreted as a methodological quality or risk-of-bias assessment. Item 25, effect of engagement, was coded conditionally on Item 21, approach to engagement: where no engagement was reported, Item 21 = N, Item 25 was coded NA; where engagement was fully or partially reported, Item 21 = F or P, Item 25 was coded F, P, or N according to whether its effect on the study approach or findings was reported. CHEERS 2022 was applied primarily to the 33 full comparative economic evaluations. Contextual economic evidence records were coded descriptively only where items were applicable and were not included in the primary reporting-completeness synthesis. Reporting-completeness bands were reviewer-defined as high ≥80%, moderate 60–79%, and low <60% of applicable items fully reported. These bands are not part of CHEERS 2022 and were used only for descriptive comparison.*

***Row shading:*** *Green shading indicates full comparative economic evaluations included in the primary synthesis. Amber shading indicates contextual economic evidence records included for contextual mapping only. Row shading is used only as a visual aid; the classification key should be used as the authoritative interpretation if colors are not visible in print or journal formatting.*
