# Supplementary material for: Methodological drivers, reporting completeness, and transferability in HPV vaccination economic evaluations: A scoping review
Source: PLoS One. 2026 Oct 14;21(10):e0360732. doi: 10.1371/journal.pone.0360732 (PMC13667800; doi:10.1371/journal.pone.0360732)
Supplement: Supplementary file 6 [file pone.0360732.s006.docx]

**S6 Table. Database-specific search strategies and administrative/search-filter criteria.**

*Full Boolean search strategies were executed across six databases: PubMed, Scopus, PsycINFO, Embase, Web of Science, and CINAHL. Each strategy was structured around three Population–Concept–Context (PCC) blocks: HPV vaccine terms, economic evaluation terms, and vaccination program/context terms. Searches covered January 1, 2003, to December 31, 2025, with the final search conducted on January 2, 2026. Searches were limited to English-language records. Publication-type exclusions were applied where database functionality allowed; remaining ineligible publication types were excluded during screening.*

| **Database** | **Concept Block** | **Search String** |
| --- | --- | --- |
| **PubMed**  *MeSH + Title/Abstract syntax* | Block 1: Population (HPV vaccine) | ("Papillomavirus Vaccines"[MeSH] OR "human papillomavirus"[Title/Abstract]  OR "human papilloma virus"[Title/Abstract] OR HPV[Title/Abstract]  OR "HPV vaccine"[Title/Abstract] OR "HPV vaccination"[Title/Abstract]  OR Gardasil[Title/Abstract] OR Cervarix[Title/Abstract]  OR "9vHPV"[Title/Abstract] OR "4vHPV"[Title/Abstract] OR "2vHPV"[Title/Abstract]  OR nonavalent[Title/Abstract] OR quadrivalent[Title/Abstract] OR bivalent[Title/Abstract]) |
|  | Block 2: Concept (economic evaluation) | AND  ("Costs and Cost Analysis"[MeSH] OR "Cost-Benefit Analysis"[MeSH]  OR "cost-effectiveness"[Title/Abstract] OR "cost effectiveness"[Title/Abstract]  OR "cost-utility"[Title/Abstract] OR "cost utility"[Title/Abstract]  OR "cost-benefit"[Title/Abstract] OR "cost benefit"[Title/Abstract]  OR "cost-consequence"[Title/Abstract] OR "cost consequence"[Title/Abstract]  OR "cost-minimization"[Title/Abstract] OR "cost minimisation"[Title/Abstract]  OR "economic evaluation"[Title/Abstract] OR "economic analysis"[Title/Abstract]  OR "economic model"[Title/Abstract] OR "decision model"[Title/Abstract]  OR ICER[Title/Abstract] OR QALY[Title/Abstract] OR DALY[Title/Abstract]  OR "budget impact"[Title/Abstract] OR "health technology assessment"[Title/Abstract] OR pharmacoeconomic*[Title/Abstract]) |
|  | Block 3: Context (vaccination program) | AND  (vaccin*[Title/Abstract] OR immuni*[Title/Abstract]  OR "immunization program"[Title/Abstract] OR "immunisation programme"[Title/Abstract]  OR "vaccination program"[Title/Abstract] OR "vaccination programme"[Title/Abstract]) |
|  | Filters and limits | AND English[Language]  AND ("2003/01/01"[Date - Publication] : "2025/12/31"[Date - Publication])  NOT (editorial[Publication Type] OR letter[Publication Type]  OR comment[Publication Type] OR news[Publication Type]) |
| **Scopus**  *TITLE-ABS-KEY syntax* | Block 1: Population (HPV vaccine) | ( TITLE-ABS-KEY ( "human papillomavirus" OR "human papilloma virus" OR HPV  OR "HPV vaccine" OR "HPV vaccination"  OR Gardasil OR Cervarix  OR "9vHPV" OR "4vHPV" OR "2vHPV" OR nonavalent OR quadrivalent OR bivalent ) ) |
|  | Block 2: Concept (economic evaluation) | AND  ( TITLE-ABS-KEY ( "cost-effectiveness" OR "cost effectiveness" OR "cost-utility" OR "cost utility"  OR "cost-benefit" OR "cost benefit" OR "cost-consequence" OR "cost-minimization" OR "cost minimisation"  OR "economic evaluation" OR "economic analysis" OR "economic model" OR "decision model"  OR ICER OR QALY OR DALY OR "budget impact" OR "health technology assessment" OR pharmacoeconomic* ) ) |
|  | Block 3: Context (vaccination program) | AND  ( TITLE-ABS-KEY ( vaccin* OR immuni* OR "vaccination program*" OR "vaccination programme*"  OR "immunization program*" OR "immunisation programme*" ) ) |
|  | Filters and limits | AND LANGUAGE ( english )  AND PUBYEAR > 2002 AND PUBYEAR < 2026  AND NOT ( DOCTYPE(ed) OR DOCTYPE(le) OR DOCTYPE(no) OR DOCTYPE(cp) ) |
| **Embase**  *EmTree + Title/Abstract (.ti,ab) syntax* | Block 1: Population (HPV vaccine) | ('papillomavirus vaccine'/exp OR 'human papillomavirus':ti,ab  OR 'human papilloma virus':ti,ab OR HPV:ti,ab  OR 'HPV vaccine':ti,ab OR 'HPV vaccination':ti,ab  OR Gardasil:ti,ab OR Cervarix:ti,ab  OR '9vHPV':ti,ab OR '4vHPV':ti,ab OR '2vHPV':ti,ab  OR nonavalent:ti,ab OR quadrivalent:ti,ab OR bivalent:ti,ab) |
|  | Block 2: Concept (economic evaluation) | AND  ('cost effectiveness analysis'/exp OR 'cost benefit analysis'/exp  OR 'cost utility analysis'/exp OR 'health economics'/exp  OR 'cost-effectiveness':ti,ab OR 'cost effectiveness':ti,ab  OR 'cost-utility':ti,ab OR 'cost utility':ti,ab  OR 'cost-benefit':ti,ab OR 'cost benefit':ti,ab  OR 'cost-consequence':ti,ab OR 'cost consequence':ti,ab  OR 'cost-minimization':ti,ab OR 'cost minimisation':ti,ab  OR 'economic evaluation':ti,ab OR 'economic analysis':ti,ab OR 'economic model':ti,ab  OR ICER:ti,ab OR QALY:ti,ab OR DALY:ti,ab  OR 'budget impact':ti,ab OR 'health technology assessment':ti,ab OR pharmacoeconomic*:ti,ab) |
|  | Block 3: Context (vaccination program) | AND  (vaccin*:ti,ab OR immuni*:ti,ab  OR 'vaccination program':ti,ab OR 'vaccination programme':ti,ab  OR 'immunization program':ti,ab OR 'immunisation programme':ti,ab) |
|  | Filters and limits | AND [english]/lim  AND [2003-2025]/py  NOT ('editorial'/it OR 'letter'/it OR 'note'/it OR 'conference abstract'/it) |
| **Web of Science**  *Topic Search (TS=) syntax* | Block 1: Population (HPV vaccine) | TS=("human papillomavirus" OR "human papilloma virus" OR HPV  OR "HPV vaccine" OR "HPV vaccination"  OR Gardasil OR Cervarix  OR "9vHPV" OR "4vHPV" OR "2vHPV" OR nonavalent OR quadrivalent OR bivalent) |
|  | Block 2: Concept (economic evaluation) | AND  TS=("cost-effectiveness" OR "cost effectiveness" OR "cost-utility" OR "cost utility"  OR "cost-benefit" OR "cost benefit" OR "cost-consequence" OR "cost-minimization" OR "cost minimisation"  OR "economic evaluation" OR "economic analysis" OR "economic model" OR "decision model"  OR ICER OR QALY OR DALY OR "budget impact" OR "health technology assessment" OR pharmacoeconomic*) |
|  | Block 3: Context (vaccination program) | AND  TS=(vaccin* OR immuni* OR "vaccination program*" OR "vaccination programme*"  OR "immunization program*" OR "immunisation programme*") |
|  | Filters and limits | Refined by: LANGUAGES = (English)  PUBLICATION YEARS = (2003-2025)  DOCUMENT TYPES = (Article)  [Ineligible publication types were excluded during screening  where database-level exclusion was not feasible.] |
| **PsycINFO**  *via Ovid (.ti,ab. syntax)* | Block 1: Population (HPV vaccine) | (("human papillomavirus" OR "human papilloma virus" OR HPV  OR "HPV vaccine" OR "HPV vaccination"  OR Gardasil OR Cervarix  OR "9vHPV" OR "4vHPV" OR "2vHPV"  OR nonavalent OR quadrivalent OR bivalent).ti,ab.) |
|  | Block 2: Concept (economic evaluation) | AND  (("cost-effectiveness" OR "cost effectiveness"  OR "cost-utility" OR "cost utility"  OR "cost-benefit" OR "cost benefit"  OR "cost-consequence" OR "cost consequence"  OR "cost-minimization" OR "cost minimisation"  OR "economic evaluation" OR "economic analysis" OR "economic model" OR "decision model"  OR ICER OR QALY OR DALY OR "budget impact"  OR "health technology assessment" OR pharmacoeconomic*).ti,ab.) |
|  | Block 3: Context (vaccination program) | AND  ((vaccin* OR immuni* OR "vaccination program*" OR "vaccination programme*"  OR "immunization program*" OR "immunisation programme*").ti,ab.) |
|  | Filters and limits | LIMIT to English language AND year = 2003-2025  NOT (editorial OR letter OR comment OR news).pt. |
| **CINAHL**  *EBSCO syntax (MH = Subject Heading; TI = Title; AB = Abstract)* | Block 1: Population (HPV vaccine) | (MH "Papillomavirus Vaccines+" OR TI "human papillomavirus" OR AB "human papillomavirus"  OR TI "human papilloma virus" OR AB "human papilloma virus"  OR TI HPV OR AB HPV  OR TI "HPV vaccine" OR AB "HPV vaccine" OR TI "HPV vaccination" OR AB "HPV vaccination"  OR TI Gardasil OR AB Gardasil OR TI Cervarix OR AB Cervarix  OR TI "9vHPV" OR AB "9vHPV" OR TI "4vHPV" OR AB "4vHPV" OR TI "2vHPV" OR AB "2vHPV"  OR TI nonavalent OR AB nonavalent OR TI quadrivalent OR AB quadrivalent  OR TI bivalent OR AB bivalent) |
|  | Block 2: Concept (economic evaluation) | AND  (MH "Costs and Cost Analysis+" OR MH "Cost Benefit Analysis"  OR TI "cost-effectiveness" OR AB "cost-effectiveness"  OR TI "cost effectiveness" OR AB "cost effectiveness"  OR TI "cost-utility" OR AB "cost-utility"  OR TI "cost utility" OR AB "cost utility"  OR TI "cost-benefit" OR AB "cost-benefit"  OR TI "cost benefit" OR AB "cost benefit"  OR TI "cost-consequence" OR AB "cost-consequence"  OR TI "cost consequence" OR AB "cost consequence"  OR TI "cost-minimization" OR AB "cost-minimization"  OR TI "cost minimisation" OR AB "cost minimisation"  OR TI "economic evaluation" OR AB "economic evaluation"  OR TI "economic analysis" OR AB "economic analysis"  OR TI "economic model" OR AB "economic model"  OR TI ICER OR AB ICER OR TI QALY OR AB QALY OR TI DALY OR AB DALY  OR TI "budget impact" OR AB "budget impact"  OR TI "health technology assessment" OR AB "health technology assessment" OR TI pharmacoeconomic* OR AB pharmacoeconomic*) |
|  | Block 3: Context (vaccination program) | AND  (TI vaccin* OR AB vaccin* OR TI immuni* OR AB immuni*  OR TI "vaccination program*" OR AB "vaccination program*"  OR TI "vaccination programme*" OR AB "vaccination programme*"  OR TI "immunization program*" OR AB "immunization program*"  OR TI "immunisation programme*" OR AB "immunisation programme*") |
|  | Filters and limits | Limiters:  — Language: English  — Publication Date: 2003-2025  — Exclude: Editorial, Letter, Commentary, News |

**Notes**

1. The PubMed strategy is the primary syntax; strategies for other databases are adapted to platform-specific syntax conventions (controlled vocabulary, wildcard, field-tag, and filter syntax).

2. Wildcard truncation (vaccin*, immuni*) captures vaccine/vaccination/vaccinated and immunisation/immunization/immunised/immunized to maximize sensitivity.

3. Both British (immunisation, minimisation, programme) and American (immunization, minimization, program) spelling variants are included in OR clauses to maximize retrieval across journals using either convention.

4. Free-text terms include both "human papillomavirus" (single-word) and "human papilloma virus" (spaced) variants, the abbreviation HPV, the phrases "HPV vaccine" and "HPV vaccination", product names (Gardasil, Cervarix), valency abbreviations (9vHPV, 4vHPV, 2vHPV), and product-type descriptors (nonavalent, quadrivalent, bivalent).

5. Outcome-metric keywords (ICER, QALY, DALY) and the broader term pharmacoeconomic* are included explicitly to capture economic-evaluation studies that may not use full descriptor phrases.

6. The date range begins on January 1, 2003 (to capture pre-licensure modeling studies anticipated before the first HPV vaccine licensure in 2006) and ends on December 31, 2025 (search cut-off date), with the final search conducted on January 2, 2026, to ensure completeness.

7. In Web of Science, Articles were retained as the document type at the database-filter stage. In databases where reliable publication-type exclusion was not feasible, remaining ineligible publication types were excluded during screening according to the eligibility criteria.

*Abbreviations: HPV, human papillomavirus; PCC, Population–Concept–Context; MeSH, Medical Subject Headings; TS, Topic Search; ICER, incremental cost-effectiveness ratio; QALY, quality-adjusted life year; DALY, disability-adjusted life year.*

**Supplementary Table S6b. Administrative and search-filter exclusions before title and abstract screening.**

| **Exclusion category** | **n** | **Criterion applied** | **Field(s) checked** | **Software/tool** | **Automated or manual** | **Verification procedure** |
| --- | --- | --- | --- | --- | --- | --- |
| Study design or publication-type mismatches | 19 | Records whose publication type or study design was ineligible (e.g., editorials, letters, notes, comments, conference items, or reviews) and not already removed by database-level publication-type filters. | Publication type; document-type metadata; title; abstract | EndNote 21; Covidence | Manual (manually verified) | Publication type read from database metadata and confirmed by a reviewer against the title and abstract; ambiguous cases were retained and carried forward to screening. |
| Topic or intervention mismatches | 24 | Records outside the HPV vaccination scope (e.g., non-HPV topics or non-vaccination interventions retrieved by broad search terms). | Title; abstract; keywords | EndNote 21; Covidence | Manual (manually verified) | Topic relevance judged by a reviewer from title, abstract, and keywords; records with any potential HPV vaccination economic content were retained for screening. |
| No identifiable economic evaluation component | 485 | Records with no cost or economic-evaluation content identifiable in the bibliographic fields (no cost-effectiveness, cost-utility, cost-benefit, cost-consequence, budget-impact, ICER, QALY, or DALY terms). | Title; abstract; keywords | EndNote 21; Covidence | Manual (manually verified) | Absence of any economic-evaluation content confirmed by reviewer inspection of title, abstract, and keywords; records with any potential economic content were retained for screening. |
| Other administrative or search-filter exclusions | 20 | Records not assignable to the preceding three categories, retained through database-export or search-filter artifacts (e.g., residual export or formatting artifacts). | Database metadata; bibliographic fields | EndNote 21; Covidence | Manual (manually verified) | Records inspected individually by a reviewer; the exact composition of this residual category was not separately cataloged in the study records (see note). |

***Note:*** *These four categories account for the 548 records removed after de-duplication and before title and abstract screening (19 + 24 + 485 + 20 = 548), corresponding to the counts reported in the manuscript and in Figure 1. Removals at this stage reflected record type, topic, or the complete absence of an economic component rather than a judgment on study quality; any record with potential economic evaluation content was retained and carried forward to title and abstract screening. Exclusions were verified manually within the reference-management and screening workflow; no automated text-mining or machine-classification algorithm was used. The exact composition of the residual “other administrative or search-filter exclusions” category (n = 20) was not separately cataloged in the study records.*
